# Bioreduction of *N*-(3-oxobutyl)heterocycles with flexible ring by yeast whole-cell biocatalysts

**DOI:** 10.1007/s00253-025-13486-2

**Published:** 2025-04-30

**Authors:** Máté Gergő Honvári, Bence Attila Kucsinka, Levente András Mócza, Pál Csuka, Viktória Bódai, László Poppe, Gábor Hornyánszky

**Affiliations:** 1 Department of Organic Chemistry and Technology, Faculty of Chemical Technology and Biotechnology, Budapest University of Technology and Economics, Műegyetem Rkp. 3, 1111 Budapest, Hungary; 2 Fermentia Microbiological Ltd, Berlini Út 47 - 49, 1049 Budapest, Hungary; 3 Witaria Ltd, Luther utca 4–6, 1087 Budapest, Hungary; 4 Biocatalysis and Biotransformation Research Centre, Faculty of Chemistry and Chemical Engineering, Babeș-Bolyai University of Cluj-Napoca, Arany János Str. 11, 400028 Cluj-Napoca-Napoca, Romania

**Keywords:** Alcohol dehydrogenase, Immobilization, Ketoreductase, Recyclability, Whole-cell bioreduction, Yeast

## Abstract

**Abstract:**

This study explored the bioreduction of *N*-(3-oxobutyl)heterocycles with (partially) saturated heterocyclic moieties using whole-cell forms of wild-type yeast strains and commercially available baker’s yeast (*Saccharomyces cerevisiae*). Eleven wild-type yeast strains and baker’s yeast were screened for ketoreductase activity on a series of five flexible *N*-heterocycles with prochiral carbonyl group in the *N*-(3-oxobutyl) substituent. Among the yeast strains tested, *Candida parapsilosis* (WY12) proved to be the most efficient biocatalyst in the bioreductions, resulting in the corresponding enantiopure alcohols—being promising chiral fragments with high level of drug-likeness—with good to excellent conversions (83–99%) and high enantiomeric excess (ee > 99%). Other strains, such as *Pichia carsonii* (WY1) and *Lodderomyces elongisporus* (WY2), also showed promising ketoreductase activities with certain substrates. After screening as lyophilized whole cells, *C. parapsilosis* cells were immobilized in the form of calcium, zinc, nickel, and copper alginate beads. The whole-cell immobilization enabled recycling, with considerable residual activity of the biocatalyst over multiple cycles. Additionally, the study explored the scalability of these bioreductions, with immobilized *C. parapsilosis* delivering promising results. The use of immobilized cells simplified the work-up process and resulted in chiral alcohols with similar or even higher conversions to those observed in the screening reactions. Molecular docking of the five flexible *N*-heterocycles with prochiral carbonyl group into the active site of the experimental structure of the carbonyl reductase of *C. parapsilosis* rationalized their biocatalytic behavior and confirmed the assigned (*S*)-configuration of forming enantiopure alcohols.

**Key points:**

• *Ketoreductase activity of eleven wild-type yeast strains and baker’s yeast were examined*.

• *Candida parapsilosis was subjected to whole-cell immobilization and recycling*.

• *Enantiopure alcohols with flexible N-heterocyclic units were produced at preparative scale*.

**Supplementary Information:**

The online version contains supplementary material available at 10.1007/s00253-025-13486-2.

## Introduction

Owing to the existing synthetic methods (e.g., cross-coupling reactions), most active pharmaceutical ingredients (APIs) are sp^2^-rich, rigid molecules. However it is well documented, that “3D” molecules, containing predominantly sp^3^ atoms are more successful as drug candidates at each stage of research, from discovery to registered drug (Lovering et al. 2009). Molecular shape and size are on the primary level in the hierarchy of molecular descriptors (Brüstle et al. 2002), and the diversity of the shape of molecules is also a prerequisite for the broad bioactivity of molecular libraries (Sauer and Schwarz 2003). Fragment-based drug discovery (FBDD), which employs a diverse set of chemical scaffolds and functionalities to cover chemical space, is an excellent tool for the identification of new chemical leads (Murray and Rees 2009). A study was conducted to assess every FDA-approved (United States Food and Drug Administration) small-molecule API (1086 unique drugs) and found that 84% of them (910 unique drugs) contain at least one nitrogen atom, while 59% (640 unique drugs) incorporate a nitrogen heterocycle. Piperazine was identified as the third most abundant nitrogen heterocycle, showing up in 59 of the analyzed APIs (Vitaku et al. 2014), for example, in the atypical antipsychotics Aripiprazole (Casey and Canal 2017) and Clozapine (Bhosle et al. 2023) or in the second-generation antihistamine Cetirizine (Curran et al. 2004). Chiral compounds are important intermediates and building blocks in the synthesis of APIs, where enantiopurity is of prime importance due to the strict regulations of pharmaceutical authorities. Asymmetric secondary hydroxyl groups show up in numerous chiral drugs, e.g., in the high blood cholesterol medication Ezetimibe (Singh et al. 2009), in the beta blocker Carvedilol (Ettireddy et al. 2017), or in the serotonin-norepinephrine reuptake inhibitor Duloxetine (Ren et al. 2015). In recent years, the application of ketoreductases (KREDs, also called alcohol dehydrogenases, ADHs) in the industrial-scale synthesis of chiral alcohols has undergone a revolutionary change (Huisman et al. 2010). For a KRED-catalyzed process to be viable, an efficient cofactor regeneration system is required, so that the expensive cofactor can be recycled (Matsuda et al. 2009). Numerous cofactor regeneration systems have been described so far, but one of the most straightforward options is to utilize the same KRED in a whole-cell system with inexpensive and easy-to-handle isopropyl alcohol as the co-substrate. Additional advantages of this method are easy scalability and applicability to both NADH- and NADPH-dependent enzymes; however, a disadvantage is that it may be necessary to remove the resulting acetone from the reaction mixture in order to shift the equilibrium (Huisman et al. 2010). Whole-cell wild-type yeast strains serve as valuable catalysts for biocatalytic ketoreductions (Goldberg et al. 2007) due to their highly efficient cofactor regeneration. Commonly used strains include *Candida* (e.g., *Candida parapsilosis*, *Candida albicans*, and *Candida norvegica*), *Lodderomyces* (e.g., *Lodderomyces elongisporus*), and *Pichia* (*Pichia carsonii*). These strains usually contain KREDs that typically obey Prelog’s rule (Prelog 1964) and catalyze the production of (*S*)-alcohols with high enantioselectivity from a wide variety of ketones (Contente et al. 2014, 2016a, 2016b; Jakoblinnert et al. 2011, 2012, 2013). Baker’s yeast (*Saccharomyces cerevisiae*) has also been utilized as a whole-cell biocatalyst in the bioreduction of prochiral ketones, yielding products with high conversions and enantiomeric excesses (Ren et al. 2021). The obtained hydroxyl functionalities can be used to incorporate drug-like fragments, resembling building blocks, into the target structure during the synthesis of pharmaceutically relevant molecules.

**Fig. 1:**
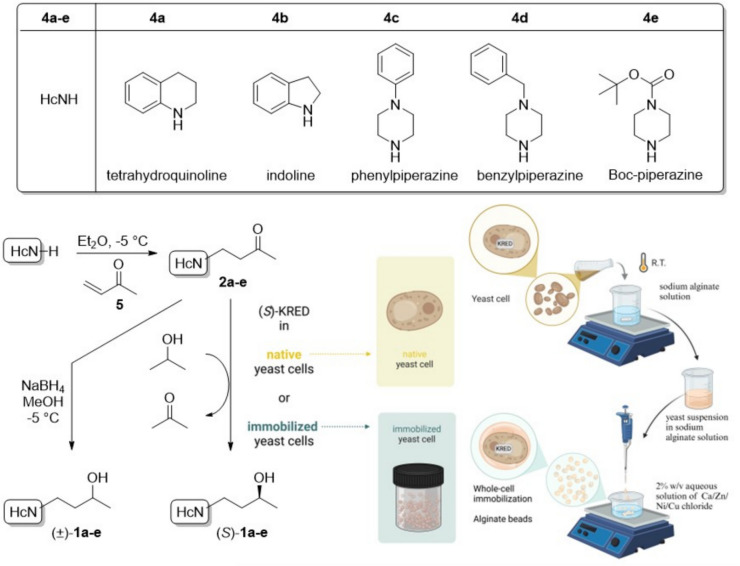
Synthesis of *N*-(3-oxobutyl)heterocycles with flexible rings **2a-e** and their chemical and bioreduction to racemic and optically active alcohols (±)-**1a-e** and (*S*)-**1a-e**, respectively. (*S*)-KRED-containing yeast cells were investigated in the bioreductions as lyophilized cell or as alginate bead immobilized forms (Created in BioRender. Bódai, V. (2024) https://BioRender.com/t33n319)

The objective of this research is to extend the synthetic toolkit of chiral “3D” heterocycles containing asymmetric alcohol functions that can be employed in fragment-based drug discovery (FBDD). To do so, the present study aimed to develop biocatalytic asymmetric bioreductions of prochiral ketones with flexible *N*-heterocycles (Fig. 1), which is surprisingly rarely exemplified in this field, mediated by yeast cells.

## Materials and methods

### Chemicals

The *N*-(3-oxobutyl)heterocycles (tetrahydroquinoline **4a**, indoline **4b**, phenylpiperazine **4c**, benzylpiperazine **4d**, *N-*Boc-piperazine **4e**), the reagents, and solvents were purchased from Sigma-Aldrich (St. Louis, MO, USA), Merck KGaA (Darmstadt, Germany), or Molar Chemicals (Halásztelek, Hungary).

### Biocatalysts

Lyophilized whole-cell yeasts investigated in this study [*Pichia carsonii* (WY1), *Lodderomyces elongisporus* (WY2), *Wickerhamomyces subpelliculosus* (WY3), *Candida norvegica* (WY4), *Cryptococcus curvatus* (WY5), *Debaryomyces hansenii* (WY6), *Candida guillermondi* (WY7), *Debaryomyces fabrii* (WY11), *Candida parapsilosis* (WY12), *Hanseniaspora uvarum* (WY33), and *Rhodotorula glutinis* (WY51)] originated from Witaria Culture Collection (Budapest, Hungary). The *Candida parapsilosis* WY12 strain is available from National Collection of Agricultural and Industrial Microorganisms Hungarian University of Agriculture and Life Sciences (NCAIM) under accession number Y.02342. The fermentation and lyophilization of the yeast strains were described earlier (Erdélyi et al. 2006; Bódai et al. 2016; Csuka et al. 2021). Commercially available baker’s yeast (*Saccharomyces cerevisiae*) in both wet (BY; Budafoki baker’s yeast, Lesaffre Hungary Ltd.) and dried (BYD; Dr. Oetker Instant baker’s yeast, Dr. Oetker Hungary Ltd.) form was purchased in a local grocery store. Amano Lipase PS from *Burkholderia cepacia* was purchased from Sigma-Aldrich (St. Louis, MO, USA).

### Analytical methods

NMR spectra were recorded in the indicated deuterated solvents (CDCl_3_ in the case of free bases and D_2_O in the case of piperazine dihydrochloride salts) on a Bruker DRX-500 spectrometer operating at 500 MHz for ^1^H and 126 MHz for ^13^C. NMR signals were given in ppm on the δ scale. Infrared (IR) spectra were recorded on a Bruker ALPHA FT-IR spectrometer (in ATR mode), and wavenumbers of bands were listed in cm. Optical rotations were measured on a Perkin-Elmer 241 polarimeter at the 578 nm line of mercury and at the D-line of sodium (589 nm). The polarimeter was calibrated with measurements of both enantiomers of menthol. Thin layer chromatography (TLC) was carried out on precoated TLC ALUGRAM® Xtra SIL G/UV254 sheets (Macherey–Nagel); spots were visualized under UV light (254 or 365 nm) and with phosphomolybdic acid staining. Column chromatography was carried out with Gerduran® Si 60 (Merck) silica gel. Gas chromatography (GC) was used to determine the conversion and the enantiomeric excess of the bioreductions from ketones **2a-e.** GC analyses were performed with an Agilent 4890 GC equipped with FID detector and Hydrodex β−6TBDM column [25 m × 0.25 mm × 0.25 μm film with heptakis-(2,3-di-*O*-methyl-6-*O*-t-butyldimethylsilyl)-β-cyclodextrin; Macherey & Nagel (Düren, Germany); H_2_ carrier gas (injector: 250 °C, detector: 250 °C, head pressure: 12 psi, split ratio: 50:1)] and an Agilent 5890 GC equipped with FID detector and HP-5 column [30 m × 0.25 mm × 0.25 μm film with (5%-phenyl)-methylpolysiloxane; Agilent Technologies (Santa Clara, California, U.S.); H_2_ carrier gas (injector: 250 °C, detector: 250 °C, head pressure: 15 psi, split ratio: 50:1)]. The temperature programs and retention times are included in the Supplementary Information (Table S1).

To determine the conversion of the bioreductions, samples (100 μL) were taken from the reaction mixtures. These samples were extracted with EtOAc (1000 μL), and the organic layer was then separated and dried over sodium sulfate. The decanted EtOAc-extracts were analyzed directly. Since the corresponding alcohols and ketones have the same number of carbon atoms, and very similar molar masses, the molar response factors of alcohols to ketones (GC equipped with FID detector) were assumed to be 1.

To determine the enantiomeric excess, freshly distilled acetic anhydride (10 μL) was added to the dry EtOAc extract of the sample, and acylation was conducted at room temperature for 1 min to give the desired acetylated alcohols. The excess of the acylating agent was quenched with water (10 μL, room temperature, 1 min quenching time), the organic phase was dried over sodium sulfate, and the decanted EtOAc-extracts were analyzed directly.

As experiments performed with the model compound **2a** in triplicates indicated a standard variation within 2%, further experiments were performed as single series.

### Synthesis of the substrates and reference compounds

The synthesis and spectral data of *N*-(3-oxobutyl)heterocycles **2a-e** (Silva et al. 2023; Ouyang et al. 2022; Kilic et al. 2012; Roth and Mühlenbruch 1970; Desantis et al. 2022) and racemic *N*-(3-hydroxybutyl)heterocycles (±)-**1a-e** (Deb et al. 2013; Nakamura et al. 2015; Zhu et al. 2024) can be found in the Supplementary Information (Sects. 1.1.1. and 1.1.2., respectively).

### Screening the bioreduction of ketones 2a-e to (*S*)-alcohols (*S*)−1a-e

In a 4 mL vial, lyophilized native yeast cells (30 mg) were suspended in sodium phosphate buffer (910 μL, pH = 7.0, 64 mM). To the suspension were added the cosubstrate 2-propanol (40 μL) and a solution of the ketone substrate (50 μL from a *c* = 1 M stock solution in DMSO (substrates **2a** and **2b**) or in the same phosphate buffer (substrates **2c**, **2d** and **2e**)). This way the mixture had a final volume of 1000 μL, a cosubstrate concentration of 4 V/V%, and a substrate concentration of 50 mM (9.5 to 12.8 mg mL^−1^ depending on the molar mass of the substrate). The resulting mixture was shaken at 700 rpm for 24 h at 30 °C. After 24 h, a sample (100 μL) was taken from the reaction mixture. The same method was employed to investigate commercial baker’s yeast (*Saccharomyces cerevisiae*) using 100 mg of baker’s yeast in wet form (BY) or 30 mg of lyophilized baker’s yeast (BYD) being suspended in the buffer.

### Immobilization of *Candida parapsilosis* WY12 cells by alginate entrapment

Immobilization of the best performing yeast strain *Candida parapsilosis* (WY12) in the form of calcium alginate beads was performed according to a previous study (Padhi and Chadha 2005). Briefly, in a 100 mL flask containing distilled water (40 mL), sodium alginate (0.8 g) was added, and the suspension was stirred at 50 °C for 1 h. After cooling to room temperature, a suspension of lyophilized *C. parapsilosis* (WY12, 4.0 g) in distilled water (20 mL) was added to the sodium alginate solution. After stirring for 1 h, the forming slurry was added dropwise to a pre-chilled 2% w/v CaCl_2_ solution resulting in the formation of calcium alginate beads with diameter of 1.5–2.0 mm. After maturing the beads in the CaCl_2_ solution for 12 h, washing was performed with Tris buffer (3 × 100 mL, pH = 7.5, 50 mM) resulting in 36 g of Im-WY12-Ca (wet weight) entrapping 4.0 g of lyophilized *C. parapsilosis* cells. Zinc, nickel, and copper alginate beads of *Candida parapsilosis* (WY12) were prepared analogously, using a 2% w/v aqueous solution of ZnCl_2_, NiCl_2_, and CuCl_2_, respectively.

### Bioreductions with immobilized *Candida parapsilosis* WY12

In a 4 mL vial, alginate beads containing immobilized yeast cells (Im-WY12-Ca, Im-WY12-Zn, Im-WY12-Ni or Im-WY12-Cu, 300 mg) were suspended in Tris buffer (950 μL, pH = 7.0, 25 mM). To the mixture were added the cosubstrate 2-propanol (40 μL) and a solution of the ketone substrate (10 μL from a *c* = 1 M stock solution in DMSO (substrates **2a** and **2b**) or in the same Tris buffer (substrates **2d** and **2e**)). This way the mixture had a final volume of 1000 μL, a cosubstrate concentration of 4 V/V% and a substrate concentration of 10 mM (1.9 to 2.6 mg/mL depending on the molar mass of the substrate). The resulting mixture was shaken at 700 rpm for 24 h at 30 °C. The conversions of bioreductions were determined by GC analysis of samples (100 μL) taken from the reaction mixtures at 2, 4, 8, and 24 h (**2a** and **2b**) or after 24 h (**2d** and **2e**).

### Recyclability of the immobilized *Candida parapsilosis* WY12

In a 4 mL vial, alginate beads containing immobilized yeast cells (Im-WY12-Ca or Im-WY12-Zn, 300 mg) were suspended in Tris buffer (950 μL, pH = 7.0, 25 mM). To the mixture were added the cosubstrate 2-propanol (40 μL) and a solution of the substrate 4-(indolin-1-yl)butan-2-one **2b** (10 μL from a *c* = 1 M stock solution in DMSO). This way the mixture had a final volume of 1000 μL, a cosubstrate concentration of 4 V/V%, and a substrate concentration of 10 mM (1.9 mg/mL). The resulting mixture was shaken at 700 rpm for 2 h at 30 °C. After 2 h, a sample (100 μL) was taken from the reaction mixture and analyzed as described before. The remainder of the reaction mixture was pipetted off the alginate beads. The biocatalyst was washed three times with 1 mL of Tris buffer, then shaken in Tris buffer for 15 min (1 mL, 700 rpm, 30 °C). After thorough washing, the biocatalyst was tested in a next cycle of reaction (the process was repeated until a total of six times).

### Bioreduction of ketones 2a-c, 2e to (*S*)-alcohols (*S*)−1a-c, (*S*)−1e at preparative scale

#### Preparative scale bioreductions with lyophilized *C. parapsilosis* (WY12) cells

In a 20 mL vial, the lyophilized cells (WY12, 150 mg) were suspended in sodium phosphate buffer (4550 μL, pH = 7.0, 64 mM). To the suspension were added the cosubstrate 2-propanol (200 μL) and a solution of the ketone substrate (250 μL from a *c* = 1 M stock solution in DMSO (substrates **2a** and **2b**) or in the same phosphate buffer (substrate **2c**)). This way the mixture had a final volume of 5000 μL, a cosubstrate concentration of 4 V/V%, and a substrate concentration of 50 mM (9.5 to 11.6 mg mL^−1^ depending on the molar mass of the substrate). The resulting mixture was shaken at 450 rpm for 24 h at 30 °C. After 24 h, the mixture was allowed to separate, and the supernatant was extracted with EtOAc (3 × 5 mL). The combined organic layers were dried over sodium sulfate and evaporated in vacuum. The crude residue was purified by preparative TLC (**2a-b**, eluent: hexane/EtOAc 10:4; **2c**, eluent: CH_2_Cl_2_/MeOH/NH_4_OH 10:1:0.2) to give the desired alcohols (*S*)-**1a-c** (Table 4, entries 1–3).

### Preparative scale bioreductions with immobilized whole-cells of *C. parapsilosis* in alginate beads (Im-WY12-Zn) 

In a 100 mL bottle, alginate beads containing immobilized yeast cells (Im-WY12-Zn, 7.5 g) were suspended in Tris buffer (23.8 mL, pH = 7.0, 25 mM). To the mixture were added the cosubstrate 2-propanol (1000 μL) and a solution of the ketone substrate (250 μL from a *c* = 1 M stock solution in DMSO (substrates 2**a** and 2**b**) or in the same Tris buffer (substrate **2e**)). This way the mixture had a final volume of 25.0 mL, a cosubstrate concentration of 4 V/V%, and a substrate concentration of 10 mM (1.9 to 2.6 mg/mL depending on the molar mass of the substrate). The resulting mixture was shaken at 300 rpm for 2 h (**2b**), 4 h (**2a**), or 24 h (**2e**) at 30 °C. After the appropriate reaction time, the biocatalyst was filtered and washed with 10 mL of Tris buffer. The aqueous phase was extracted with 3 × 25 mL of EtOAc (**2a**, **2b**) or dichloromethane (**2e**). The combined organic layers were dried over sodium sulfate and evaporated in vacuo. The crude residue was purified by preparative TLC (**2a-b**, eluent: hexane/EtOAc 10:4) or by column chromatography over silica gel (**2e**, eluent: CH_2_Cl_2_/MeOH/NH_4_OH 10:1:0.2) to give the desired alcohols (*S*)−**1a-b**; (*S*)−**1e** (Table 4, entries 4–6).

The spectral data of the enantiopure alcohol products (Silva et al. 2023; Xu et al. 2020) can be found in the Supplementary Information (Sect. 1.1.3.).

### Absolute configuration assignment to the enantiomers (*S*)−1a-e

*Modeling the possible arrangement of the N-(3-oxobutyl)heterocycles 2a-e in the active site of the alcohol dehydrogenase of Candida parapsilosis*: The docking was performed by using Autodock VINA (Trott & Olson 2010; Eberhardt et al. 2021) implemented in PyMOL (DeLano 2002) by the DockingPie plugin (Rosignoli & Paiardini 2022) using the experimental structure of the carbonyl reductase from *C. parapsilosis* containing NADH cofactor (PDB ID: 4 C4O; Man et al. 2014). The structures of the *N*-(3-oxobutyl)heterocycles **2a-e** were generated in HyperChem (HyperChem 2009). After importing receptor and ligand structures from PyMOL into the DockingPie, *H*-atoms were added to the enzyme, the bonds and *H*-atoms in the dihydropyridine moiety of NADH were edited by PyMOLs tools, and a grid was set (centered at the midpoint of a cavity above the dihydropyridine part of NADH surrounded by residues of chain B of the trimeric 4C4O). Five docking runs were performed resulting in 20 poses for each run. Those docking poses with the best docking scores (*ds*) were considered as the best reactive arrangement in which the carbonyl *C*-atom of the ketone **2a-e** was in close vicinity (< 4.0 Å) of the *pro-R H*-atom of the dihydropyridine moiety of NADH (Run 3, Pose 1, *ds* = − 6.838 kcal/mol for **2a**; Run 4, Pose 3, *ds* = − 6.377 kcal/mol for **2b**; Run 1, Pose 3, *ds* = − 5.954 kcal/mol for **2c**; Run 4, Pose 4, *ds* = − 5.593 kcal/mol for **2d**; Run 4, Pose 2, *ds* = − 5.565 kcal/mol for **2e**).

## Results

The synthesis of pharmaceutically relevant prochiral *N*-(2-oxopropyl)heterocycles with flexible rings has previously been studied (Lakó et al. 2021), but the bioreduction of these compounds to enantiopure chiral alcohols mediated by KREDs represents an extended area of research. Three of the five heterocyclic rings studied in this research are piperazine-based (phenylpiperazine (**4c**), benzylpiperazine (**4d**), and Boc-piperazine (**4e**)), while the remaining two are partially saturated, fused rings (tetrahydroquinoline (**4a**) and indoline (**4b**)) (Fig. 1). These heterocyclic rings can be considered as 3D fragments, as they contain multiple sp^3^-hybridized carbon atoms, which result in a non-planar structure. In summary, the present study aimed to (1) assess the ketoreductase activity of numerous wild yeast strains and baker’s yeast on a set of prochiral *N*-(3-oxobutyl)heterocycles with flexible rings, (2) subject the best performing strain to whole-cell immobilization in the form of alginate beads created with various metal ions, (3) examine the recyclability of the aforementioned immobilized biocatalysts, (4) perform bioreductions at preparative scale to isolate and characterize the enantiopure products, and (5) ascertain the absolute configuration of the forming alcohol enantiomers with the help of suitable molecular modeling methods.

In this study, lyophilized cells of eleven yeast strains from the Witaria culture collection [*Pichia carsonii* (WY1), *Lodderomyces elongisporus* (WY2), *Wickerhamomyces subpelliculosus* (WY3), *Candida norvegica* (WY4), *Cryptococcus curvatus* (WY5), *Debaryomyces hansenii* (WY6), *Candida guillermondi* (WY7), *Debaryomyces fabrii* (WY11), *Candida parapsilosis* (WY12), *Hanseniaspora uvarum* (WY33), and *Rhodotorula glutinis* (WY51)] and commercially available baker’s yeast (*Saccharomyces cerevisiae*, in both wet (BY) and dried (BYD) forms) were investigated as biocatalysts in the bioreduction of a series of ketones with various (partially) saturated heterocyclic moieties {**2a**: 4-(3,4-dihydroquinolin-1(2*H*)-yl)butan-2-one, **2b**: 4-(indolin-1-yl)butan-2-one, **2c**: 4-(4-phenylpiperazin-1-yl)butan-2-one, **2 d**: 4-(4-benzylpiperazin-1-yl)butan-2-one, **2e**: *tert*-butyl 4-(3-oxobutyl)piperazine-1-carboxylate} (Fig. 1). The prochiral *N*-(3-oxobutyl)heterocycles with flexible rings **2a-e** were prepared by aza-Michael addition reaction of the corresponding (partially) saturated heterocycle **4a-e** and methyl vinyl ketone **5** (Fig. 1), resulting in moderate to excellent yields (52–98%). Racemic alcohols as standards (±)-**1a-e** for setting up the enantioselective GC analysis were prepared by sodium borohydride reduction of the corresponding ketones **2a-e** (Fig. 1), resulting in good to excellent yields (71–92%).

### Initial screening reactions

The goal of this study was to screen a diverse range of wild-type yeast strains to gain enantiopure alcohols by bioreductions from a selection of ketones comprising flexible *N*-heterocyclic parts. Previously, a wider selection of yeast strains was examined for bioreduction of various substrates in our research group (Erdélyi et al. 2006; Bódai et al. 2016; Nagy-Győr et al. 2019; Csuka et al. 2021). For example, *Debaryomyces hansenii* (WY6) was utilized for the stereoselective production of (*S*)−1-aralkylethanols (Erdélyi et al. 2006). Furthermore, *Wickerhamomyces subpelliculosus* (WY3) was used to transform several simple ketones and a ketone with aryl and cyclopropyl moieties (Bódai et al. 2016). Later, *Pichia carsonii* (WY1), *Lodderomyces elongisporus* (WY2), *Candida norvegica* (WY4), *Candida guillermondi* (WY7), *Debaryomyces fabrii* (WY11), and *Candida parapsilosis* (WY12) were tested in the bioreduction of model ketones with different molecular properties (Nagy-Győr et al. 2019; Csuka et al. 2021). Thus, the most efficient strains from our previous studies were selected to test the bioreduction of *N*-(3-oxobutyl)heterocycles with flexible ring, which was the goal of the present study.

First, the lyophilized samples of eleven yeast strains from the Witaria culture collection and the commercially available yeast, baker’s yeast (*Saccharomyces cerevisiae*) (Table 1), were screened for ketoreductase activity toward the target substrates **2a-e**.

**Table 1:** List of the screened yeast strains

Code	Yeast strain
WY1	*Pichia carsonii*
WY2	*Lodderomyces elongisporus*
WY3	*Wickerhamomyces subpelliculosus*
WY4	*Candida norvegica*
WY5	*Cryptococcus curvatus*
WY6	*Debaryomyces hansenii*
WY7	*Candida guillermondi*
WY11	*Debaryomyces fabrii*
WY12	*Candida parapsilosis*
WY33	*Hanseniaspora uvarum*
WY51	*Rhodotorula glutinis*
BY	Baker’s yeast (*Saccharomyces cerevisiae*)
BYD	Baker’s yeast (*Saccharomyces cerevisiae*, dry)

The initial screening (performed by thin layer chromatography (TLC)) revealed five strains [*Pichia carsonii* (WY1), *Lodderomyces elongisporus* (WY2), *Candida norvegica* (WY4), *Candida parapsilosis* (WY12) and *Rhodotorula glutinis* (WY51)] with detectable ketoreductase activity on the target substrates **2a-e** (Table 2). Three strains (WY1, WY2, and WY12) could produce the desired alcohol (*S*)−**1a-e** from all five tested substrates **2a-e**. The yeast strain WY4 showed activity only with substrates **2a**, **2b**, **2d**, while WY51 converted only **2a** and **2b** to the corresponding alcohol products. Both the wet and dry form of baker’s yeast (*Saccharomyces cerevisiae*) exhibited limited activity with substrates **2b**, **2c**, **2d**.

**Table 2:** TLC-based screening of several yeasts for bioreduction of ketones **2a-e** (50 mM **2a-e**, 3:1 yeast:substrate ratio, 4 V/V% *i*-PrOH, pH = 7.0, 30 °C, 24 h; the substrate-yeast strain combinations that exhibited detectable activity are marked with “** + **”; inactive combinations are denoted with “-”)

Substrate	Yeast strain												
	WY1	WY2	WY3	WY4	WY5	WY6	WY7	WY11	WY12	WY33	WY51	BY	BYD
**2a**	** + **	** + **	-	** + **	-	-	-	-	** + **	-	** + **	-	-
**2b**	** + **	** + **	-	** + **	-	-	-	-	** + **	-	** + **	** + **	** + **
**2c**	** + **	** + **	-	-	-	-	-	-	** + **	-	-	** + **	** + **
**2d**	** + **	** + **	-	** + **	-	-	-	-	** + **	-	-	** + **	** + **
**2e**	** + **	** + **	-	-	-	-	-	-	** + **	-	-	-	-

Because the TLC-based screen could not provide information on the selectivity of the various substrate-yeast strain combinations, the conversion and the enantiomeric composition of the alcohol products of bioreductions with conversions exceeding 10% were determined by GC analysis of samples taken from the positive screen reaction after 24 h (Table 3).

**Table 3:** Conversion and enantiomeric excess data of the bioreduction reactions of **2a-e** with yeast whole cells (WY1, WY2, WY4, WY12, WY51, BY, BYD) as biocatalysts (50 mM **2a-e**, 3:1 yeast:substrate ratio, 4 V/V% *i*-PrOH, pH = 7.0, 30 °C, 24 h)

Substrate	Yeast strain	Conversion [%]	Enantiomeric excess [%]
**2a**	WY1	75	> 99, (*S*)
	WY2	80	> 99, (*S*)
	WY4	9	n.d
	WY12	83	> 99, (*S*)
	WY51	24	74, (*R*)
**2b**	WY1	67	> 99, (*S*)
	WY2	72	> 99, (*S*)
	WY4	47	> 99, (*S*)
	WY12	93	> 99, (*S*)
	WY51	54	89, (*R*)
	BY	4	n.d
	BYD	6	n.d
**2c**	WY1	91	> 99, (*S*)
	WY2	98	> 99, (*S*)
	WY12	> 99	> 99, (*S*)
	BY	1	n.d
	BYD	2	n.d
**2d**	WY1	2	n.d
	WY2	3	n.d
	WY4	3	n.d
	WY12	3	> 99, (*S*)
	BY	6	n.d
	BYD	1	n.d
**2e**	WY1	8	n.d
	WY2	11	> 99, (*S*)
	WY12	20	> 99, (*S*)

*n.d.* not determined.

The highest activities were observed in the case of substrate **2c**, 4-(4-phenylpiperazin-1-yl)butan-2-one, resulting in the complete conversion of the prochiral substrate to enantiopure alcohol (remaining ketone substrate below the detection limit of the GC) utilizing yeast strain *C. parapsilosis* (WY12), with *L. elongisporus* (WY2) performing almost as well (98% conversion). In contrast, the lowest activities were observed with substrate **2d**, 4-(4-benzylpiperazin-1-yl)butan-2-one, reaching only a maximum of 6% with the wet form of baker’s yeast (*S. cerevisiae*). Substrates **2a**, 4-(3,4-dihydroquinolin-1(2*H*)-yl)butan-2-on and **2b**, 4-(indolin-1-yl)butan-2-on resulted in high conversions (> 70%), while the conversion of substrate **2e**, *tert*-butyl 4-(3-oxobutyl)piperazine-1-carboxylate to the corresponding enantiopure alcohol reached only a maximum of 20% (with *C. parapsilosis* (WY12)) under the above specified conditions.

In the case of *P. carsonii* (WY1), *L. elongisporus* (WY2), *C. norvegica* (WY4), and *C. parapsilosis* (WY12), high enantiotopic selectivities were observed in all bioreductions resulting in virtually enantiopure alcohols with enantiomeric excess exceeding 99%. These biocatalysts exhibited the usual Prelog selectivity, leading to enantiopure (*S*)-alcohols. On the other hand, *R. glutinis* (WY51) exhibited anti-Prelog selectivity, yielding (*R*)-alcohols with 74% ((*R*)-**1a**, 4-(3,4-dihydroquinolin-1(2*H*)-yl)butan-2-ol) and 89% ((*R*)-**1b**, 4-(indolin-1-yl)butan-2-ol) enantiomeric excess. The enantiotopic selectivity of baker’s yeast (*S. cerevisiae*) was not investigated, due to low conversion values (< 10%).

Neither the wet nor the dry form of baker’s yeast (*Saccharomyces cerevisiae*) proved to be useful biocatalysts for bioreductions of ketones **2a-e**, resulting in less than 10% conversion after 24 h (6% from **2b** with BYD or from **2d** with BY as the best). Overall, *C. parapsilosis* (WY12) exhibited the best performance among the examined yeast strains, while *P. carsonii* (WY1) and *L. elongisporus* (WY2) could also be considered as useful options. *C. norvegica* (WY4) was partially useful as this yeast could not convert ketones **2c,e**. The yeast *R. glutinis* (WY51) carried out bioreduction with only two ketones (**2a,b**) with moderate conversion and non-exclusive anti-Prelog enantiopreference.

### Bioreductions with immobilized yeasts

**Fig. 2:**
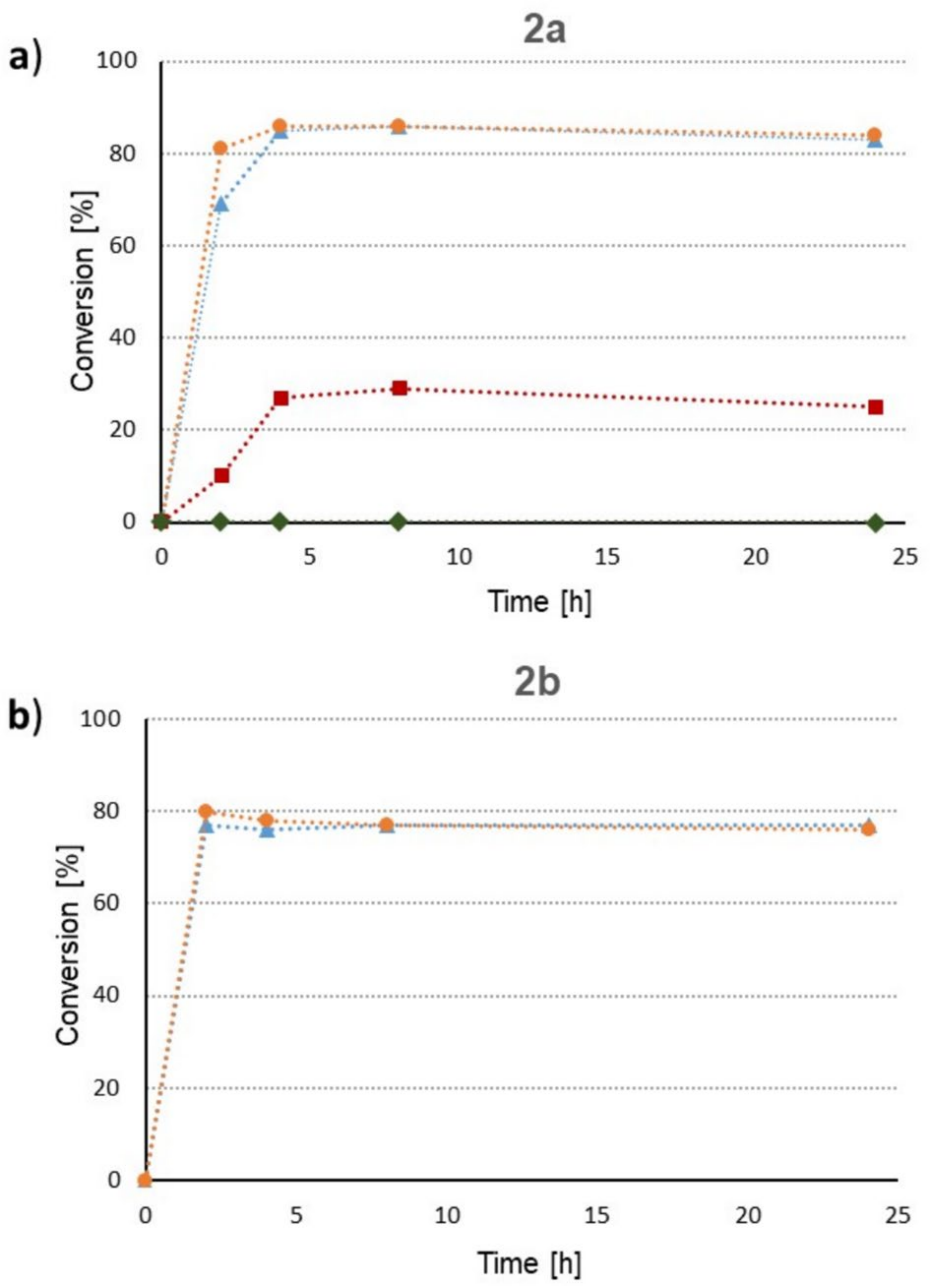
Bioreductions of (**a**) 4-(3,4-dihydroquinolin-1(2*H*)-yl)butan-2-one **2a** and (**b**) 4-(indolin-1-yl)butan-2-one **2b** with the immobilized forms of the best performing yeast strain (*Candida parapsilosis* WY12:  — Im-WY12-Ca;  — Im-WY12-Zn;  — Im-WY12-Ni;  — Im-WY12-Cu) and of (**b**) 4-(indolin-1-yl)butan-2-one **2b** with Im-WY12-Ca (  ) and Im-WY12-Zn (  ). Conditions: 10 mM **2a,b**; ~ 15:1 yeast:substrate ratio, 4 V/V% *i*-PrOH, pH = 7.0, 30 °C; ee_(*S*)-**1a,b**_ > 99% in all cases)

Following the initial screening, the most versatile *C. parapsilosis* (WY12) cells were subjected to whole-cell immobilization according to an alginate entrapment methodology applied in a previous study (Padhi and Chadha 2005). In this work, not only calcium alginate beads were created to immobilize *C. parapsilosis* (WY12) cells, but Zn^2+^, Ni^2+^, and Cu^2+^ ion cross-linked alginate beads as well. The KRED activity of these immobilized biocatalysts was first tested on 4-(3,4-dihydroquinolin-1(2*H*)-yl)butan-2-one **2a** as substrate (Fig. 2a), then the two best performing alginate entrapped WY12 forms were further tested on 4-(indolin-1-yl)butan-2-one **2b** as substrate (Fig. 2b).

Im-WY12-Cu exhibited no observable KRED activity with the substrate **2a**, most probably due to detrimental effect of the Cu^2+^ ion on the KRED enzyme of *C. parapsilosis*. Im-WY12-Ni showed quite moderate KRED activity, reaching only 25% conversion after 24 h. The biocatalysts Im-WY12-Ca and Im-WY12-Zn exhibited high ketoreductase activity, resulting in maximum conversion (85% and 86%) even after 4 h of reaction time. Based on these results, only Im-WY12-Ca and Im-WY12-Zn were further tested on substrate **2b**, reaching 77% and 80% maximum conversions after 2 h, respectively. The biocatalysts Im-WY12-Ca and Im-WY12-Zn were further tested on 4-(4-benzylpiperazin-1-yl)butan-2-one **2d** as substrate. By using Im-WY12-Zn as biocatalyst in bioreduction of **2d**, higher conversion (10%) could be achieved after 24 h as with the non-immobilized cell form (3%; WY12 in Table 3), while Im-WY12-Ca was inactive (0% conversion). When *tert*-butyl 4-(3-oxobutyl)piperazine-1-carboxylate **2e** was tested as substrate, improved conversions as compared to WY12 (20%) could be observed (55% with Im-WY12-Ca and 83% with Im-WY12-Zn). The combined effect of decreased substrate concentration and increased catalyst/substrate ratio contributed markedly to the enhanced conversions in these bioreductions enabling a conversion range being useful for preparative applications.

### Recyclability of the immobilized biocatalysts

**Fig. 3:**
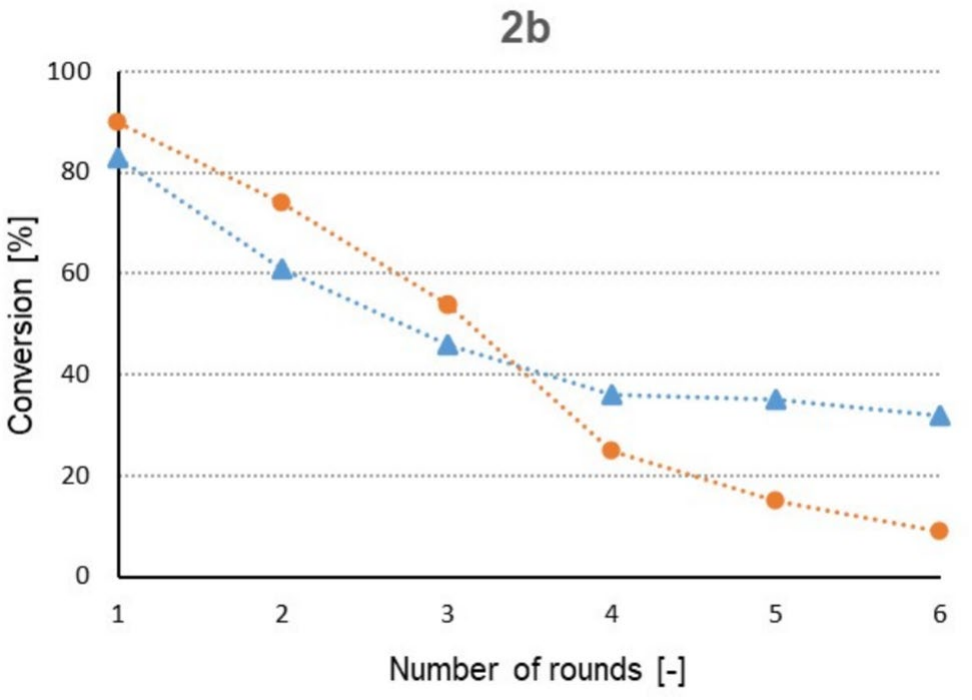
Recyclability of immobilized biocatalysts Im-WY12-Ca and Im-WY12-Zn in bioreduction reactions with 4-(indolin-1-yl)butan-2-one **2b** as the substrate;  — Im-WY12-Ca;  — Im-WY12-Zn; ee > 99% in all cases

Since with immobilized biocatalysts Im-WY12-Ca and Im-WY12-Zn the maximum conversion with substrate **2b** could be reached after only 2 h, the recyclability of the alginate beads was also tested in six subsequent reaction cycles with the same biocatalyst (Fig. 3).

After each cycle, the biocatalyst was separated from the reaction mixture and washed thoroughly (4 times) with buffer. Like in the previous bioreduction tests, the Zn^2+^-cross-linked Im-WY12-Zn resulted in higher conversion compared to Im-WY12-Ca in the first three cycles. On the other hand, the stability of the Ca^2+^-cross-linked Im-WY12-Ca seemed to be better, since after three cycles, the loss of activity was more pronounced for Im-WY12-Zn, while Im-WY12-Ca showed a less drastic decline (the conversion of **2b** in the 6th round dropped to 9% with Im-WY12-Zn but Im-WY12-Ca was still able to convert 32% of the substrate).

### Bioreductions of ketones 2a,b,d,e at preparative scale

Because out of the of five ketones (**2a-e**) investigated as substrates in KRED-containing yeast whole-cell biotransformations four (**2a,b,d,e**) could be converted to the enantiopure alcohols (*S*)-**1a,b,d,e** with good to excellent conversion, bioreductions were performed at preparative scale to isolate and characterize the enantiopure products (Table 4). The preparative scale bioreductions were performed with lyophilized cells of *Candida parapsilosis* (WY12: Entries 1–3 in Table 4) and with the Zn-alginate entrapped form (Im-WY12-Zn: Entries 4–6 in Table 4).

**Table 4:** Preparative bioreduction of ketones **2a-c; 2e** with the best performing yeast strain (*Candida parapsilosis* WY12) and its immobilized form (Im-WY12-Zn) at preparative scale

Entry	Alcohol	Biocatalyst	Conversion [%]	Yield [%]	Enantiomeric excess [%]	[α]_578_^1^	[α]_589_^2^
1	(*S*)-**1a**	WY12	84	14	> 99	26.0^3^	24.2
2	(*S*)-**1b**	WY12	85	35	> 99	n.d	n.d
3	(*S*)-**1c**	WY12	> 99	60	> 99	− 27.8	− 26.6^4^
4	(*S*)-**1a**	Im-WY12-Zn	71	49	> 99	26.9^3^	25.4
5	(*S*)-**1b**	Im-WY12-Zn	90	71	> 99	n.d	n.d
6	(*S*)-**1e**	Im-WY12-Zn	89	47	> 99	− 17.1	− 16.6

*n.d.* not determined.

^1^* T* = 25 °C, *λ* = 578 nm (*c* = 1, for Entries 2, 3, 4, 5; *c* = 0.5 for Entries 1, 6; CDCl_3_).

^2^* T* = 25 °C, *λ* = 589 nm (*c* = 1, for Entries 2, 3, 4, 5; *c* = 0.5 for Entries 1, 6; CDCl_3_).

^3^Lit. + 12.2 (ee = 67.3%; *T* = 20 °C, *λ* = 578 nm (c = 3, ethanol) (Silva et al. 2023)).

^4^Lit. − 6.1 (ee = 15.6%, *T* = 20 °C, *λ* = 589 nm (*c* = 0.49, CHCl_3_) (Ishizaka et al. 2023)); − 0.08 (ee = 3%, *T* = 25 °C, *λ* = 589 nm (*c* = 1.3, CHCl_3_) (Li et al. 2022)).

First, the scalability of *C. parapsilosis*-catalyzed reactions was tested with the original, lyophilized version of the biocatalyst. Although (*S*)−4-(4-phenylpiperazin-1-yl)butan-2-ol (*S*)-**1c** could be prepared this way with an acceptable yield (Entry 3), the extensive emulsion formation and foaming, thereby challenging phase separation during extraction led to low yields of (*S*)−4-(3,4-dihydroquinolin-1(2*H*)-yl)butan-2-ol (*S*)-**1a** (Entry 1) and (*S*)−4-(indolin-1-yl)butan-2-ol (*S*)-**1b** (Entry 2).

By using the Im-WY12-Zn form, the preparative scale production of (*S*)-*tert*-butyl 4-(3-hydroxybutyl)-piperazine-1-carboxylate (*S*)-**1e** also became possible (Entry 6, 47% yield). Application of immobilized *C. parapsilosis* simplified the work-up process due to the greatly decreased emulsion formation. Im-WY12-Zn was chosen for the preparative scale biotransformations, because this form exhibited the best performance with substrates **2a**, **2b**, and **2e** during small-scale reactions. Solving the downstream process problems enabled the synthesis of (*S*)-**1a** and (*S*)-**1b** with satisfactory preparative yields (Entries 4 and 5).

It is noteworthy that the preparative scale bioreductions exhibited comparable conversions to those observed in the small-scale screening reactions and all of the isolated (*S*)-alcohol products were virtually enantiopure (ee > 99%). The considerably lower yield of the pure alcohols (*S*)-**1a,b,c,e** achieved in the preparative scale reaction could be attributed to non-optimized chromatographic separation with considerable amounts of mixed fraction of the forming alcohols (*S*)-**1a,b,c,e** and the corresponding unconverted ketones **2a,b,c,e**. Unfortunately, in the case of the bioreductions of ketone **2b** to (*S*)−4-(indolin-1-yl)butan-2-ol (*S*)-**1b** (Entries 2 and 5 in Table 4), the unavoidable partial oxidation to its indole derivative (2–10%) was observed. Although, the decreased reaction time (2 h with Im-WY12-Zn instead of 24 h with WY12) suppressed the formation of the oxidative impurity, it was still detectable, hindering the determination of the optical rotation of (*S*)-**1b**.

### Assignment of absolute configuration to the produced alcohols (*S*)−1a-e

Although the majority of ADHs follow Prelog’s rule (Prelog 1964) in which the cofactor delivers its hydride from the *Re* face, and the bioreductions by KREDs following Prelog’s rule could be considered to produce (*S*)-alcohols (*S*)-**1a-e**, it was desirable to assign the absolute configurations of the present products (*S*)-**1a-e** not only on the basis of assumed analogy.

First, to assign absolute configuration of the alcohol products, the kinetic resolution of 4-(3,4-dihydroquinolin-1(2*H*)-yl)butan-2-ol ((**±**)-**1a**) was performed with an (*R*)-selective lipase enzyme, lipase PS from *Burkholderia cepacia* (Fig. S1). The kinetic resolution resulted in the preferred acetylation of (*R*)-**1a**, while (*S*)-**1a** remained almost intact. With the conversion of the reaction (*c*) and the enantiomeric excess of the product (*ee*_P_) determined, the high degree of enantiomer selectivity could be confirmed (*E*_c,eeP_ > 100). GC analysis on the enantiomer selective Hydrodex β−6TBDM column revealed that the less reactive enantiomer in the lipase-catalyzed kinetic resolution of 4-(3,4-dihydroquinolin-1(2*H*)-yl)butan-2-ol **1a** is the one which is forming in the majority of the yeast mediated bioreductions. This agrees with the expectations as lipases are usually (*R*)-selective (Kazlauskas et al. 1991), while the majority of ADHs follow Prelog’s rule (Prelog 1964) in which the cofactor delivers its hydride from the *Re* face, producing (*S*)-alcohol (*S*)-**1a**.

**Fig. 4:**
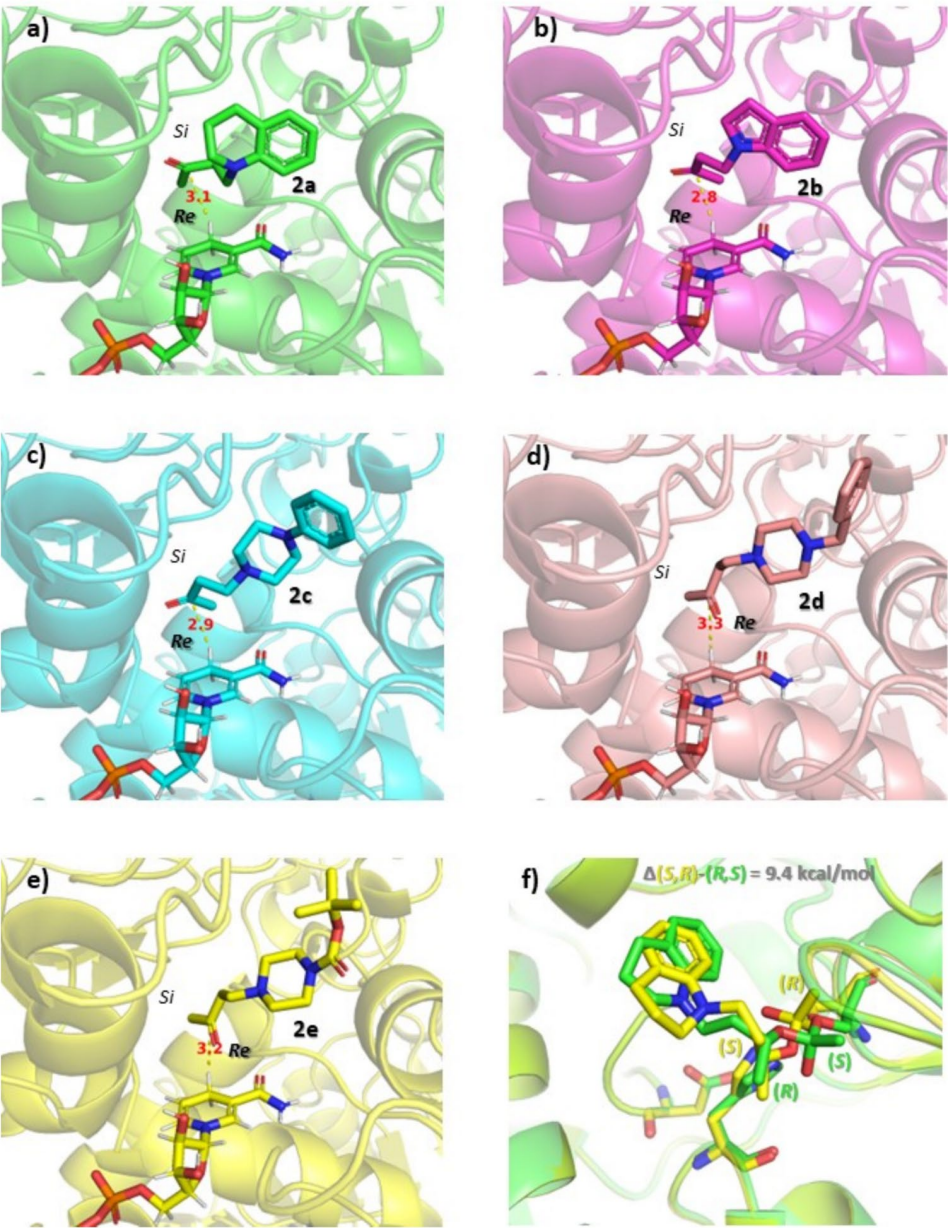
Absolute configuration assignment for the (*S*)-enantiomer of 4-(3,4-dihydroquinolin-1(2*H*)-yl)butan-2-ol (*S*)-**1a** performed by two independent calculation methods. Molecular docking (**a**–**e**) revealed the NADH at the *Re* face of the ketones **2a-e** in their reactive arrangements within the active site of the alcohol dehydrogenase of *Candida parapsilosis*. Comparative calculations (**f**) on the tetrahedral intermediates forming from the (*R*)- and (*S*)-enantiomers of **1a** in course of enzymatic acetylation (see Supplementary Information Sect. 1.1.4. and Figure S1) modeled into the experimental structure of lipase from *Burkholderia cepacia* showed (*R*)-enantiomer preference by 9.4 kcal/mol

Next, these expectations were further confirmed by computations (Fig. 4). The absolute configuration assignment for the (*S*)-enantiomer of 4-(3,4-dihydroquinolin-1(2*H*)-yl)butan-2-ol (*S*)-**1a** was performed by two independent methods: (i) by modeling the possible arrangement of all the five ketones **2a-e** within the active site of the alcohol dehydrogenase of *Candida parapsilosis* and (ii) by comparative calculations on the tetrahedral intermediates forming from the (*R*)- and (*S*)-enantiomer of **1a** in course of enzymatic acetylation modeled into the experimental structure of lipase from *Burkholderia cepacia*.

The isolation and application of an ADH—exhibiting broad substrate specificity and dependency on the cofactor NADH—from the yeast *Candida parapsilosis* (CpADH) were described (Peters et al. 1993). The high tolerance of CpADH for the isopropanol cosolvent allowed the enzyme to be applied at high concentration of the substrate by using isopropanol as the cosubstrate for NADH regeneration (Zelinski et al. 1999). Due to the synthetic usefulness of CpADH, the experimental structure of the carbonyl reductase from *C. parapsilosis* containing NADH cofactor (PDB ID: 4C4O; Man et al. 2014) was determined. This structure enabled us to perform docking studies and direct analysis of the stereochemical outcome of the allowed reactive arrangements of the *N*-(3-oxobutyl)heterocycle substrates **2a-e** in the active site of the active enzyme in *C. parapsilosis* WY12 biocatalysts. Analysis of 5 × 20 docking poses for each ketone **2a-e** revealed only non-reactive poses (> 4 Å distance between the carbonyl carbon and the *pro-R* H-atom transferring from the NADH) or reactive arrangements of the ketones facing the *Re* side of the carbonyl moiety toward the *pro-R* *H*-atom of the NADH within 4 Å (Fig. 4a–e). The hydride equivalent from the *Re* site results in formation of the (*S*)-alcohols proving the absolute configuration of all the prepared chiral alcohol products (*S*)-**1a-e**.

Related to a detailed study combining X-ray diffraction and QM/MM studies of the lipase from *Burkholderia cepacia* as biocatalyst in esterification of secondary alcohols, an experimental lipase structure containing covalently bound (2*S*)-(1-phenoxybut-2-yl)methylphosphonic acid inhibitor is available (PDB ID: 2NW6; Luić et al. 2008). After removing the ligand, the acetate enantiomers (*R*)-**3a** and (*S*)-**3a** could be docked into the active site of this experimental structure. Starting from the best scored reactive docking poses of acetate enantiomers (*R*)-**3a** and (*S*)-**3a** (with the carbonyl *C*-atom of the acetate in close vicinity of the *O*-atom of S87 (< 4.0 Å) and the carbonyl *O*-atom oriented toward the oxyanion hole), the four possible THIs representing high energy intermediates close to the rate determining transition states could be modeled. The relative energies of the four THI states showed 9.4 kcal/mol difference between the *R*_**1a**_,*S*_THI_ and *S*_**1a**_,*R*_THI_ THI states (Fig. 4f) favoring the faster conversion of (*R*)-**1a** as compared to (*S*)-**1a** to the corresponding acetate enantiomer.

## Discussion

The KRED-catalyzed bioreduction of prochiral *N*-(3-oxobutyl)heterocycles with flexible rings represents an unexplored area of research. Previous examples of prochiral ketones utilized as substrates in yeast mediated KRED-catalyzed bioreductions include aromatic ketones, e.g., acetophenone, phenylacetone or benzylacetone (Nagy-Győr et al. 2019). These aromatic ketones were converted to the corresponding (*S*)-alcohols using wild yeast strains *Candida norvegica*, *Pichia carsonii*, *Lodderomyces elongisporus*, and *Debaryomyces fabryi* with moderate to excellent conversions (49–97%) and high selectivities (> 95% ee) in all cases. In the present study, *C. parapsilosis* (WY12), *P. carsonii* (WY1), and *L. elongisporus* (WY2) exhibited similar, promising ketoreductase activities on the targeted *N*-(3-oxobutyl)heterocycles **2a-e**, while *C. norvegica* (WY4) proved to be only partially applicable for bioreductions. *D. fabryi* (WY11) did not demonstrate any activity with the five tested substrates.

According to the expectations based on the previous study (Nagy-Győr et al. 2019), the enantiomeric excess values were excellent. Relevant precedents in literature can be found for flexible carbocyclic analogues, for example, the bioreduction of 1-cyclohexylethanone was accomplished with several fungal species. Whole-cell biocatalyst *Lasiodiplodia theobromae* yielded the corresponding (*R*)-alcohol with 96% conversion and 35% enantiomeric excess (Barros-Filho et al. 2010), while a purified KRED from *Pichia guilliermondii* resulted in the same (*R*)-alcohol with 99.7% conversion and > 99% ee (Xu et al. 2013). Additional whole-cell biocatalysts have been employed in the bioreduction of 1-cyclohexylethanone, e.g., *Lentinus strigellus* yielded (*S*)−1-cyclohexylethanol with 99% conversion and 91% ee (Barros-Filho et al. 2009), while a fungus belonging to the *Trichothecium* genus resulted in the (*R*)-enantiomer with 85% conversion and 97.5% ee (Mandal et al. 2004). In another study, the heterocyclic ketone ethyl 1-benzyl-3-oxopiperidine-4-carboxylate was subjected to biocatalytic reduction with *Candida parapsilosis* and *Pichia methanolica*, resulting in the complete conversion of the substrate to the corresponding alcohol in both cases. Utilization of *C. parapsilosis* yielded a product with 97.4% diastereomeric and 99.8% enantiomeric excess, while *P. methanolica* resulted in the target alcohol with 99.5% diastereomeric and 98.2% enantiomeric excess (Guo et al. 2006).

Overall, the present study extends the applicability of native yeast whole cell biocatalysts to ketones involving flexible *N*-heterocycles. Moreover, whole-cell immobilization of the most useful biocatalyst *C. parapsilosis* (WY12) resulted in further enhancements. Previously, downstream processing of the bioreductions involving lyophilized yeasts presented a challenge due to emulsion formation and foaming during the extraction process. In a previous study, the problem of emulsion formation after bioreduction of 1-(arylsulfanyl)propan-2-ones with whole cells of *C. parapsilosis* was solved by extensive centrifugation (Sándor et al. 2024). The present study demonstrated that immobilization allows easier downstream processing of the reaction mixtures after bioreduction by filtration or decantation. This allowed the substrate concentration in the bioreductions to be decreased (from 50 to 10 mM) which increased the conversions of **2d** [from 3% (WY12) to 10% (Im-WY12-Zn)] and **2e** [from 20% (WY12) to 55% (Im-WY12-Ca) and 83% (Im-WY12-Zn)] most probably due to diminished substrate or product inhibition. Immobilization allowed also raising the biocatalyst-to-substrate ratio, leading to shorter reaction times diminishing the formation of possible byproducts, thereby resulting in higher purity alcohol products.

These effects contributed to achieving higher yields at preparative scale as compared to the process with non-immobilized cells [the yield of (*S*)-4-(3,4-dihydroquinolin-1(2*H*)-yl)butan-2-ol (*S*)-**1a** increased from 14 to 49%, while the yield of (*S*)−4-(indolin-1-yl)butan-2-ol (*S*)-**1b** increased from 35 to 71%]. By increasing the catalyst/substrate ratio and eliminating the possibility of substrate inhibition at higher concentrations, the preparative scale production of (*S*)-**1e** ((*S*)-*tert*-butyl 4-(3-hydroxybutyl)-piperazine-1-carboxylate) became feasible. This enantiopure chiral fragment is synthetically valuable, because after removal of the *tert*-butoxycarbonyl protecting group, the secondary amine function of the *N*-heterocycle can be further modified in a multitude of ways (e.g. by *N*-alkylation, *N*-acylation, etc.).

It is notable that molecular docking of the ketones **2a-e**, indicating their arrangement with the *Re* site facing toward the NADH cofactor, could confirm not only the absolute configuration of the forming (*S*)-alcohols (*S*)-**1a-e**, but also rationalized the differences between their reactivities. The distance of the *pro-R* *H*-atom and the carbonyl *C*-atom in the reactive arrangements of ketones **2a-c** docked into the 4C4O structure was shorter than 3.2 Å and the hydride transfer could happen from almost perpendicular to the trigonal carbonyl moiety. On the other hand, the arrangements of 4-(4-benzylpiperazin-1-yl)butan-2-one **2d** and *tert*-butyl 4-(3-oxobutyl)piperazine-1-carboxylate **2e** (larger than 3.2 Å distance and strong deviation from the ideal approaching angle) were in good agreement with the experiments indicating ketones **2d,e** as much worse substrates of WY12 (*Candida parapsilosis*) as compared to ketones **2a-c** (Table 3).

Finally, the recyclability of the immobilized biocatalysts Im-WY12-Ca and Im-WY12-Zn was also studied, with **2b**, 4-(indolin-1-yl)butan-2-one as the substrate. Im-WY12-Ca retained 39% of its original activity after six rounds, while Im-WY12-Zn exhibited a weaker performance, retaining only 10%, which is consistent with the data presented in relevant literature. Previously, immobilized *C. parapsilosis* in the form of calcium alginate beads has been utilized in the enantioselective oxidation of diols (Sivakumari and Chadha 2014). The authors examined the recyclability of the alginate beads and concluded that the first two cycles showed 100% activity, which subsequently decreased to 87% in the third cycle, and then declined markedly to 2% in the fourth cycle. In another study, the recyclability of immobilized* C. parapsilosis* (in the form of calcium alginate beads) was investigated in the bioreduction of acetyltrimethylsilane to (*R*)-1-trimethylsilylethanol (Zhang et al. 2012). After six cycles, the biocatalyst retained 50% of its original activity in aqueous monophasic system. Another fungus with ketoreductase activity, *Geotrichum candidum*, was immobilized in agar and calcium alginate (Liu et al. 2018). The recyclability of these immobilized biocatalysts were studied in the bioreduction of 1-(2-bromophenyl)ethanone. After six cycles, the agar-immobilized cells showed 15% of their original activity, while the calcium alginate-immobilized cells exhibited 3.7% of their original activity. In contrast, the non-immobilized *Geotrichum candidum* became almost completely inactive after three cycles.

Presumably, in the case of present study and previous studies as well, the numerous washing and shaking steps had a negative effect on the mechanical strength of the alginate beads, leading to possible leaching of the biocatalyst. This issue could be tackled by the means of cross-linking, which is among our future plans.

Our study indicates the possibility of widening the scope of substrates, including other (partially) saturated heterocycles (e.g., morpholine, thiomorpholine, pyrrolidine, piperidine, 4-methylpiperidine, 4-benzylpiperidine, ethyl piperidine-4-carboxylate, and tetrahydroisoquinoline) as well. Another possibility of synthetic improvements is to extend the types of biocatalysts from native yeasts to recombinant organisms overexpressing alcohol dehydrogenases (e.g., (*R*)-selective LkADH from *Lactobacillus kefir* or (*S*)-selective RaADH from *Rhodococcus aetherivorans*) as biocatalysts. Utilization of an (*R*)-selective alcohol dehydrogenase would enhance the scope of the enantiocomplementary bioreduction of the investigated prochiral ketones from the partial (*R*)-selectivity with our anti-Prelog yeast *R. glutinis* WY51 toward ketones **2a,b** to more perfect selectivity with a wider substrate range. These biocatalysts could also be immobilized in the form of alginate beads, preferably reinforced by cross-linking, enabling their recyclability or their utilization in flow reactors.

## Supplementary Information

Below is the link to the electronic supplementary material.

Supplementary file1 (PDF 3526 KB)

## Data Availability

The datasets generated and/or analyzed during the current study are available from the corresponding author on reasonable request.
