# Supplementary material for: Bioreduction of N-(3-oxobutyl)heterocycles with flexible ring by yeast whole-cell biocatalysts
Source: Appl Microbiol Biotechnol. 2025 Apr 30;109:108. doi: 10.1007/s00253-025-13486-2 (PMC12043778; doi:10.1007/s00253-025-13486-2)
Supplement: Supplementary file 1 [file 253_2025_13486_MOESM1_ESM.pdf]

Bioreduction of *N*-(3-oxobutyl)heterocycles with flexible ring by yeast whole-cell biocatalysts

Máté Gergő Honvári[1], Bence Attila Kucsinka[1], Levente András Mócza[1], Pál Csuka[1], Viktória Bódai[2,3], László Poppe[1,4]* and Gábor Hornyánszky[1]*

[1] Department of Organic Chemistry and Technology, Faculty of Chemical Technology and Biotechnology, Budapest University of Technology and Economics, H-1111 Budapest, Műegyetem rkp. 3, Hungary
[2] Fermentia Microbiological Ltd, H-1049 Budapest, Berlini út 47-49, Hungary
[3] Witaria Ltd, H-1087 Budapest, Luther utca 4–6, Hungary
[4] Biocatalysis and Biotransformation Research Centre, Faculty of Chemistry and Chemical Engineering, Babeş-Bolyai University of Cluj-Napoca, Arany János str. 11. RO-400028, Cluj-Napoca, Romania
* Correspondence: LP: poppe.laszlo@vbk.bme.hu; GH: hornyanszky.gabor@vbk.bme.hu

Table of contents

1. Materials and methods ***2***
1.1. Synthesis of the substrates, reference compounds and enantiopure alcohols ***2***
1.1.1. Synthesis of N-(3-oxobutyl)heterocycles 2a-e ***2***
1.1.2. Synthesis of racemic N-(3-hydroxybutyl)heterocycles (±)-1a-e ***2***
1.1.3. Bioreduction of ketones 2a-c, 2e to (S)-alcohols (S)-1a-c, (S)-1e at preparative scale ***3***
1.1.4. Absolute configuration of (S)-4-(3,4-dihydroquinolin-1(2H)-yl)butan-2-ol, (S)-1a ***4***
1.2. Gas chromatography ***5***
1.2.1. Temperature programs ***5***
1.2.2. Gas chromatograms of the starting materials and products ***5***
1.2.3. Gas chromatograms for determining the enantiomeric composition of the products ***8***
2. Spectra of the starting materials and products ***10***
3. References ***26***

1. Materials and methods

1.1. Synthesis of the substrates, reference compounds and enantiopure alcohols

*1.1.1. Synthesis of N-(3-oxobutyl)heterocycles **2a-e***

To a cooled (-5 °C) and stirred solution of the corresponding *N*-(3-oxobutyl)heterocycle (**4a-e**, 15 mmol) in dry Et_2O (20 mL) was added a solution of methyl vinyl ketone in dry Et_2O (**5**, 19.5 mmol, 1.3 eq., in 10 mL of Et_2O) dropwise (over ~30 min, below 5 °C). The resulting mixture was allowed to reach the room temperature and stirred for another 30 min. In the case of 4-(3,4-dihydroquinolin-1(2*H*)-yl)butan-2-one **2a** and 4-(indolin-1-yl)butan-2-one **2b** the solvent and the excess methyl vinyl ketone **5** was evaporated in vacuum to give the desired product. In the case of 4-(4-phenylpiperazin-1-yl)butan-2-one **2c**, 4-(4-benzylpiperazin-1-yl)butan-2-one **2d** and *tert*-butyl 4-(3-oxobutyl)piperazine-1-carboxylate **2e**, 2 eq. of 20% HCl in EtOAc was added dropwise to the reaction mixture to precipitate the dihydrochloride salt of these compounds. The precipitate was filtered, washed with ether (3×20 mL) and dried in vacuum to give the desired product.

4-(3,4-Dihydroquinolin-1(2*H*)-yl)butan-2-one, **2a**: Amber liquid (95% yield); Rf = 0.55 (hexane/EtOAc 10:4); ^{1}H NMR ($CDCl_3$, 500 MHz) δ 7.07-7.04 (t, *J* = 8.1 Hz, 1H, Ar-H), 6.97-6.95 (d, *J* = 7.3 Hz, 1H, Ar-H), 6.61-6.58 (t, *J* = 7.3 Hz, 1H, Ar-H), 6.56-6.54 (d, *J* = 8.1 Hz, 1H, Ar-H), 3.59-3.56 (t, *J* = 7.0 Hz, 2H, CH_2), 3.28-3.26 (t, *J* = 6.2 Hz, 2H, CH_2), 2.76-2.73 (m, 4H, 2×CH_2), 2.18 (s, 3H, CH_3), 1.97-1.92 (p, *J* = 6.2 Hz, 6.0 Hz, 2H, CH_2); ^{13}C NMR ($CDCl_3$, 126 MHz) δ 208.3 (C=O), 144.7 (Ar-C), 129.4 (Ar-C), 127.3 (Ar-C), 122.9 (Ar-C), 116.1 (Ar-C), 110.5 (Ar-C), 49.7 (CH_2), 46.0 (CH_2), 40.1 (CH_2), 30.8 (CH_3), 28.1 (CH_2), 22.4 (CH_2); IR (KBr): ν (cm^{-1}) 1709 (νC=O), 1600 (aromatic νC=C), 1498 (aromatic νC=C). Spectral data agreed with the published IR (Silva et al. 2023) as well as ^{1}H-NMR and ^{13}C-NMR (Ouyang et al. 2022) data.

4-(Indolin-1-yl)butan-2-one, **2b**: Purple liquid (98% yield); Rf = 0.46 (hexane/EtOAc 10:4); ^{1}H NMR ($CDCl_3$, 500 MHz) δ 7.09-7.06 (m, 2H, 2×Ar-H), 6.68-6.66 (t, *J* = 6.8 Hz, 1H, Ar-H), 6.51-6.50 (d, *J* = 7.7 Hz, 1H, Ar-H), 3.41-3.38 (t, *J* = 7.0 Hz, 2H, CH_2), 3.35-3.32 (t, *J* = 8.3 Hz, 2H, CH_2), 2.97-2.93 (t, *J* = 8.3 Hz, 2H, CH_2), 2.75-2.72 (t, *J* = 7.0 Hz, 2H, CH_2), 2.20 (s, 3H, CH_3); ^{13}C NMR ($CDCl_3$, 126 MHz) δ 207.8 (C=O), 152.0 (Ar-C), 130.2 (Ar-C), 127.5 (Ar-C), 124.6 (Ar-C), 118.0 (Ar-C), 107.1 (Ar-C), 53.4 (CH_2), 44.0 (CH_2), 41.2 (CH_2), 30.5 (CH_3), 28.7 (CH_2); IR (KBr): ν (cm^{-1}) 1710 (νC=O), 1605 (aromatic νC=C), 1487 (aromatic νC=C). Spectral data agreed with the published IR, ^{1}H-NMR and ^{13}C-NMR (Kilic et al. 2012) data.

4-(4-Phenylpiperazin-1-yl)butan-2-one dihydrochloride, **2c**·2 HCl: White crystals (90% yield); Rf = 0.80 (dichloromethane/MeOH/NH_4OH 10:1:0.2); melting point: 160 °C (decomposes); ^{1}H NMR (D_2O, 500 MHz) δ 7.47-7.43 (t, *J* = 8.0 Hz, 2H, 2×Ar-H), 7.23-7.22 (d, *J* = 7.8 Hz, 2H, 2×Ar-H), 7.20-7.17 (t, *J* = 7.4 Hz, 1H, Ar-H), 3.57 (s, broad, 8H, 4×CH_2), 3.51-3.48 (t, *J* = 6.7 Hz, 2H, CH_2), 3.18-3.15 (t, *J* = 6.7 Hz, 2H, CH_2), 2.27 (s, 3H, CH_3); ^{13}C NMR (D_2O, 126 MHz) δ 209.8 (C=O), 147.0 (Ar-C), 129.9 (2×Ar-C), 124.2 (Ar-C), 118.2 (2×Ar-C), 51.3 (CH_2), 51.2 (2×CH_2), 47.8 (2×CH_2), 36.9 (CH_2), 29.3 (CH_3); IR (KBr): ν (cm^{-1}) 1713 (νC=O), 1489 (aromatic νC=C).

4-(4-Benzylpiperazin-1-yl)butan-2-one dihydrochloride, **2d**·2 HCl: White crystals (53% yield); Rf = 0.69 (dichloromethane/MeOH/NH_4OH 10:1:0.2); melting point: 139 °C; ^{1}H NMR (D_2O, 500 MHz) δ 7.50 (m, 3H, 3×Ar-H), 7.47 (m, 2H, 2×Ar-H), 4.14 (s, 2H, CH_2), 3.21 (s, broad, 8H, 4×CH_2), 3.18-3.15 (t, *J* = 6.9 Hz, 2H, CH_2), 3.01-2.98 (t, *J* = 6.9 Hz, 2H, CH_2), 2.24 (s, 3H, CH_3); ^{13}C NMR (D_2O, 126 MHz) δ 211.3 (C=O), 136.4 (Ar-C), 130.8 (2×Ar-C), 129.6 (Ar-C), 129.1 (2×Ar-C), 60.6 (CH_2), 51.0 (CH_2), 49.9 (2×CH_2), 49.5 (2×CH_2), 38.0 (CH_2), 29.4 (CH_3); IR (KBr):ν (cm^{-1}) 1710 (νC=O), 1478 (aromatic νC=C). Spectral data agreed with the published IR (Roth and Mühlenbruch 1970) data.

tert-Butyl 4-(3-oxobutyl)piperazine-1-carboxylate dihydro-chloride, **2e**·2 HCl: White crystals (52% yield); Rf = 0.64 (dichloromethane/ MeOH/NH_4OH 10:1:0.2); melting point: 180 °C (decomposes); ^{1}H NMR (D_2O, 500 MHz) δ 4.46-2.81 (s, broad, 8H, 4×CH_2), 3.46-3.43 (t, *J* = 6.7 Hz, 2H, CH_2), 3.17-3.14 (t, *J* = 6.7 Hz, 2H, CH_2), 2.27 (s, 3H, CH_3), 1.47 (s, 9H, 3×CH_3); ^{13}C NMR (D_2O, 126 MHz) δ 209.8 (C=O), 155.5 (N-COO), 82.7 (C), 51.7 (2×CH_2), 51.3 (CH_2), 43.0 (2×CH_2), 36.8 (CH_2), 29.2 (CH_3), 27.5 (3×CH_3); IR (KBr): ν (cm^{-1}) 1715 (νC=O), 1693 (νC=O). Spectral data agreed with the published ^{1}H-NMR (Desantis et al. 2022) data.

*1.1.2. Synthesis of racemic N-(3-hydroxybutyl)heterocycles (±)-**1a-e***

To a cooled (-5 °C) and stirred solution of the corresponding ketone (**2a-e**, 2 mmol) in MeOH (5 mL) was added sodium borohydride (3 mmol, 1.5 eq. for **2a,b**; 7 mmol, 3.5 eq. for **2c-e**·2 HCl) portionwise (over ~30 min). The resulting mixture was allowed to reach room temperature, then was stirred for another 30 min. The reaction mixture was evaporated in vacuum, diluted with dichloromethane (30 mL) and washed with water (3×30 mL). The organic phase was dried over sodium sulfate and concentrated in vacuum to give the desired product.

4-(3,4-Dihydroquinolin-1(2*H*)-yl)butan-2-ol, (±)-**1a**: Amber liquid (74% yield); Rf = 0.47 (hexane/EtOAc 10:4); ^{1}H NMR ($CDCl_3$, 500 MHz) δ 7.07-7.05 (t, *J* = 7.7 Hz, 1H, Ar-H), 6.96-6.95 (d, *J* = 7.3 Hz, 1H, Ar-H), 6.68-6.66 (d, *J* = 8.2 Hz, 1H, Ar-H), 6.61-6.58 (t, *J* = 7.3 Hz, 1H, Ar-H), 3.94 (m, 1H, CH), 3.40 (m, 2H, CH_2), 3.27 (m, 2H, CH_2), 2.77-2.75 (t, *J* = 6.4 Hz, 2H, CH_2),

2.16 (s, broad, 1H, OH), 1.98-1.93 (p, J = 6.0 Hz, 2H, CH_2), 1.73 (m, 2H, CH_2), 1.25-1.24 (d, J = 6.2 Hz, 3H, CH_3); ^{13}C NMR ($CDCl_3$, 126 MHz) δ 145.6 (Ar-C), 129.4 (Ar-C), 127.2 (Ar-C), 123.1 (Ar-C), 116.3 (Ar-C), 111.5 (Ar-C), 67.1 (CH), 49.6 (CH_2), 49.3 (CH_2), 35.5 (CH_2), 28.2 (CH_2), 24.2 (CH_3), 22.3 (CH_2); IR (KBr): ν (cm^{-1}) 1600 (aromatic νC=C), 1502 (aromatic νC=C), 1120 (νC–O). Spectral data agreed with the published IR, ^{1}H-NMR and ^{13}C-NMR (Deb at al. 2013) data.

4-(Indolin-1-yl)butan-2-ol, (±)-**1b**: Purple liquid (73% yield); Rf = 0.35 (hexane/EtOAc 10:4); ^{1}H NMR ($CDCl_3$, 500 MHz) δ 7.12-7.09 (m, 2H, 2×Ar-H), 6.76-6.73 (t, J = 7.3 Hz, 1H, Ar-H), 6.65-6.63 (d, J = 7.8 Hz, 1H, Ar-H), 4.02 (m, 1H, CH), 3.51-3.46 (m, 1H, CH_2), 3.32 (m, 1H, CH_2), 3.26 (m, 1H, CH_2), 3.17 (m, 1H, CH_2), 2.98-2.95 (m, 3H, CH_2+OH), 1.77 (m, 2H, CH_2), 1.26-1.25 (d, J = 6.2 Hz, 3H, CH_3); ^{13}C NMR ($CDCl_3$, 126 MHz) δ 152.2 (Ar-C), 130.7 (Ar-C), 127.5 (Ar-C), 124.7 (Ar-C), 119.1 (Ar-C), 108.6 (Ar-C), 68.1 (CH), 54.1 (CH_2), 48.9 (CH_2), 35.9 (CH_2), 28.7 (CH_2), 23.8 (CH_3); IR (KBr): ν (cm^{-1}) 1606 (aromatic νC=C), 1487 (aromatic νC=C), 1129 (νC–O). Spectral data agreed with the published ^{1}H-NMR and ^{13}C-NMR (Nakamura et al. 2015) data.

4-(4-Phenylpiperazin-1-yl)butan-2-ol, (±)-**1c**: White solid (71% yield); Rf = 0.61 (dichloromethane/MeOH/NH_4OH 10:1:0.2); melting point: 114 °C; ^{1}H NMR ($CDCl_3$, 500 MHz) δ 7.28-7.24 (t, 2H, J = 8.0 Hz, 2×Ar-H), 6.92-6.91 (d, J = 7.7 Hz, 2H, 2×Ar-H), 6.88-6.85 (t, J = 7.3 Hz, 1H, Ar-H), 4.75 (s, broad, 1H, OH), 3.99 (m, 1H, CH), 3.21 (m, 4H, 2×CH_2), 2.83 (m, 2H, CH_2), 2.76-2.70 (m, 1H, CH_2), 2.66-2.61 (m, 1H, CH_2), 2.57 (m, 2H, CH_2), 1.69 (m, 1H, CH_2), 1,57-1.52 (m, 1H, CH_2), 1.20-1.18 (d, J = 6.2 Hz, 3H, CH_3); ^{13}C NMR ($CDCl_3$, 126 MHz) δ 151.2 (Ar-C), 129.3 (2×Ar-C), 120.1 (Ar-C), 116.3 (2×Ar-C), 69.9 (CH), 57.9 (CH_2), 53.4 (2×CH_2), 49.3 (2×CH_2), 33.4 (CH_2), 23.5 (CH_3); IR (KBr): ν (cm^{-1}) 1598 (aromatic νC=C), 1498 (aromatic νC=C), 1110 (νC–O). Spectral data agreed with the published ^{1}H-NMR and ^{13}C-NMR (Zhu et al. 2024) data.

4-(4-Benzylpiperazin-1-yl)butan-2-ol, (±)-**1d**: White solid (85% yield); Rf = 0.52 (dichloromethane/MeOH/NH_4OH 10:1:0.2); melting point: 99 °C; ^{1}H NMR ($CDCl_3$, 500 MHz) δ 7.29 (m, 4H, 4×Ar-H), 7.25 (m, 1H, Ar-H), 3.94 (m, 1H, CH), 3.49 (d, J = 3.3 Hz, 2H, CH_2), 2.68-2.48 (m, broad, 10H, 5×CH_2), 1.62 (m, 1H, CH_2), 1.50-1.45 (m, 1H, CH_2), 1.16-1.15 (d, J = 6.2 Hz, 3H, CH_3); ^{13}C NMR ($CDCl_3$, 126 MHz) δ 137.9 (Ar-C), 129.2 (2×Ar-C), 128.2 (2×Ar-C), 127.1 (Ar-C), 69.7 (CH), 62.9 (CH_2), 57.7 (CH_2), 52.9 (2×CH_2), 33.2 (CH_2), 23.4 (CH_3); IR (KBr): ν (cm^{-1}) 1451 (aromatic νC=C), 1106 (νC–O).

tert-Butyl 4-(3-hydroxybutyl)piperazine-1-carboxylate, (±)-**1e**: Colorless liquid (92% yield); Rf = 0.46 (dichloromethane/MeOH/NH_4OH 10:1:0.2); ^{1}H NMR ($CDCl_3$, 500 MHz) δ 3.95 (m, 1H, CH), 3.43 (m, 4H, 2×CH_2), 2.69-2.55 (m, 4H, 2×CH_2), 2.34 (m, 2H, CH_2), 1.64 (m, 1H, CH_2), 1.53-1.48 (m, 1H, CH_2), 1.44 (s, 9H, 3×CH_3), 1.17-1.15 (d, J = 6.2 Hz, 3H, CH_3); ^{13}C NMR ($CDCl_3$, 126 MHz) δ 154.7 (N-COO), 80.0 (C), 69.8 (CH), 57.9 (CH_2), 53.2 (2×CH_2), 43.6 (2×CH_2), 33.3 (CH_2), 28.5 (3×CH_3), 23.5 (CH_3); IR (KBr): ν (cm^{-1}) 1692 (νC=O), 1102 (νC–O).

*1.1.3. Bioreduction of ketones **2a-c**, **2e** to (S)-alcohols (S)-**1a-c**, (S)-**1e** at preparative scale*

(*S*)-4-(3,4-Dihydroquinolin-1(2*H*)-yl)butan-2-ol, (*S*)-**1a**: Amber liquid (yield: 14% (WY12); 49% (Im-WY12-Zn)); Rf = 0.47 (hexane/EtOAc 10:4); ^{1}H NMR ($CDCl_3$, 500 MHz) δ 7.07-7.05 (t, J = 7.7 Hz, 1H, Ar-H), 6.96-6.95 (d, J = 7.3 Hz, 1H, Ar-H), 6.68-6.66 (d, J = 8.2 Hz, 1H, Ar-H), 6.61-6.58 (t, J = 7.3 Hz, 1H, Ar-H), 3.94 (m, 1H, CH), 3.40 (m, 2H, CH_2), 3.27 (m, 2H, CH_2), 2.77-2.75 (t, J = 6.4 Hz, 2H, CH_2), 2.16 (s, broad, 1H, OH), 1.98-1.93 (p, J = 6.0 Hz, 2H, CH_2), 1.73 (m, 2H, CH_2), 1.25-1.24 (d, J = 6.2 Hz, 3H, CH_3); ^{13}C NMR ($CDCl_3$, 126 MHz) δ 145.6 (Ar-C), 129.4 (Ar-C), 127.2 (Ar-C), 123.1 (Ar-C), 116.3 (Ar-C), 111.5 (Ar-C), 67.1 (CH), 49.6 (CH_2), 49.3 (CH_2), 35.5 (CH_2), 28.2 (CH_2), 24.2 (CH_3), 22.3 (CH_2). Spectral data agreed with the published ^{1}H-NMR and ^{13}C-NMR (Silva et al. 2023) data.

(*S*)-4-(Indolin-1-yl)butan-2-ol, (*S*)-**1b**: Purple liquid (yield: 35% (WY12); 71% (Im-WY12-Zn)); Rf = 0.35 (hexane/EtOAc 10:4); ^{1}H NMR ($CDCl_3$, 500 MHz) δ 7.12-7.09 (m, 2H, 2×Ar-H), 6.76-6.73 (t, J = 7.3 Hz, 1H, Ar-H), 6.65-6.63 (d, J = 7.8 Hz, 1H, Ar-H), 4.02 (m, 1H, CH), 3.51-3.46 (m, 1H, CH_2), 3.32 (m, 1H, CH_2), 3.26 (m, 1H, CH_2), 3.17 (m, 1H, CH_2), 2.98-2.95 (m, 3H, CH_2+OH), 1.77 (m, 2H, CH_2), 1.26-1.25 (d, J = 6.2 Hz, 3H, CH_3); ^{13}C NMR ($CDCl_3$, 126 MHz) δ 152.2 (Ar-C), 130.7 (Ar-C), 127.5 (Ar-C), 124.7 (Ar-C), 119.1 (Ar-C), 108.6 (Ar-C), 68.1 (CH), 54.1 (CH_2), 48.9 (CH_2), 35.9 (CH_2), 28.7 (CH_2), 23.8 (CH_3).

(*S*)-4-(4-Phenylpiperazin-1-yl)butan-2-ol, (*S*)-**1c**: White solid (60% yield); Rf = 0.61 (dichloromethane/MeOH/NH_4OH 10:1:0.2); ^{1}H NMR ($CDCl_3$, 500 MHz) δ 7.28-7.24 (t, 2H, J = 8.0 Hz, 2×Ar-H), 6.92-6.91 (d, J = 7.7 Hz, 2H, 2×Ar-H), 6.88-6.85 (t, J = 7.3 Hz, 1H, Ar-H), 4.75 (s, broad, 1H, OH), 3.99 (m, 1H, CH), 3.21 (m, 4H, 2×CH_2), 2.83 (m, 2H, CH_2), 2.76-2.70 (m, 1H, CH_2), 2.66-2.61 (m, 1H, CH_2), 2.57 (m, 2H, CH_2), 1.69 (m, 1H, CH_2), 1,57-1.52 (m, 1H, CH_2), 1.20-1.18 (d, J = 6.2 Hz, 3H, CH_3); ^{13}C NMR ($CDCl_3$, 126 MHz) δ 151.2 (Ar-C), 129.3 (2×Ar-C), 120.1 (Ar-C), 116.3 (2×Ar-C), 69.9 (CH), 57.9 (CH_2), 53.4 (2×CH_2), 49.3 (2×CH_2), 33.4 (CH_2), 23.5 (CH_3). Spectral data agreed with the published ^{1}H-NMR and ^{13}C-NMR (Xu et al. 2020) data.

(*S*)-*tert*-Butyl 4-(3-hydroxybutyl)piperazine-1-carboxylate, (*S*)-**1e**: Colorless liquid (47% yield); Rf = 0.46 (dichloromethane/MeOH/NH_4OH 10:1:0.2); ^{1}H NMR ($CDCl_3$, 500 MHz) δ 3.95 (m, 1H, CH), 3.43 (m, 4H, 2×CH_2), 2.69-2.55 (m, 4H, 2×CH_2), 2.34 (m, 2H, CH_2), 1.64 (m, 1H, CH_2), 1.53-1.48 (m, 1H, CH_2), 1.44 (s, 9H, 3×CH_3), 1.17-1.15 (d, J = 6.2 Hz, 3H, CH_3); ^{13}C NMR ($CDCl_3$, 126 MHz) δ 154.7 (N-COO), 80.0 (C), 69.8 (CH), 57.9 (CH_2), 53.2 (2×CH_2), 43.6 (2×CH_2), 33.3 (CH_2), 28.5 (3×CH_3), 23.5 (CH_3).

*1.1.4. Absolute configuration of (S)-4-(3,4-dihydroquinolin-1(2H)-yl)butan-2-ol, (S)-**1a***

Kinetic resolution with lipase from Burkholderia cepacia (Fig. S1): A solution of (±)-**1a**, 4-(3,4-dihydroquinolin-1(2*H*)-yl)butan-2-ol (40 mg, 0.195 mmol) was dissolved in a mixture of 8 mL phosphate buffer (pH = 7.0, 64 mM) and 2 ml DMSO. Amano Lipase PS (lipase from *Burkholderia cepacia*, 200 mg) and vinyl acetate (1500 µL, 83.3 eq.) was added to the solution and the reaction was performed at 50 °C for 4 h, with samples (100 µL) taken at regular intervals. The sample preparation method was the same as above. After determining the molar response factor of acetylated alcohol **3a** to alcohol **1a** (*f*= 1.11), GC analysis on Hydrodex β-6TBDM column revealed that the residual (less reactive) enantiomer of 4-(3,4-dihydroquinolin-1(2*H*)-yl)butan-2-ol **1a** is the one which is forming in the bioreduction.

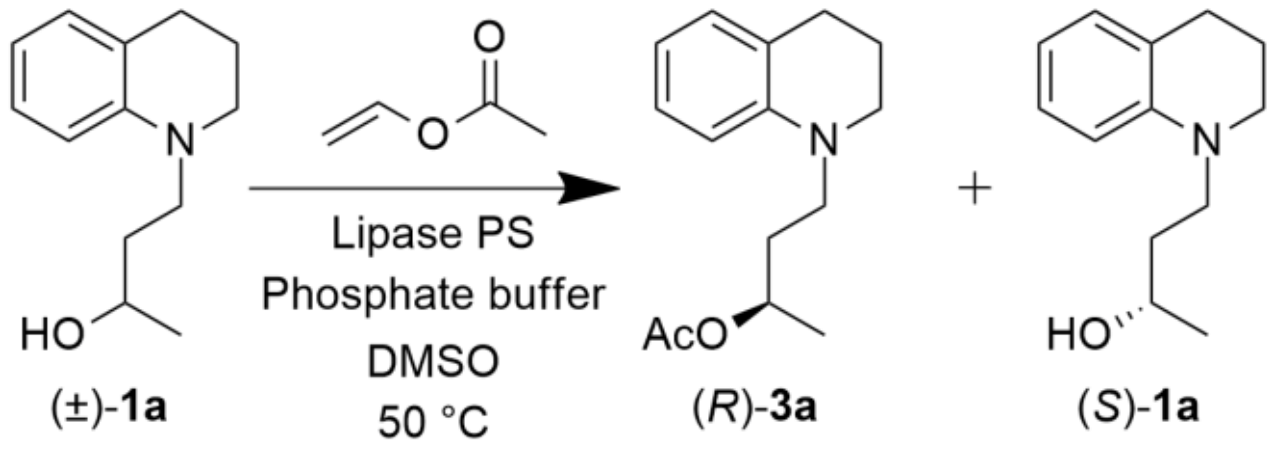

Fig. S1 Kinetic resolution of racemic (±)-4-(3,4-dihydroquinolin-1(2*H*)-yl)butan-2-ol (±)-**1a** with lipase PS from *Burkholderia cepacia*

Modeling the tetrahedral intermediates from the enantiomers of 4-(3,4-dihydroquinolin-1(2H)-yl)butan-2-ol within the active site of lipase from Burkholderia cepacia in the acetylation: The experimental structure of the lipase from *Burkholderia cepacia* (PDB ID: 2NW6 (Luić et al. 2008)) freed from covalently bound (2*S*)-(1-phenoxybut-2-yl)methylphosphonic acid inhibitor was applied for the calculations.

The structures of the two enantiomers of 4-(3,4-dihydroquinolin-1(2*H*)-yl)butan-2-yl acetate (*R*)-**3a** and (*S*)-**3a** with correct stereochemistry were generated in HyperChem (HyperChem 2009). The docking was performed by using Autodock VINA (Trott & Olson 2010; Eberhardt et al. 2021) implemented in PyMOL (DeLano 2002) by the DockingPie plugin (Rosignoli & Paiardini 2022).

After importing receptor and ligand structures from PyMOL into the DockingPie, the grid (centered at the *O*-atom of S87) settings were set in a way to incorporate all parts of the lipase structure. Five docking runs were performed resulting in 25 poses for each run. Those docking poses with the best docking scores (*ds*) were considered as the best reactive arrangement in which the carbonyl *C*-atom of the acetate was in close vicinity of the *O*-atom of S87 (<4.0 Å) and the carbonyl *O*-atom oriented towards the oxyanion hole (Run 5, Pose 1, *ds*= -6.838 kcal/mol for (*R*)-**3a**; Run 3, Pose 6, *ds*= -6.157 kcal/mol for (*S*)-**3a**). The best poses for (*R*)-**3a** and (*S*)-**3a** were energy minimized in the ligand containing lipase structure by Amber99 force-field (Showalter & Brüschweiler 2007) in HyperChem (HyperChem 2009; the S87 *O*-atom was deprotonated, and the charge was set to -0.65; the residues around a 15 Å sphere centered at the Cβ carbon of S87 were set as active while the other residues of the system were kept frozen, Polak-Ribier conjugate gradient, until conversion limit of 0.1 kcal/mol, typically within 600 cycles).
The tetrahedral intermediates representing the best transition states of the reaction pathway from the alcohol enantiomers (*R*)-**1a** and (*S*)-**1a** to the corresponding acetate enantiomers (*R*)-**3a** and (*S*)-**3a** were generated from the energy minimized structures within the lipase using HyperChem (HyperChem 2009; a bond between the carbonyl *C*-atom of the acetyl moiety of (*R*)-**3a** or (*S*)-**3a** and the S87 *O*-atom was created, the bond order of the acetyl C=O was decreased to 1, the charge of its *O*-atom was set to -0.65, the charge of the S87 *O*-atom was removed, residues around the 15 Å sphere centered at the Cβ carbon of S87 were set as active while the other residues of the system were kept frozen, Polak-Ribier conjugate gradient optimization with Amber99 until conversion limit of 0.1 kcal/mol, typically within 600 cycles) resulting in energy minimized structures of the possible THI-states for alcohol ((*R*)-**1a** or (*S*)-**1a**) to acetate ((*R*)-**3a** or (*S*)-**3a**) conversion. Several consecutive cycles of molecular dynamics (0.3 ps, until ~260 K) / energy minimization resulting in structures of decreasing energy until the energy decrease ceased (usually 3 to 7 cycles), resulted in the relative energies of the four THI states (0.0 kcal/mol for $R_{\mathbf{1a}},S_{\mathrm{THI}}$; 9.4 kcal/mol for $S_{\mathbf{1a}},R_{\mathrm{THI}}$; 16.4 kcal/mol for $S_{\mathbf{1a}},S_{\mathrm{THI}}$; 26.1 kcal/mol for $R_{\mathbf{1a}},R_{\mathrm{THI}}$).

1.2. Gas chromatography

1.2.1. Temperature programs

Table S1 GC methods and retention times of **2a-e**, (±) **1a-e** and (*S*)- and (*R*)-**3a-e** to determine conversion and enantiomeric composition

**Substrate**	**Method**	**Retention time (min)**			
		**2**	(±)-**1**	(*S*)-**3**	(*R*)-**3**
**2a**	150 °C; 5 °C min^{-1} to 210 °C[1]	10.08	11.27	11.71	11.83
**2b**	150 °C; 5 °C min^{-1} to 210 °C[1]	8.08	8.91	9.37	9.52
**2c**	150 °C; 5 °C min^{-1} to 210 °C[2]	7.88	7.73		
**2c**	185 °C; 0.5 °C min^{-1} to 195 °C; 5 min[1]			21.15	21.55
**2d**	150 °C; 5 °C min^{-1} to 210 °C[2]	7.38	7.28		
**2d**	185 °C; 0.5 °C min^{-1} to 195 °C[1]			16.68	17.01
**2e**	150 °C; 5 °C min^{-1} to 210 °C[1]	10.40	10.57	11.45	11.59

[1] Agilent 4890 GC equipped with FID detector and Hydrodex β-6TBDM column [25 m × 0.25 mm × 0.25 µm film with heptakis-(2,3-di-*O*-methyl-6-*O*-t-butyldimethylsilyl)-β-cyclodextrin; Macherey & Nagel (Düren, Germany); H_2 carrier gas (injector: 250 °C, detector: 250 °C, head pressure: 12 psi, split ratio: 50:1)].

[2] Agilent 5890 GC equipped with FID detector and HP-5 column [30 m × 0.25 mm × 0.25 µm film with (5%-phenyl)-methylpolysiloxane; Agilent Technologies (Santa Clara, California, U.S.); H_2 carrier gas (injector: 250 °C, detector: 250 °C, head pressure: 15 psi, split ratio: 50:1)].

1.2.2. Gas chromatograms of the starting materials and products

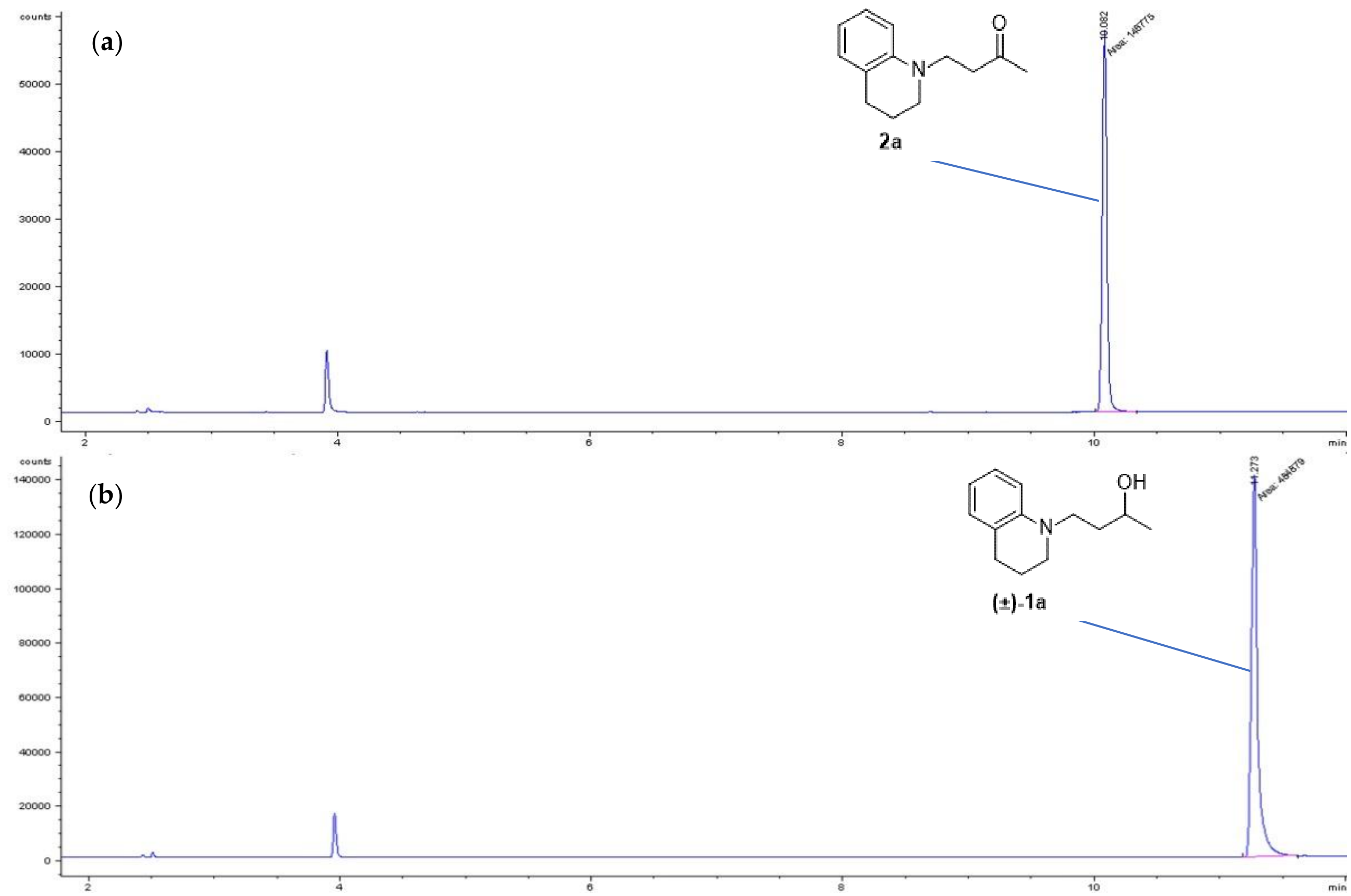

Fig. S2 Chromatogram of (**a**) 4-(3,4-dihydroquinolin-1(2*H*)-yl)butan-2-one **2a** and (**b**) 4-(3,4-dihydroquinolin-1(2*H*)-yl)butan-2-ol (±)-**1a**

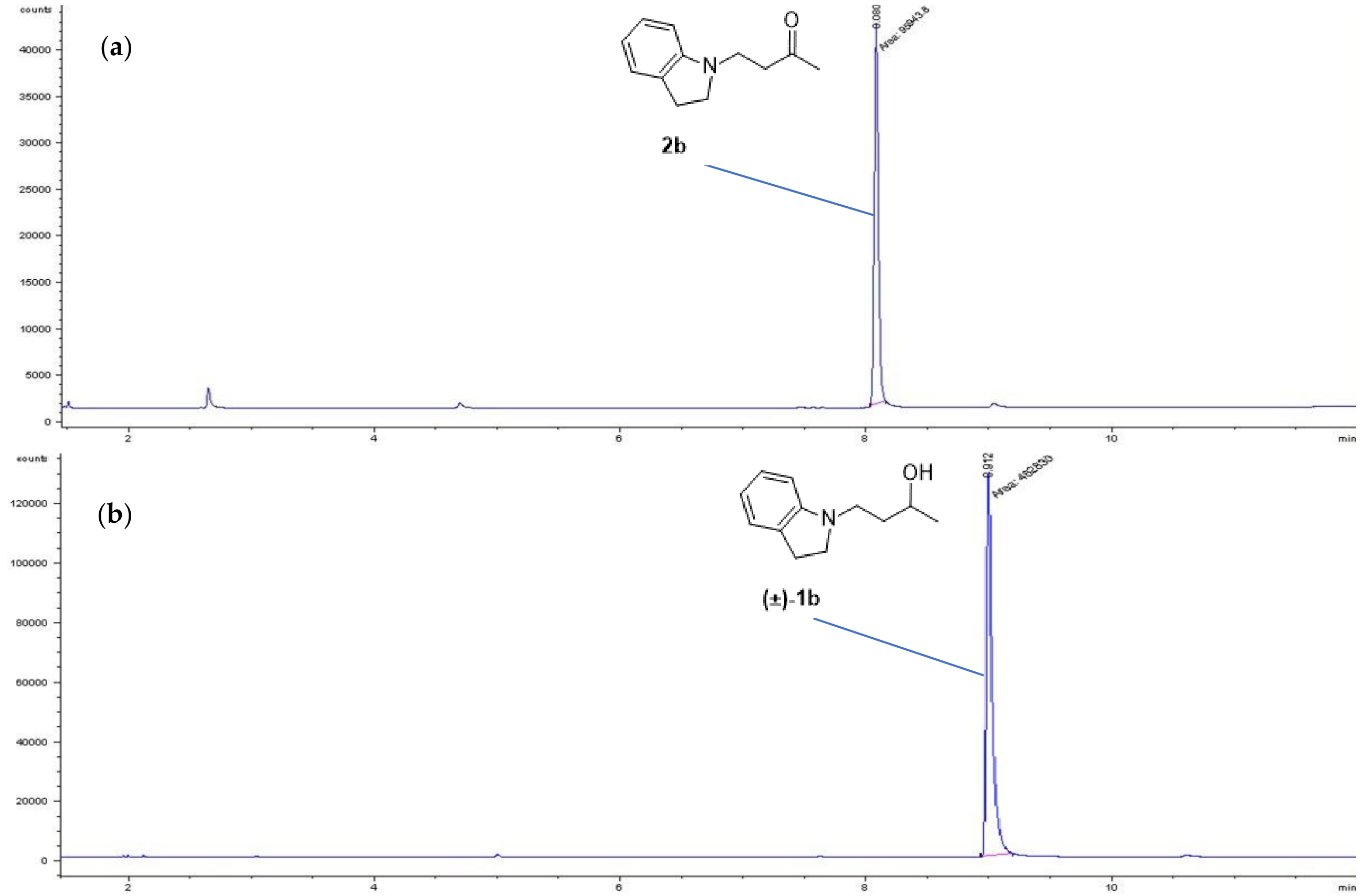

Fig. S3 Chromatogram of (**a**) 4-(indolin-1-yl)butan-2-one **2b** and (**b**) 4-(indolin-1-yl)butan-2-ol (±)-**1b**

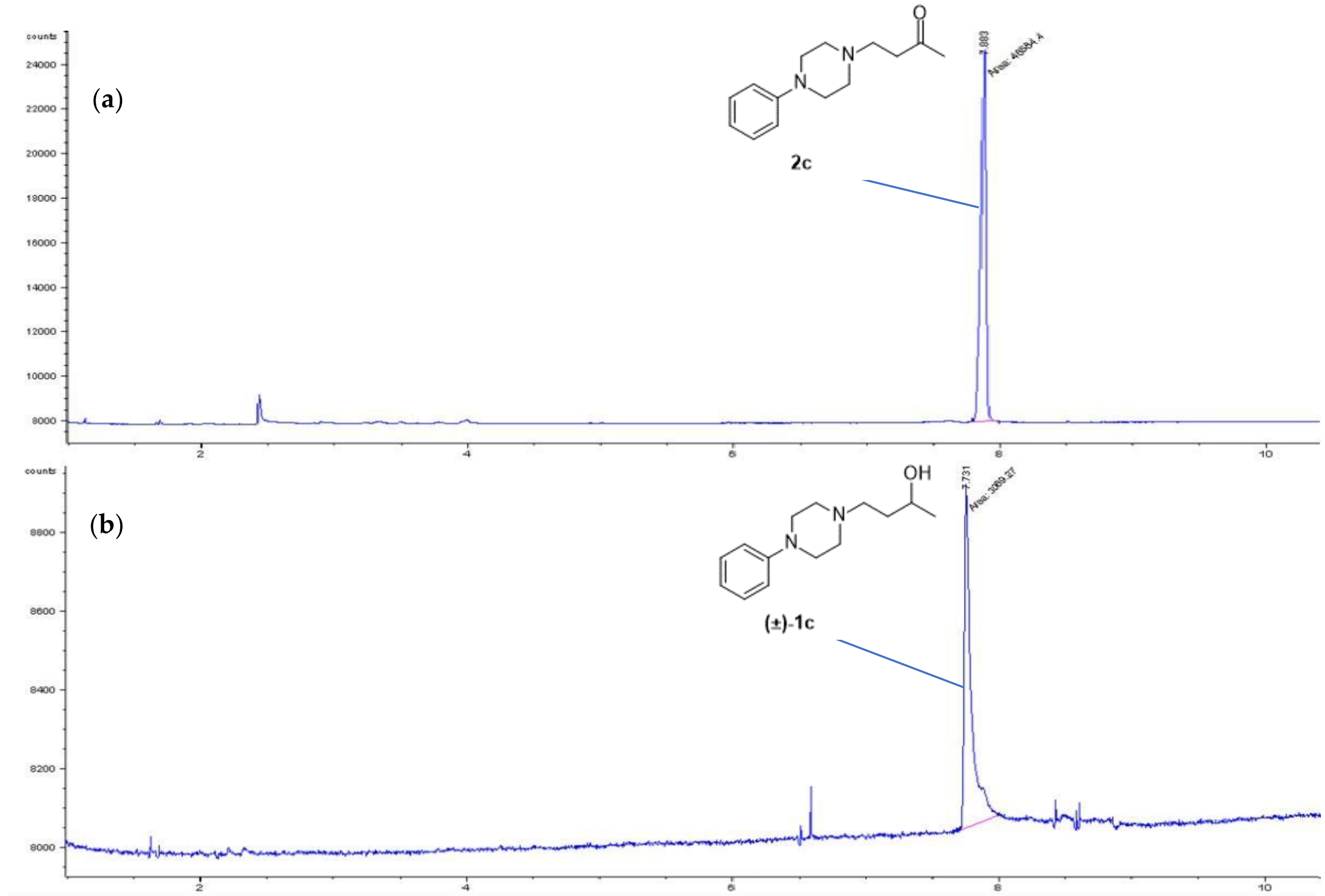

Fig. S4 Chromatogram of (**a**) 4-(4-phenylpiperazin-1-yl)butan-2-one **2c** and (**b**) 4-(4-phenylpiperazin-1-yl)butan-2-ol (**±)-1c**

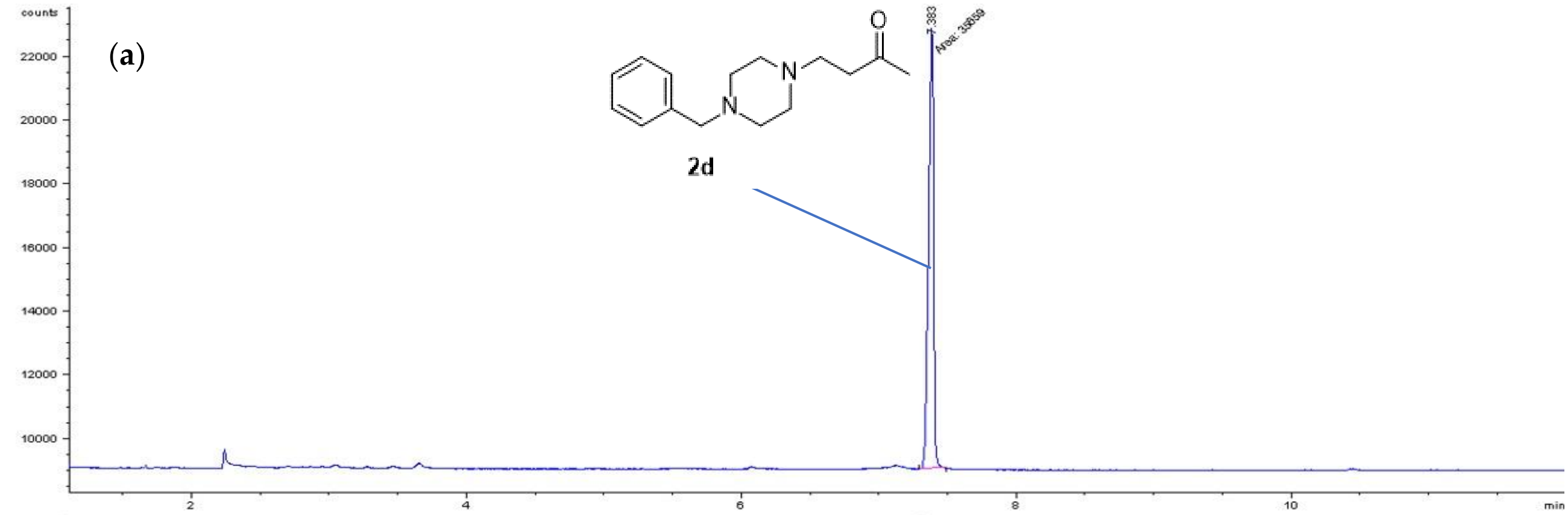

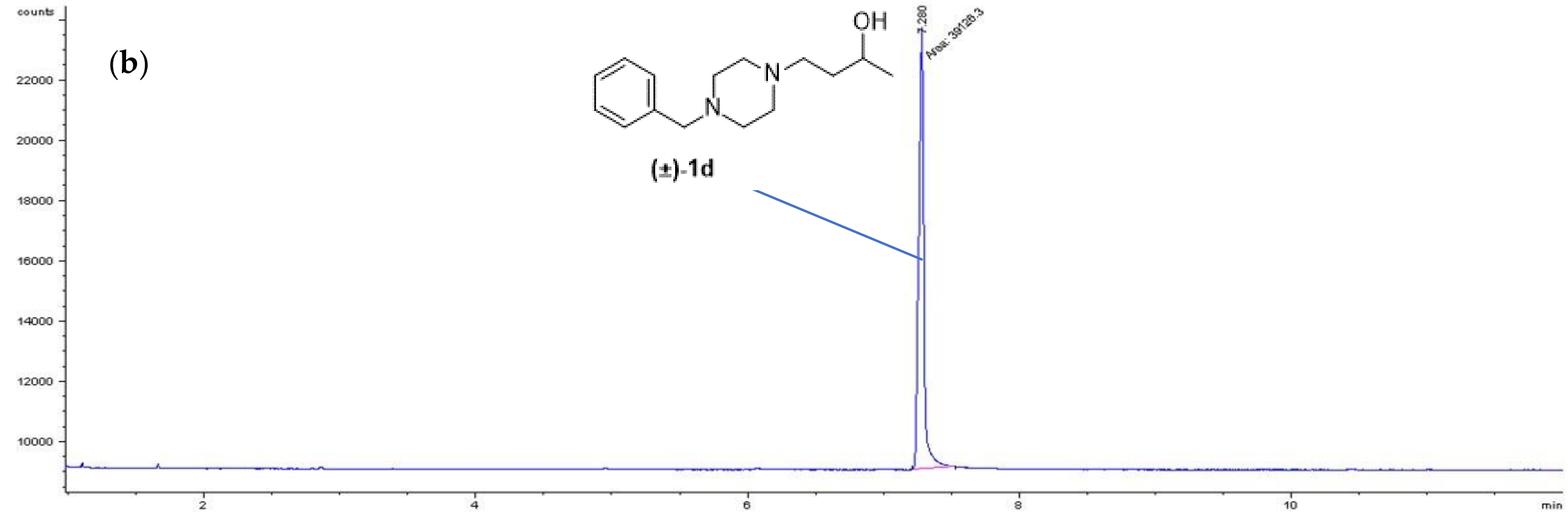

Fig. S5 Chromatogram of (**a**) 4-(4-benzylpiperazin-1-yl)butan-2-one **2d** and (**b**) 4-(4-benzylpiperazin-1-yl)butan-2-ol (±)**-1d**

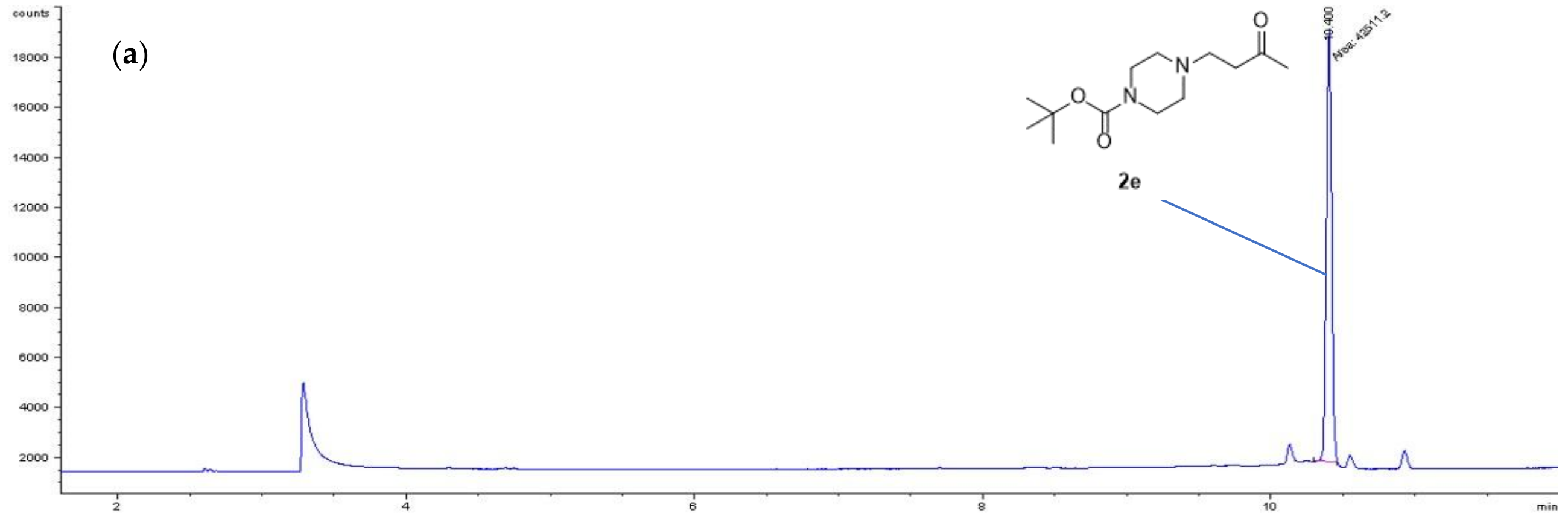

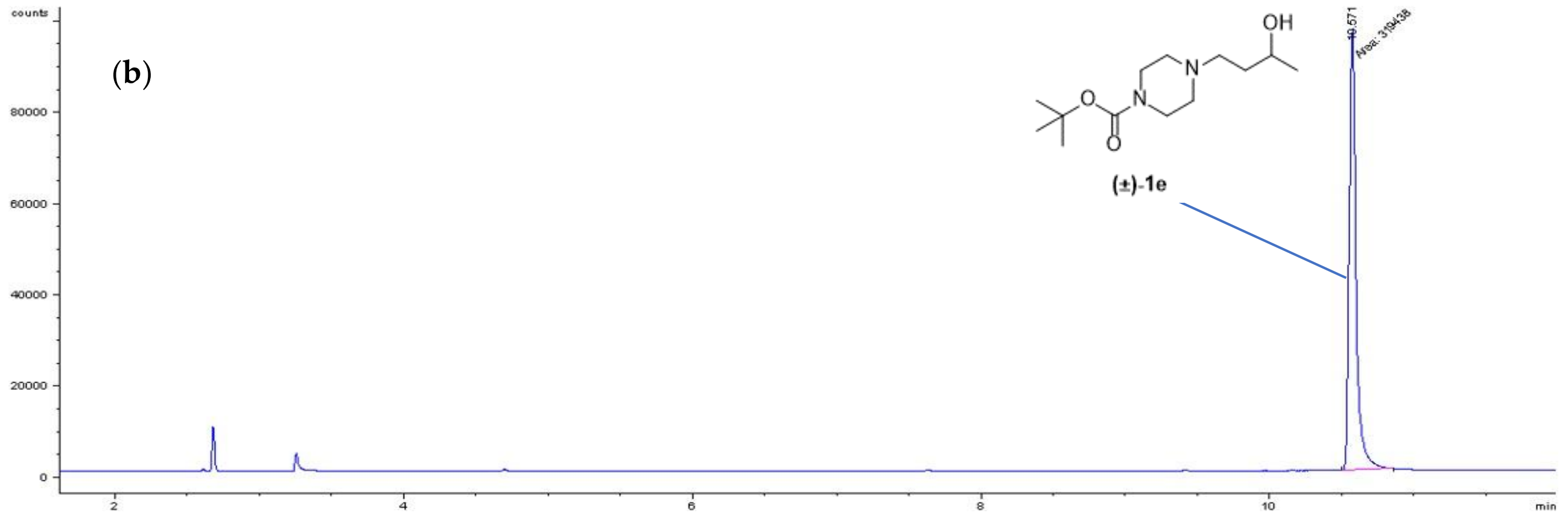

Fig. S6 Chromatogram of (**a**) *tert*-butyl 4-(3-oxobutyl)piperazine-1-carboxylate **2e** and (**b**) *tert*-butyl 4-(3-hydroxybutyl)piperazine-1-carboxylate (±)**-1e**

1.2.3. Gas chromatograms for determining the enantiomeric composition of the products

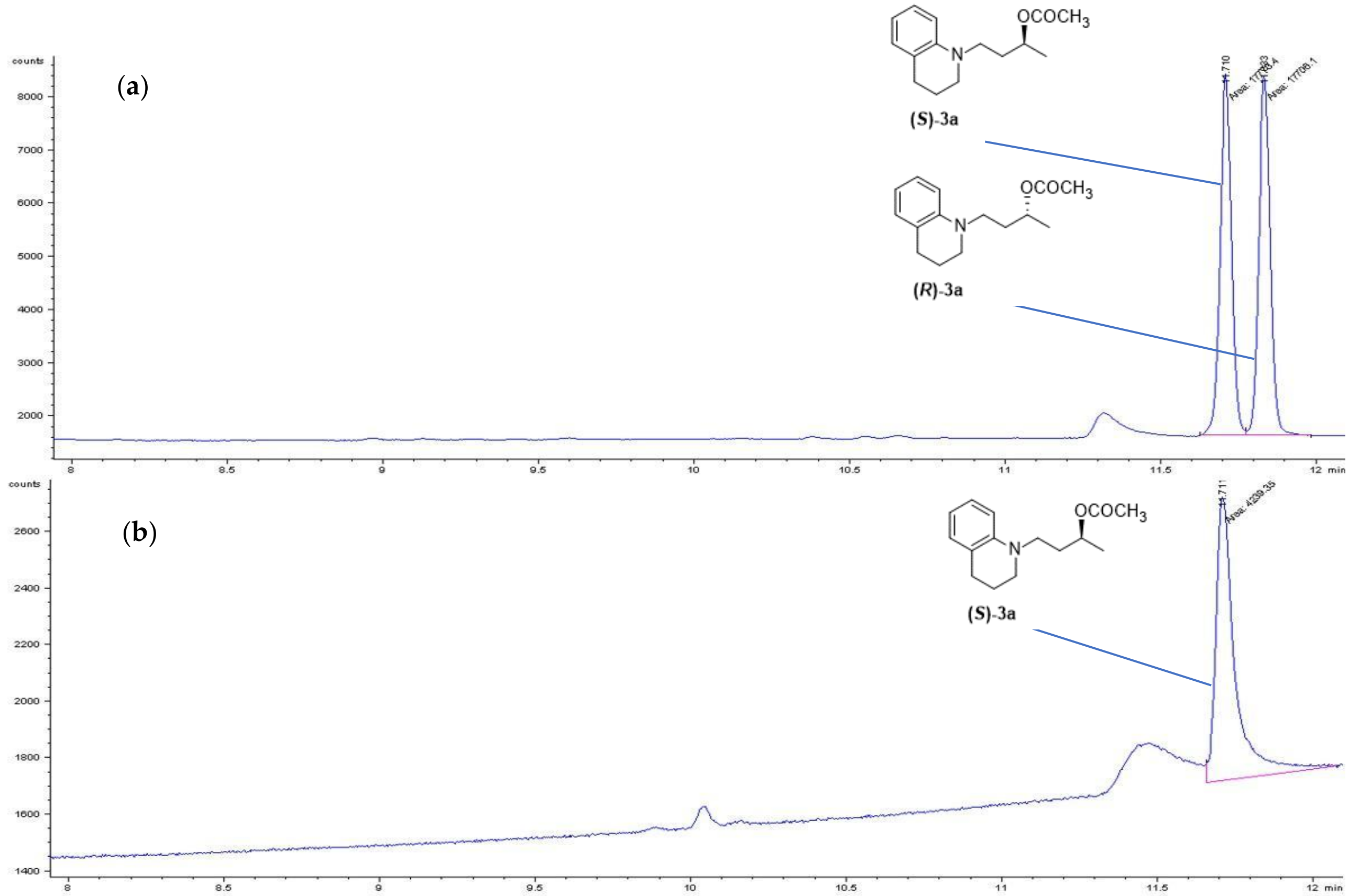

Fig. S7 Chromatogram of (**a**) racemic (±)-4-(3,4-dihydroquinolin-1(2*H*)-yl)butan-2-yl acetate [(*S*)-**3a** and (*R*)-**3a**]; (**b**) derivatized sample from bioreduction of 4-(3,4-dihydroquinolin-1(2*H*)-yl)butan-2-one **2a** with *Candida parapsilosis* (WY12) after 24 h

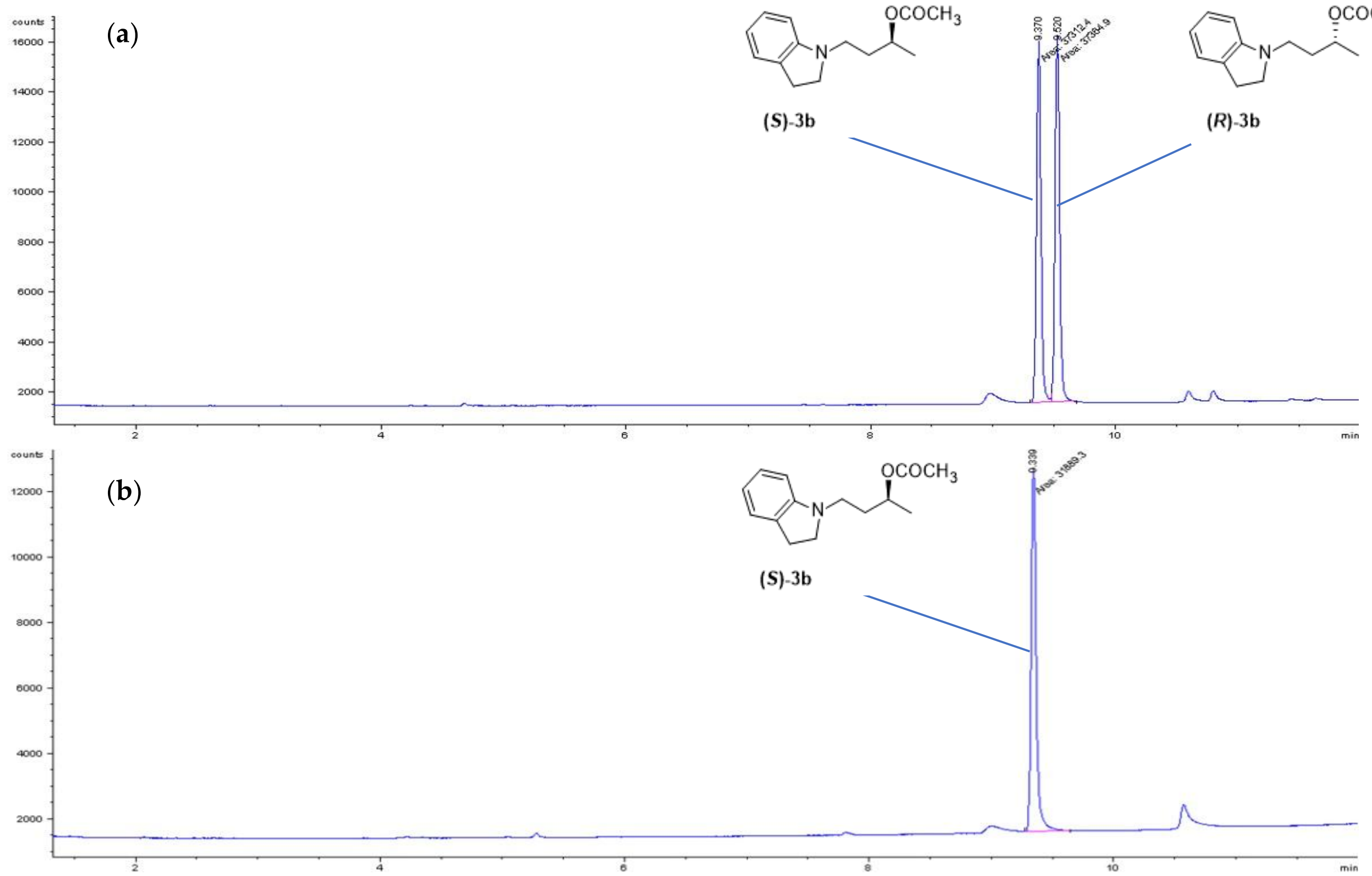

Fig. S8 Chromatogram of (**a**) racemic (±)-4-(indolin-1-yl)butan-2-yl acetate [(*S*)-**3b** and (*R*)-**3b**]; (**b**) derivatized sample from bioreduction of 4-(indolin-1-yl)butan-2-one **2b** with *Candida parapsilosis* (WY12) after 24 h

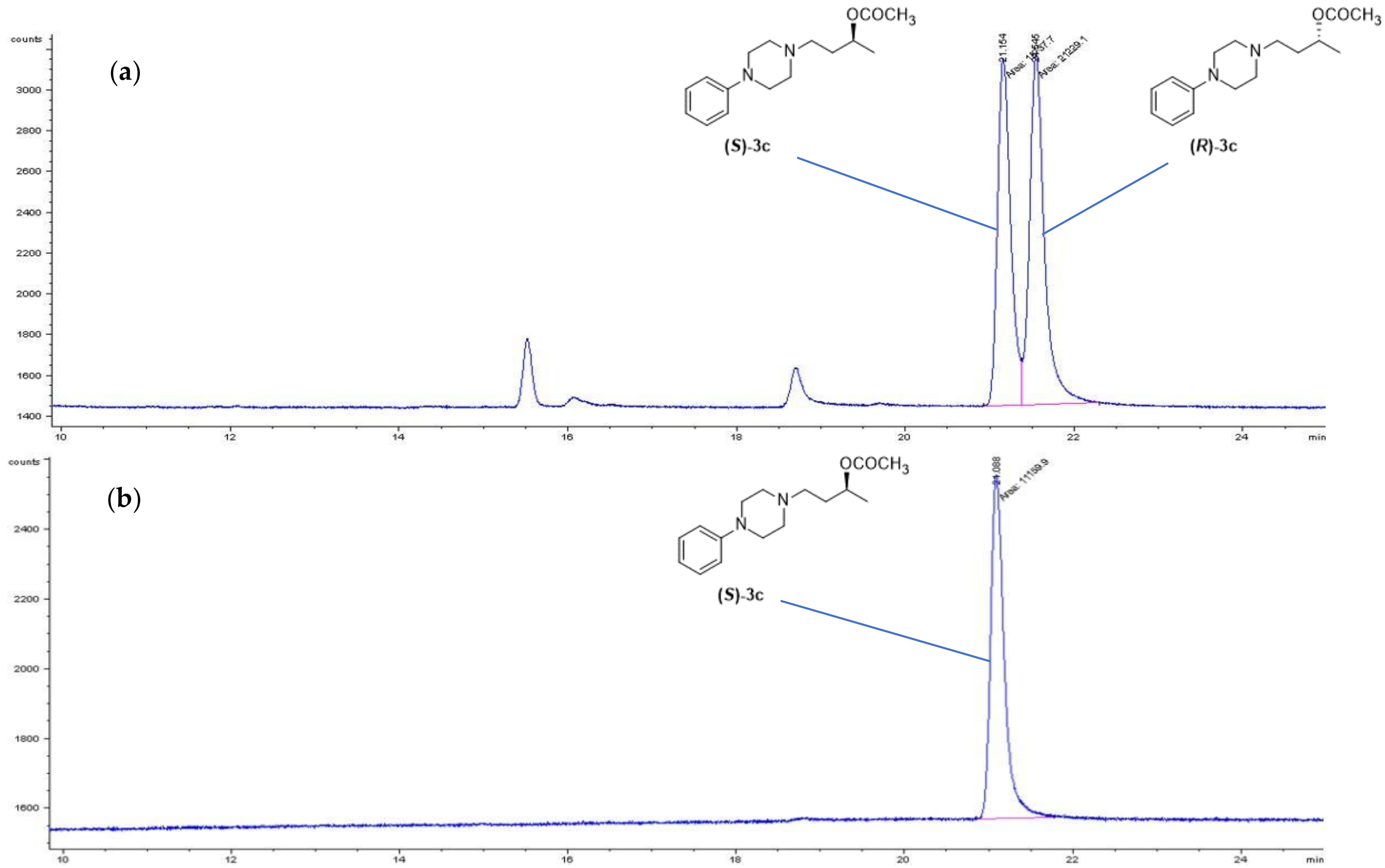

Fig. S9 Chromatogram of (**a**) racemic (±)-4-(4-phenylpiperazin-1-yl)butan-2-yl acetate [(*S*)-**3c** and (*R*)-**3c**]; (**b**) derivatized sample from bioreduction of 4-(4-phenylpiperazin-1-yl)butan-2-one **2c** with *Candida parapsilosis* (WY12) after 24 h

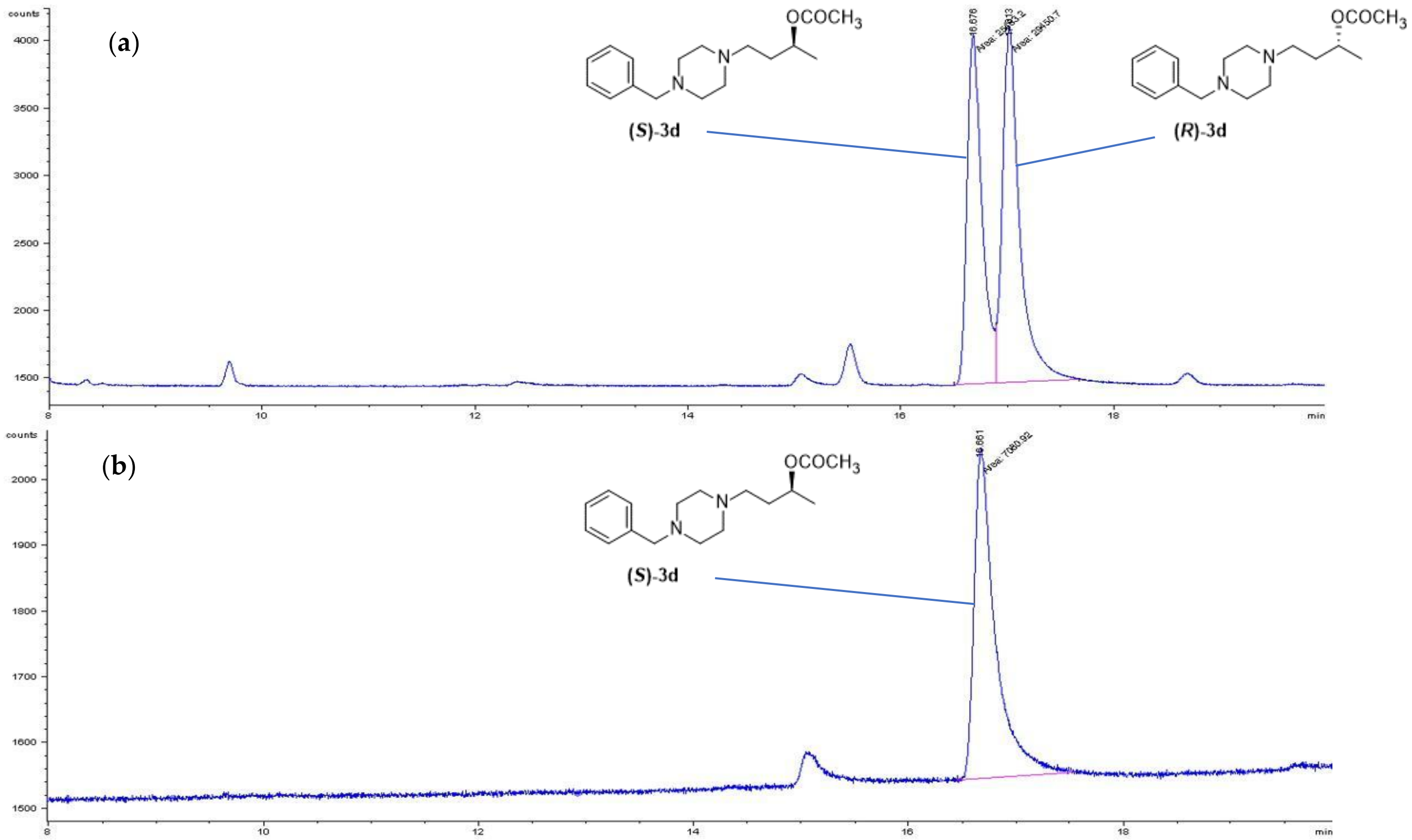

Fig. S10 Chromatogram of (**a**) racemic (±)-4-(4-benzylpiperazin-1-yl)butan-2-yl acetate [(*S*)-**3d** and (*R*)-**3d**]; (**b**) derivatized sample from bioreduction of 4-(4-benzylpiperazin-1-yl)butan-2-one **2d** with *Candida parapsilosis* (WY12) after 24 h

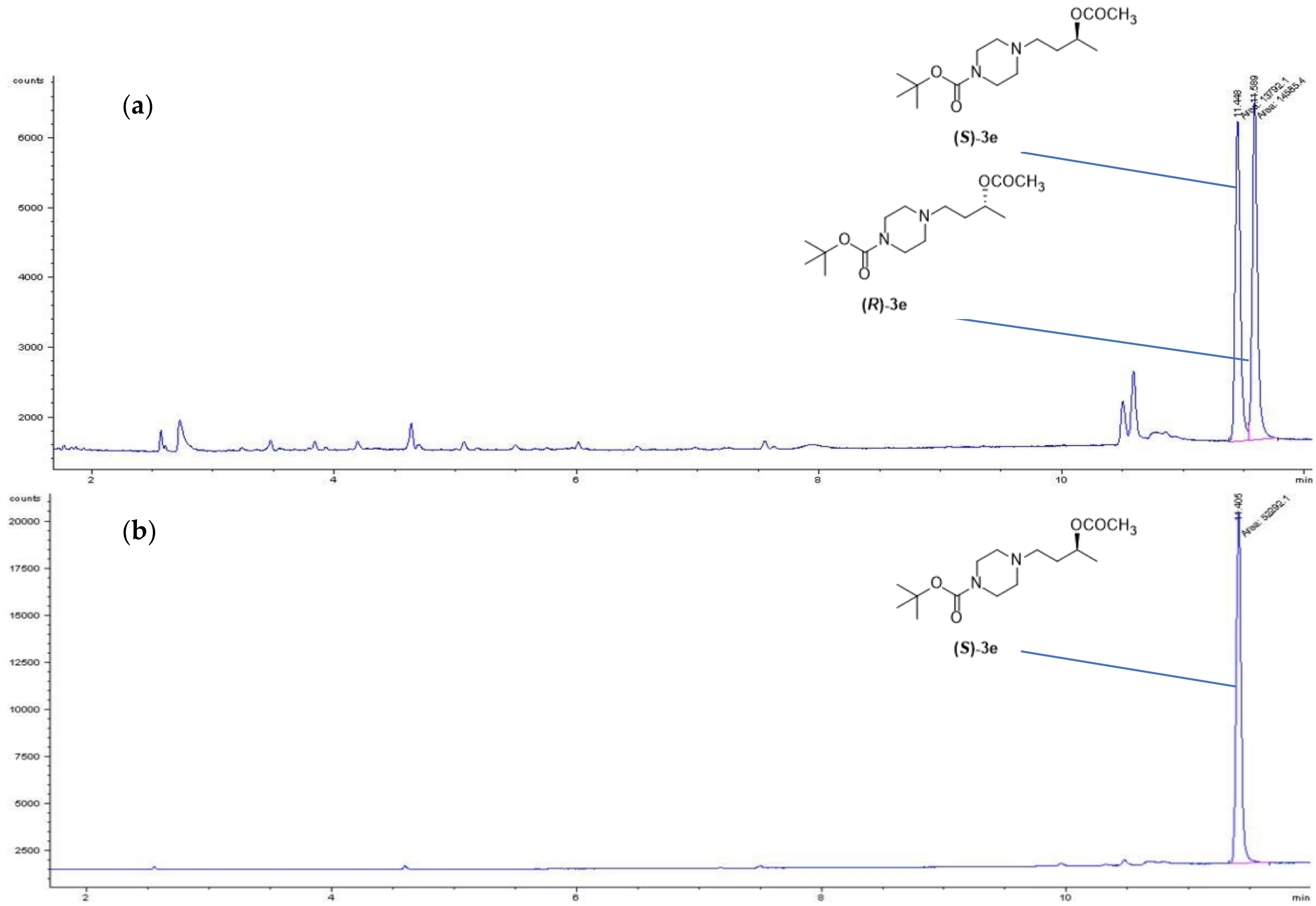

Fig. S11 Chromatogram of (**a**) racemic (±)-*tert*-butyl 4-(3-acetoxybutyl)piperazine-1-carboxylate [(*S*)-**3e** and (*R*)-**3e**]; (**b**) derivatized sample from bioreduction of *tert*-butyl 4-(3-oxobutyl)piperazine-1-carboxylate **2e** with *Candida parapsilosis* (WY12) after 24 h

2. Spectra of the starting materials and products

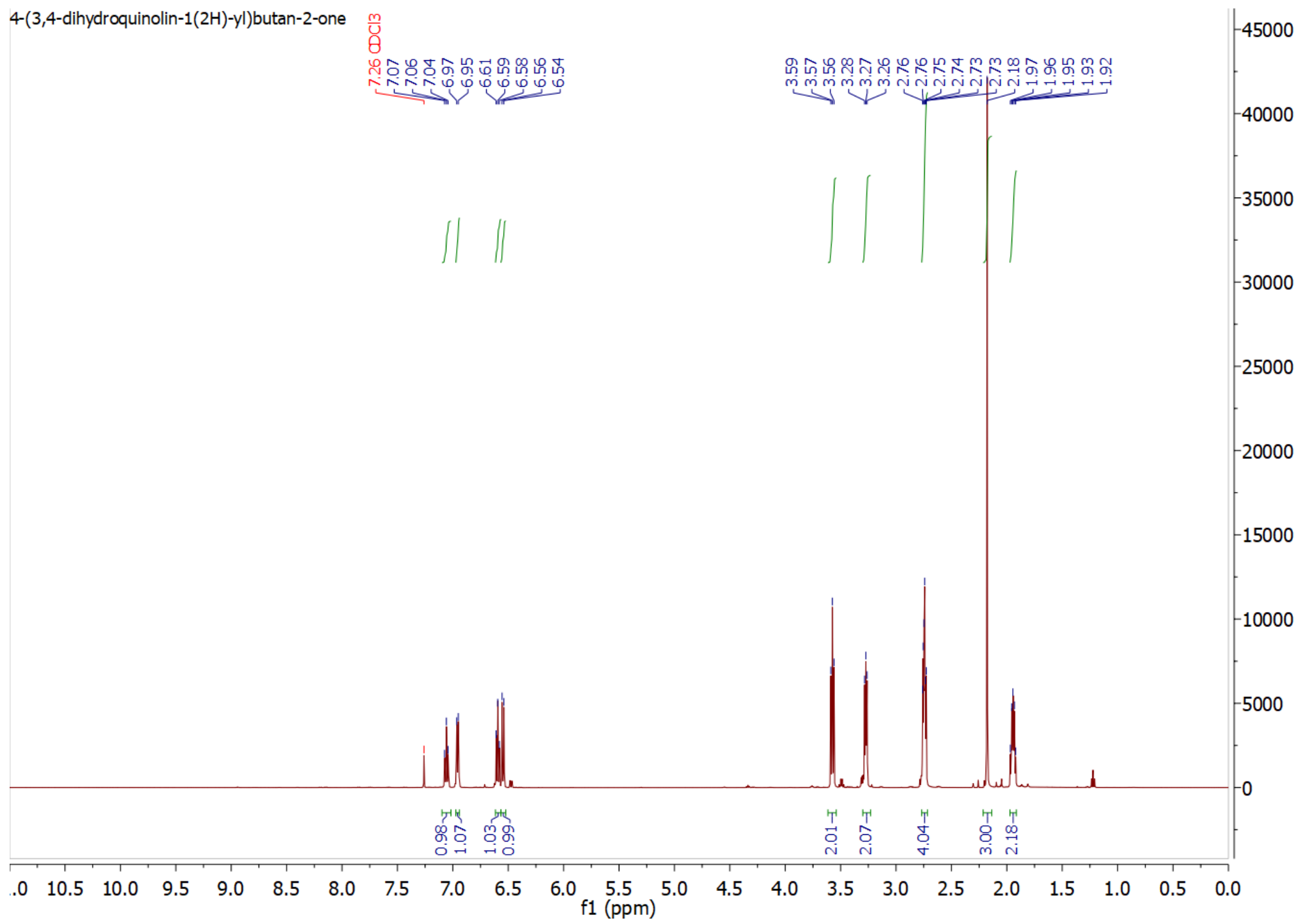

Fig. S12 ^{1}H NMR spectrum of 4-(3,4-dihydroquinolin-1(2*H*)-yl)butan-2-one **2a**

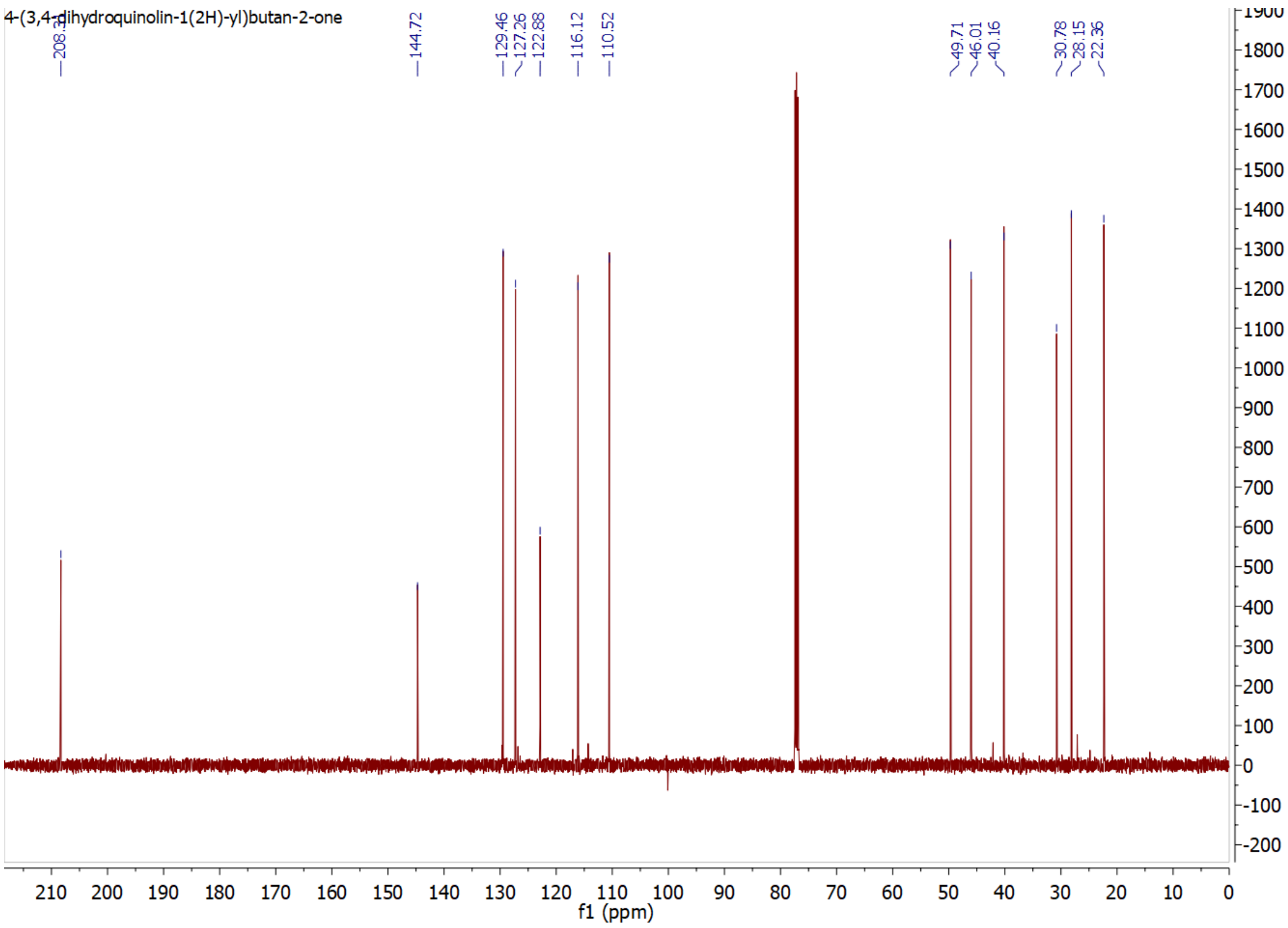

Fig. S13 ^{13}C NMR spectrum of 4-(3,4-dihydroquinolin-1(2*H*)-yl)butan-2-one **2a**

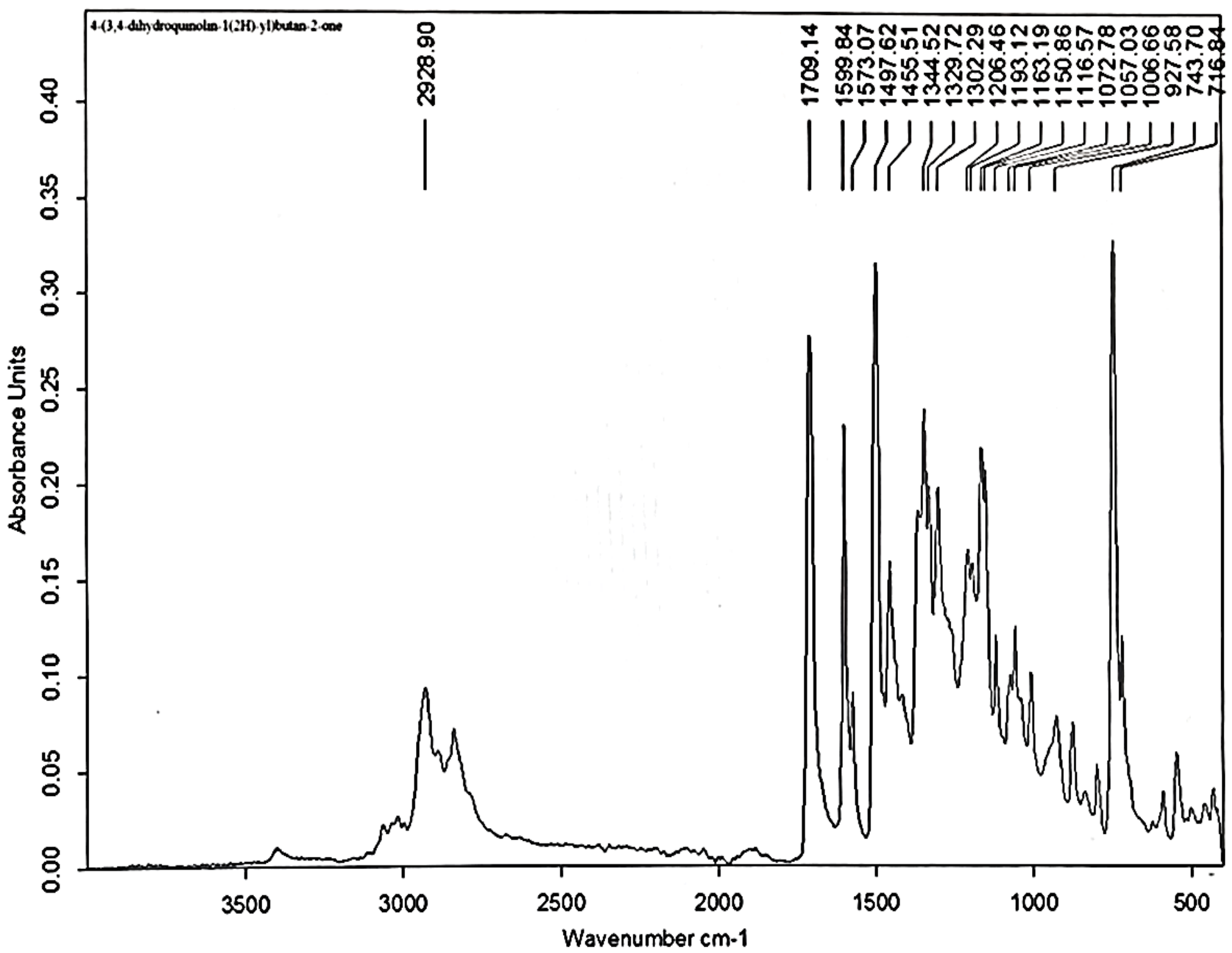

Fig. S14 IR spectrum of 4-(3,4-dihydroquinolin-1(2*H*)-yl)butan-2-one **2a**

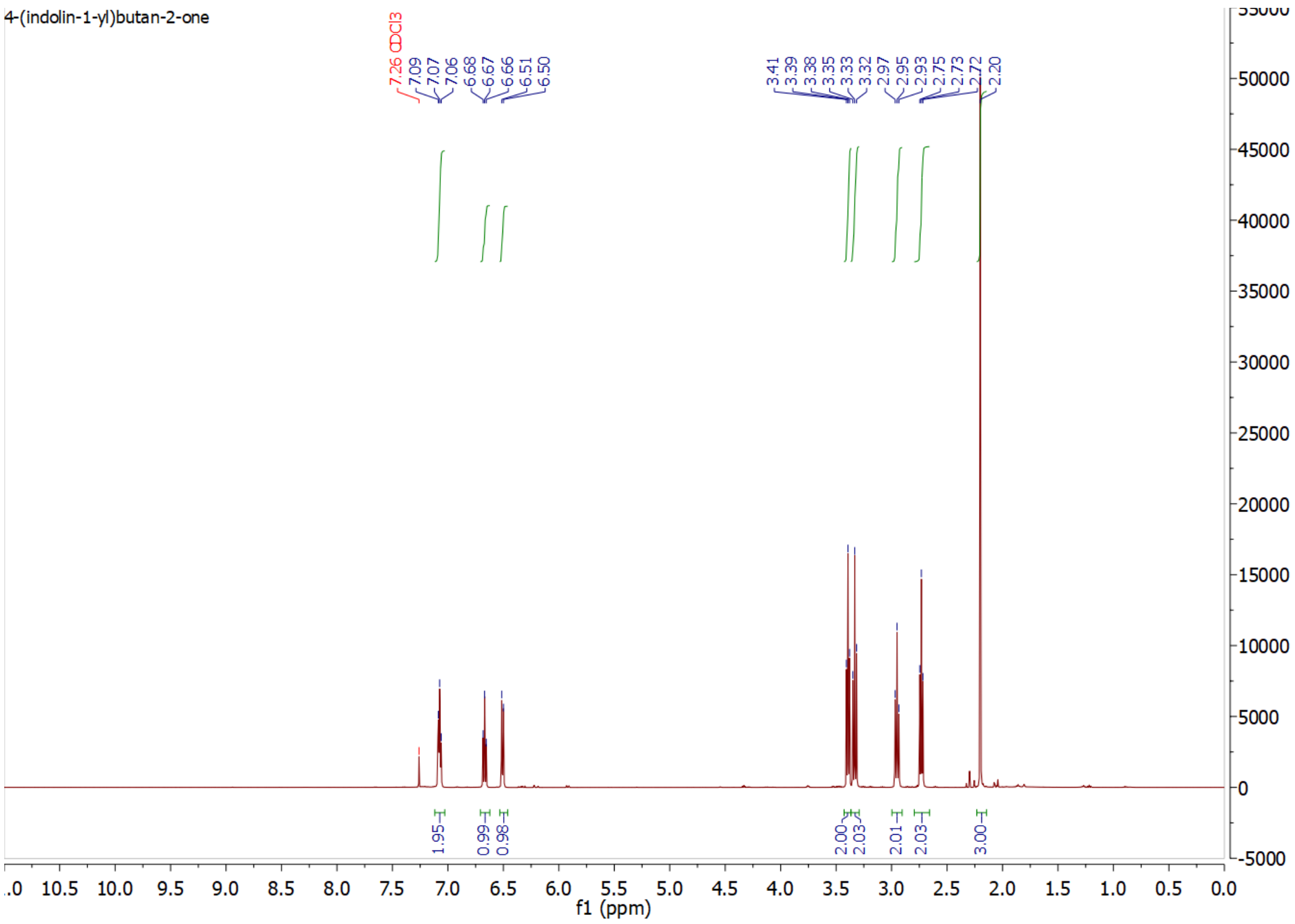

Fig. S15 ^{1}H NMR spectrum of 4-(indolin-1-yl)butan-2-one **2b**

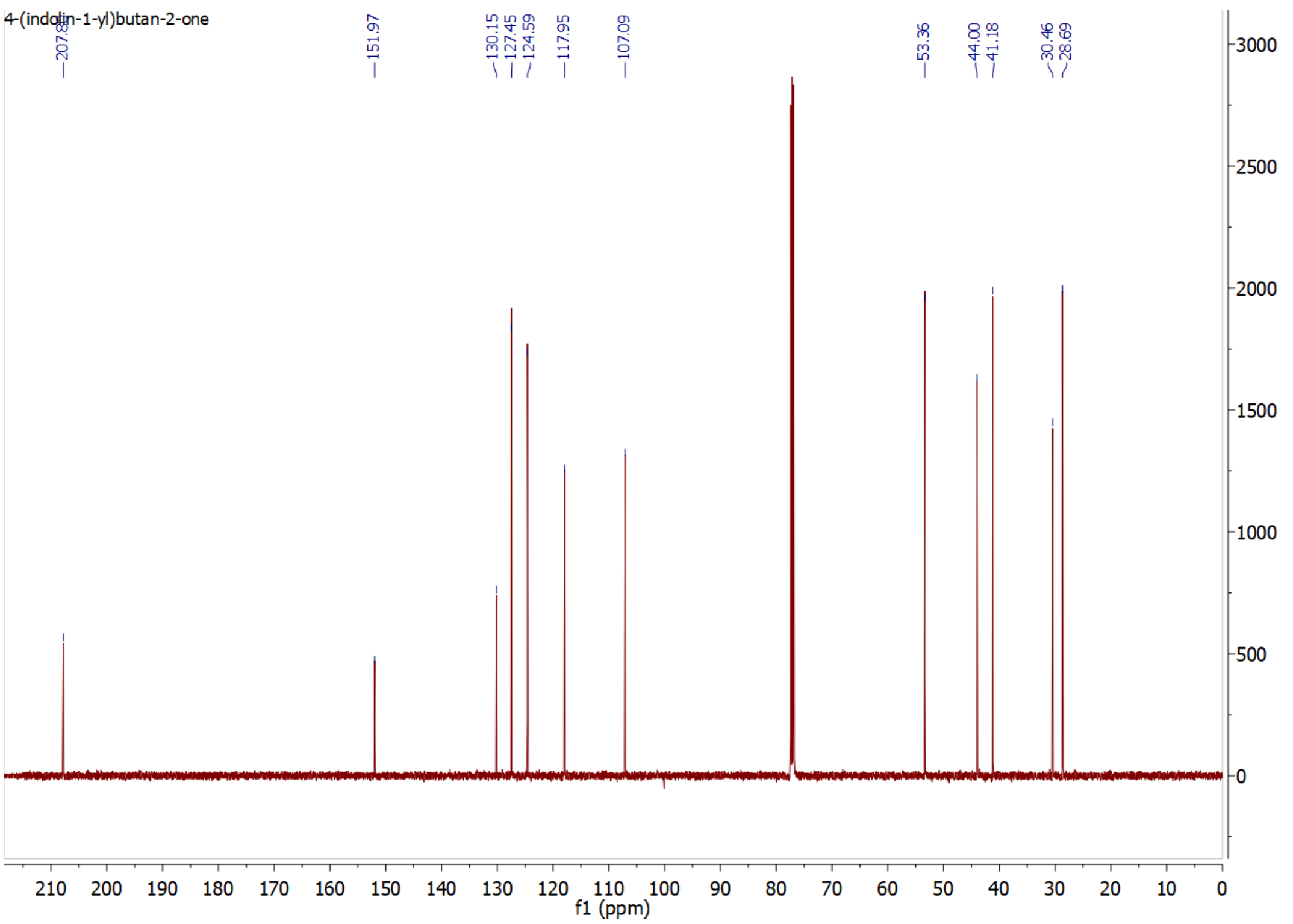

Fig. S16 ^{13}C NMR spectrum of 4-(indolin-1-yl)butan-2-one **2b**

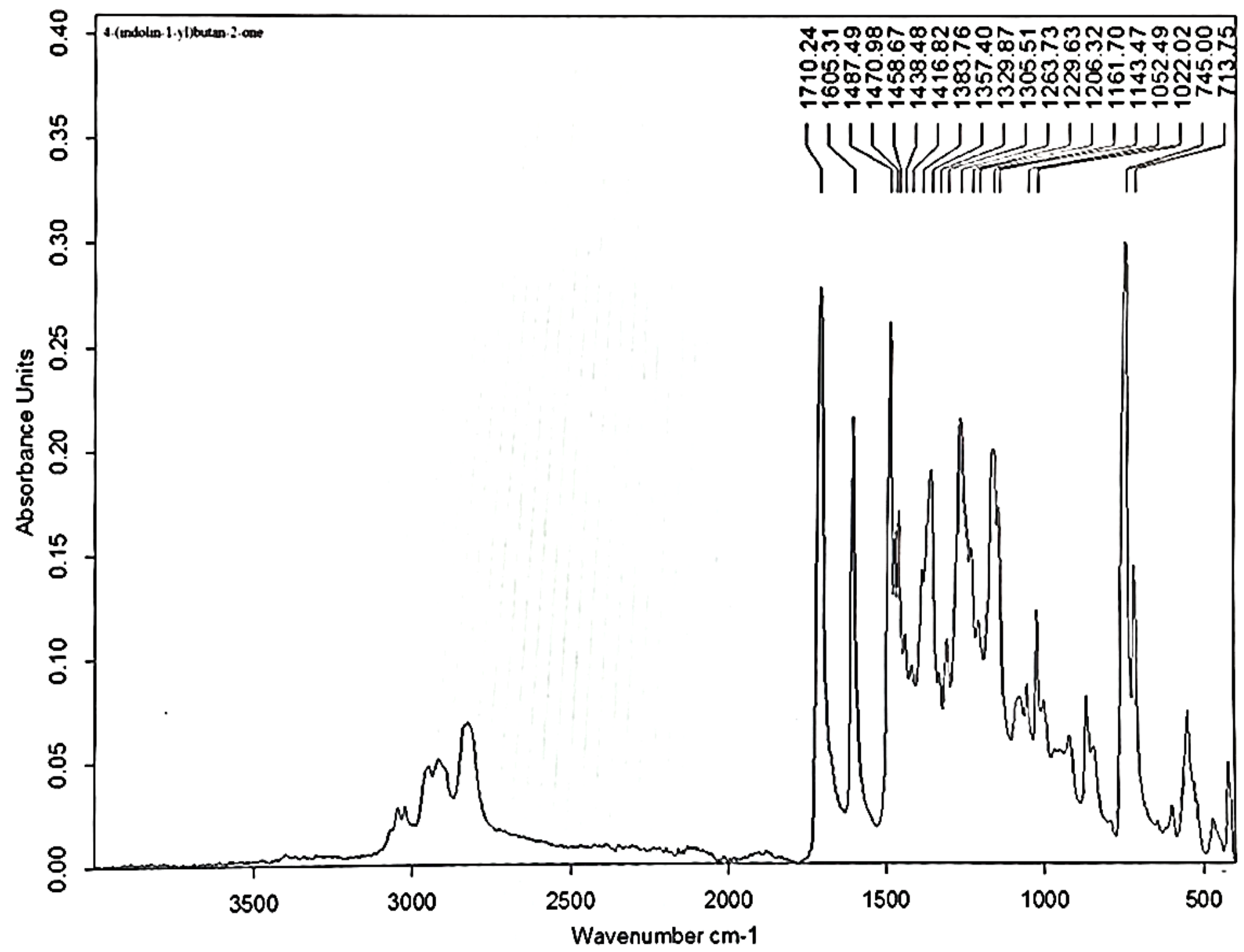

Fig. S17 IR spectrum of 4-(indolin-1-yl)butan-2-one **2b**

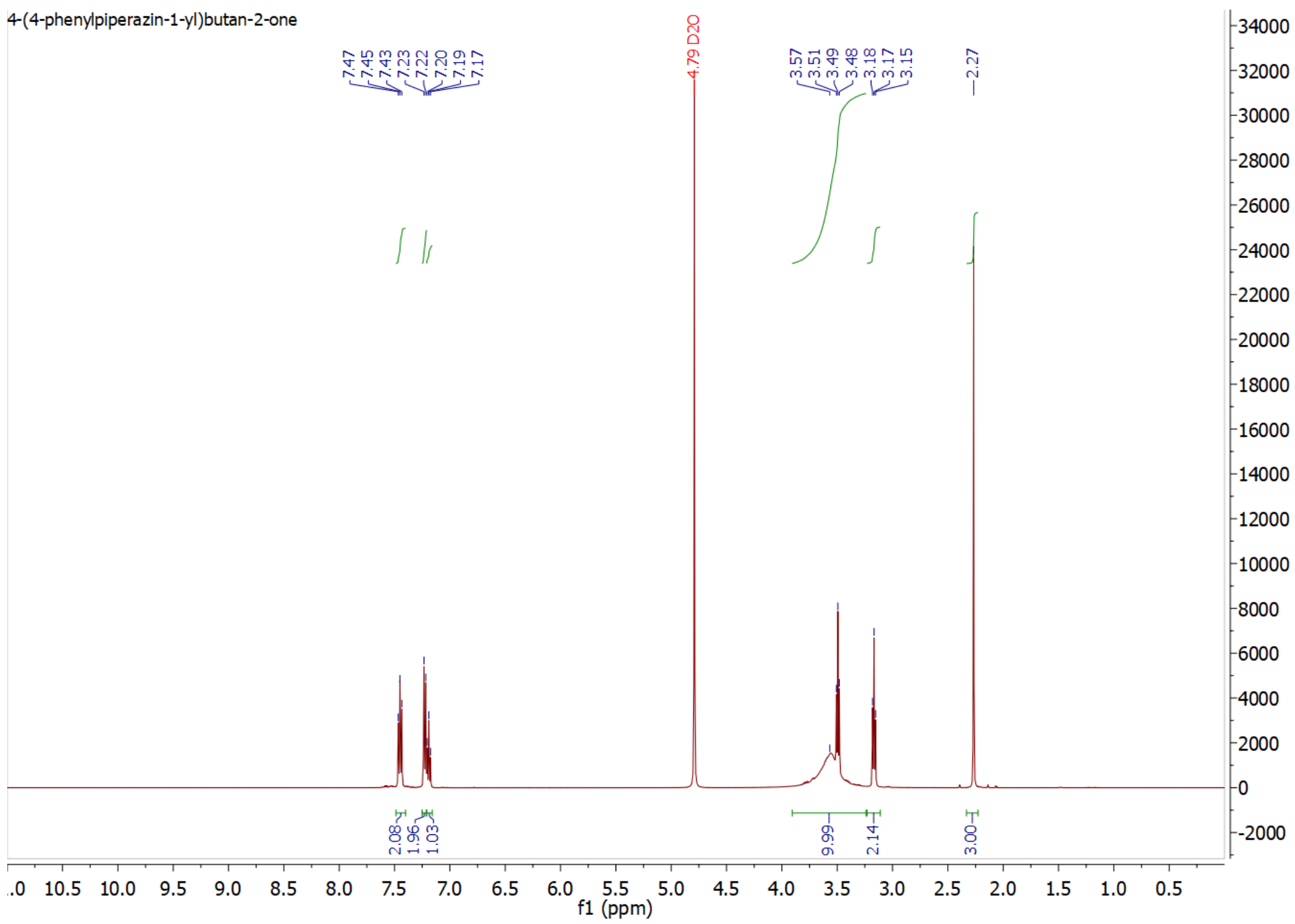

Fig. S18 ^{1}H NMR spectrum of 4-(4-phenylpiperazin-1-yl)butan-2-one **2c**

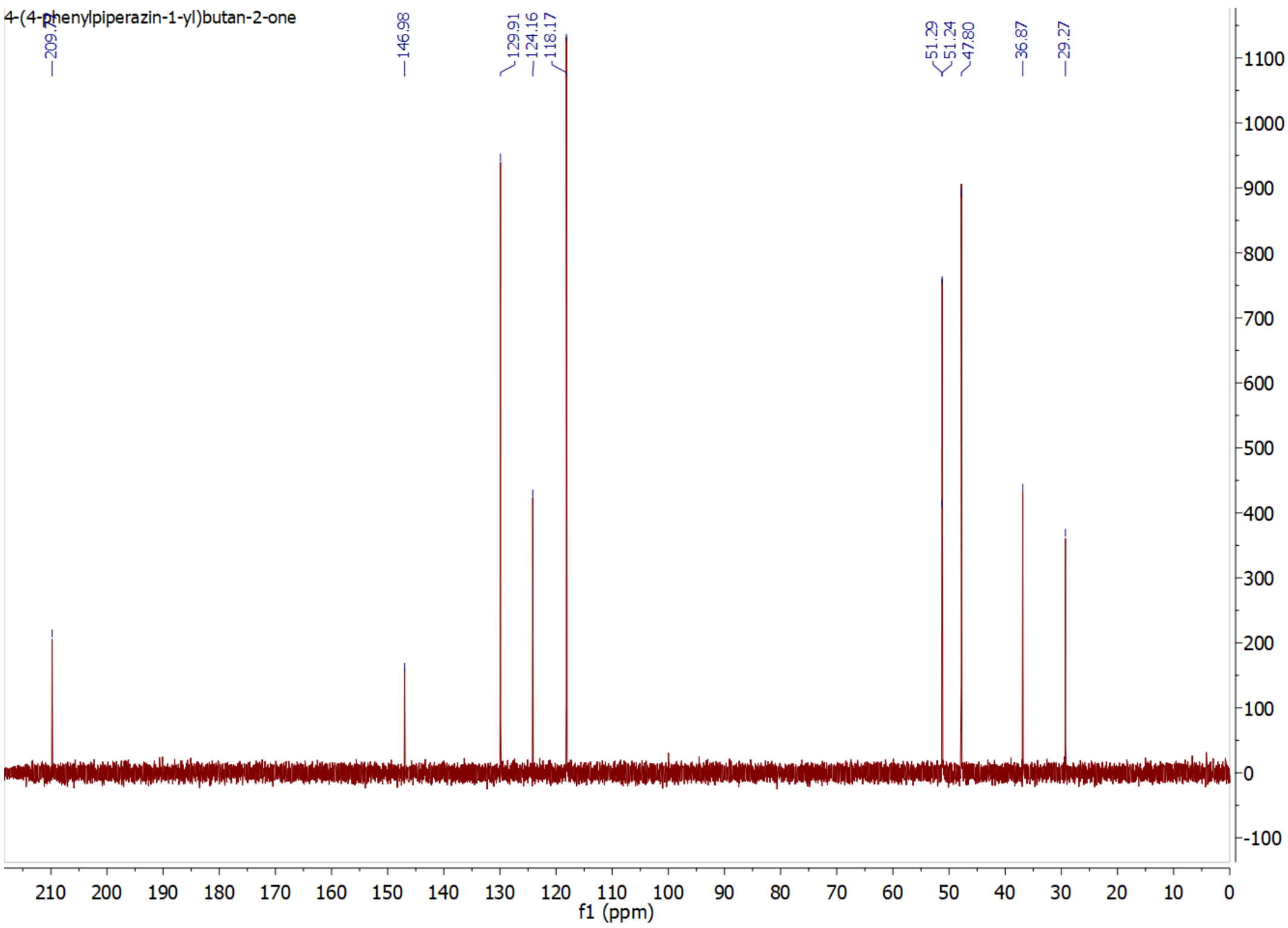

Fig. S19 ^{13}C NMR spectrum of 4-(4-phenylpiperazin-1-yl)butan-2-one **2c**

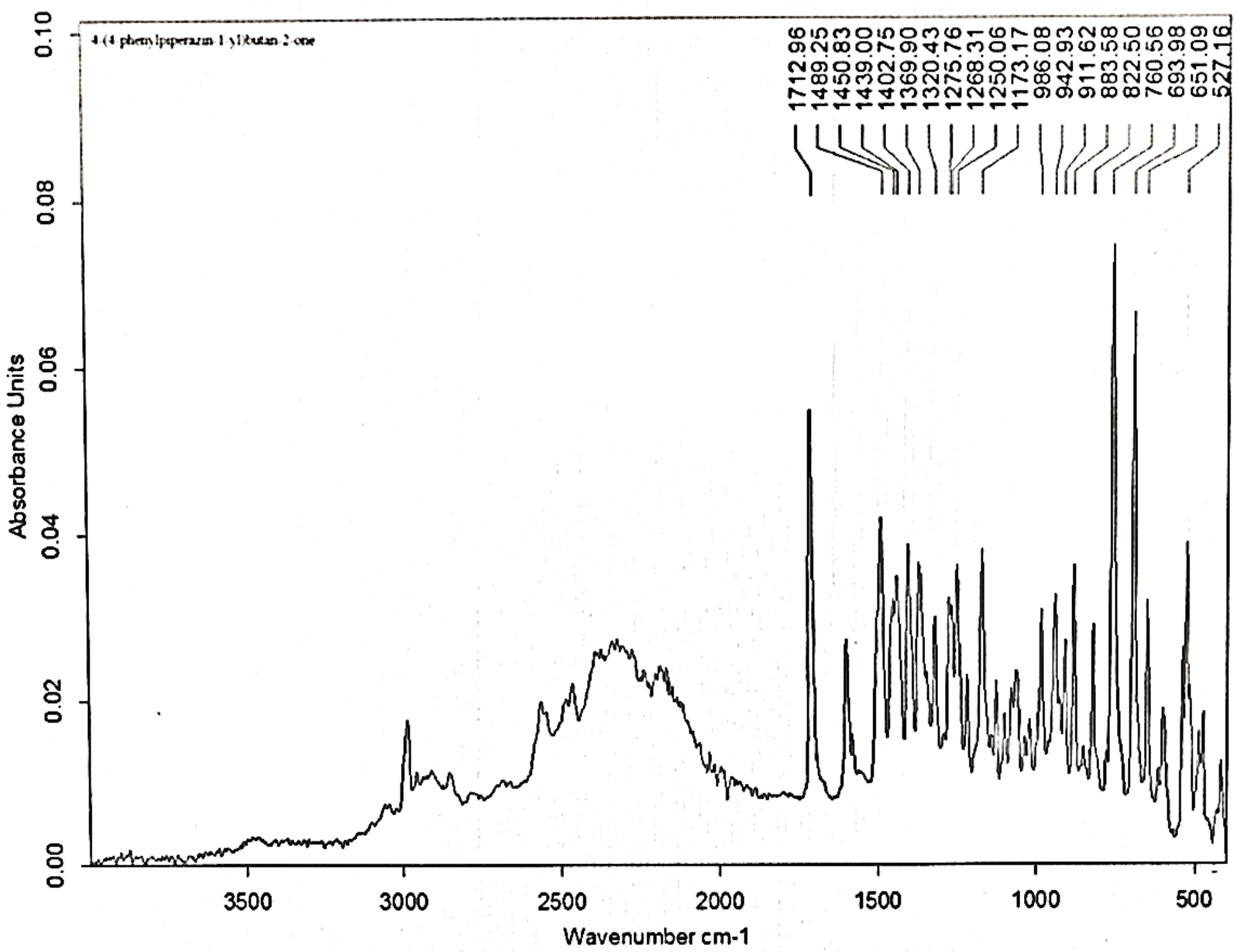

Fig. S20 IR spectrum of 4-(4-phenylpiperazin-1-yl)butan-2-one **2c**

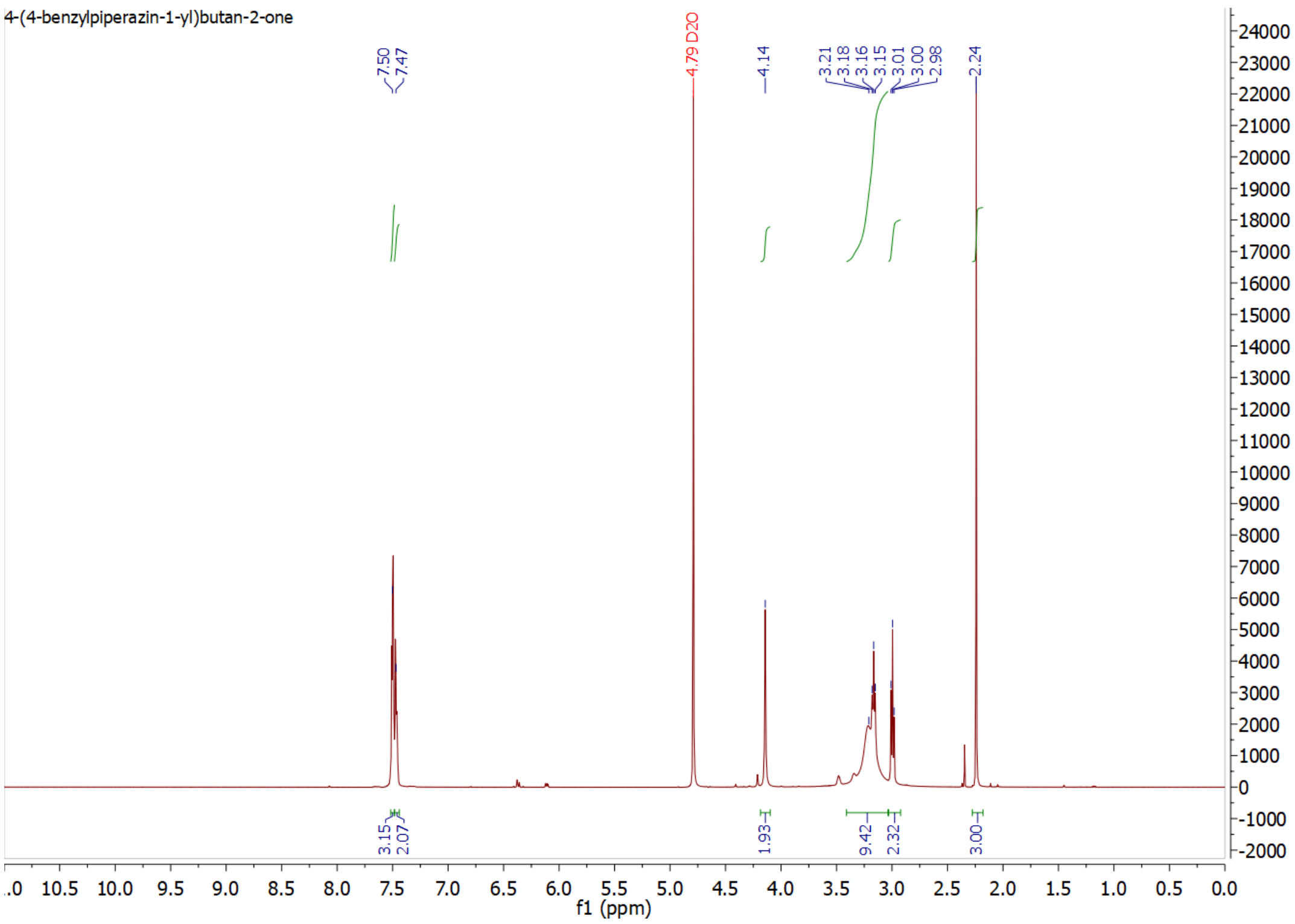

Fig. S21 ^{1}H NMR spectrum of 4-(4-benzylpiperazin-1-yl)butan-2-one **2d**

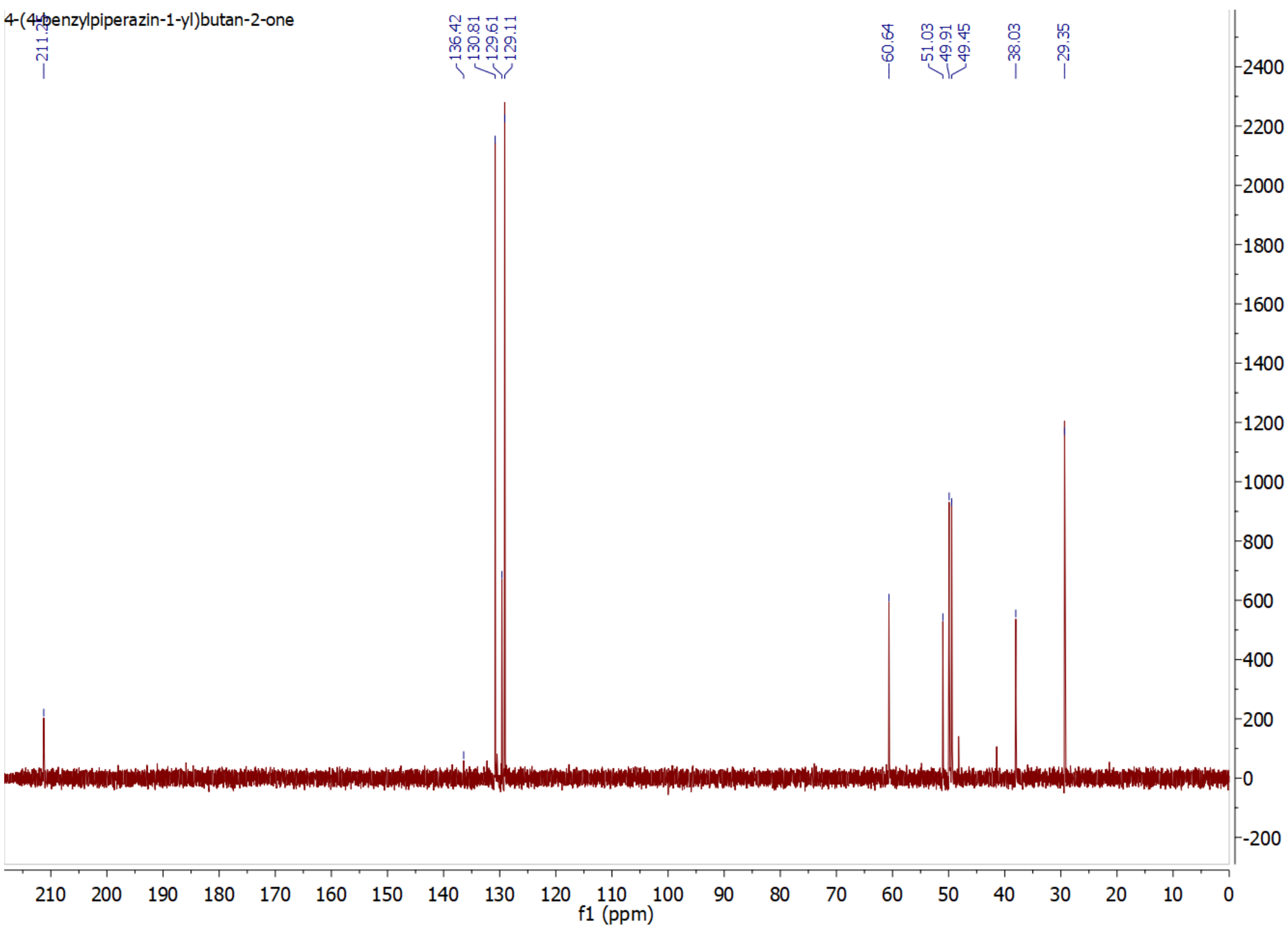

Fig. S22 ^{13}C NMR spectrum of 4-(4-benzylpiperazin-1-yl)butan-2-one **2d**

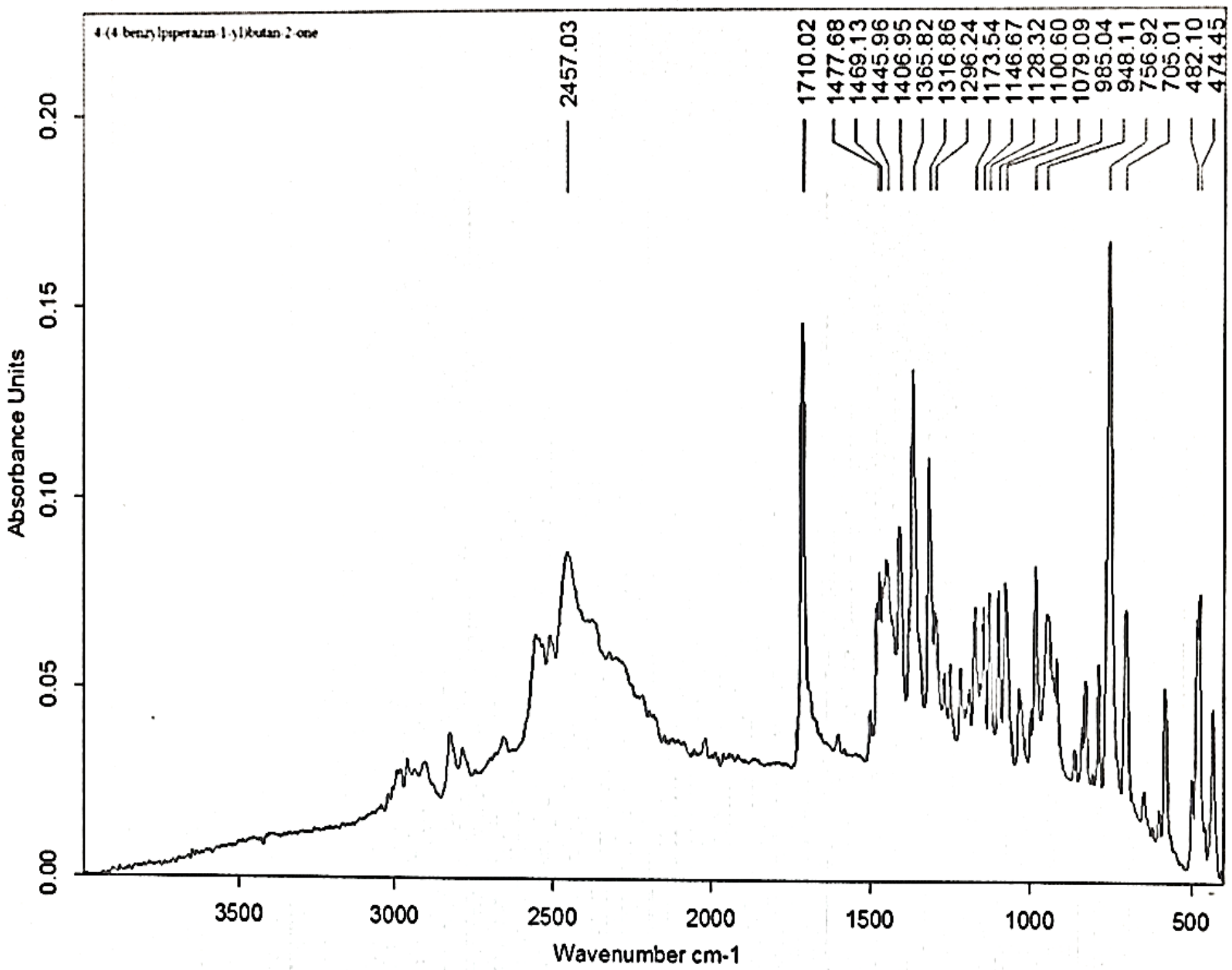

Fig. S23 IR spectrum of 4-(4-benzylpiperazin-1-yl)butan-2-one **2d**

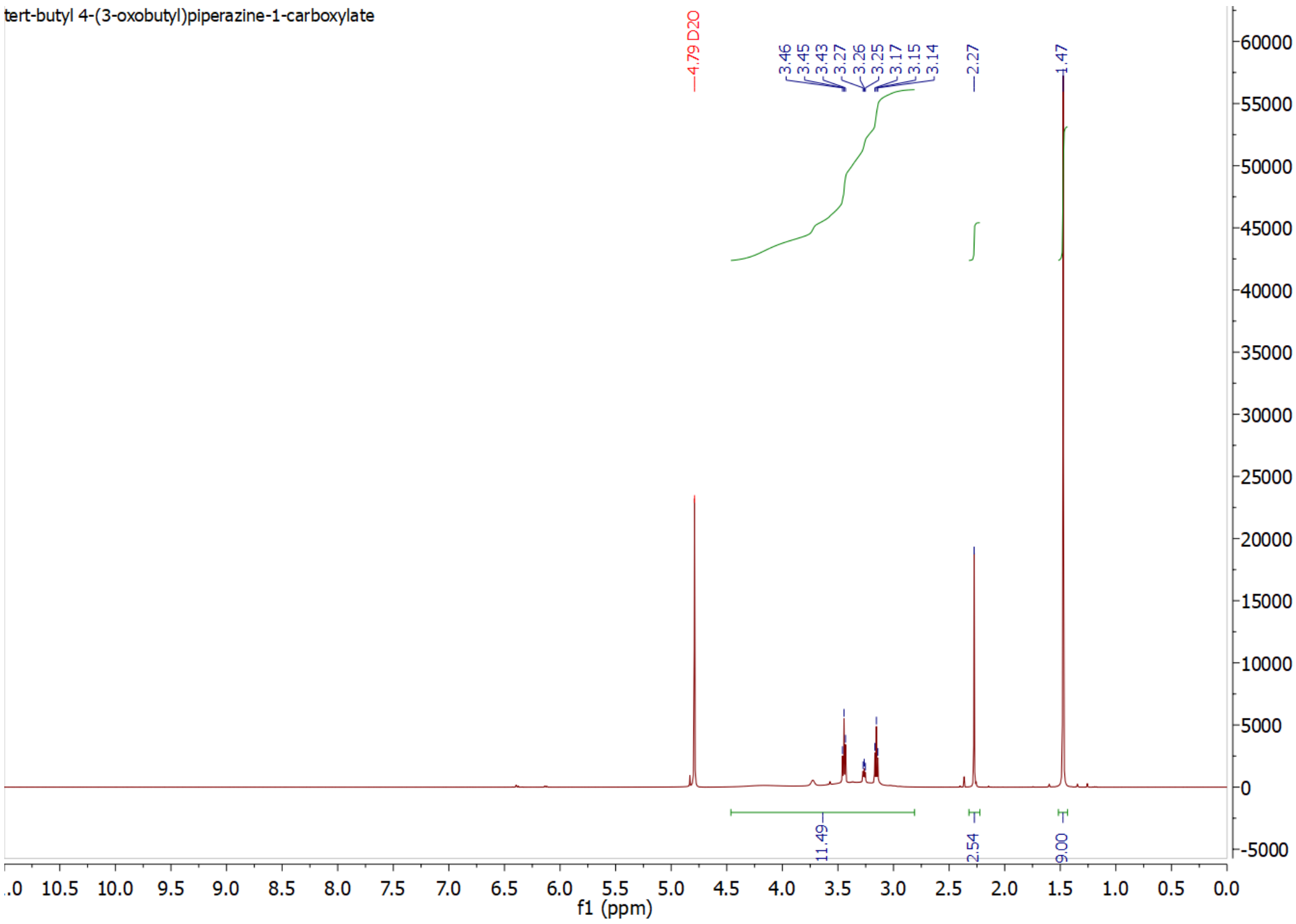

Fig. S24 ^{1}H NMR spectrum of *tert*-butyl 4-(3-oxobutyl)piperazine-1-carboxylate **2e**

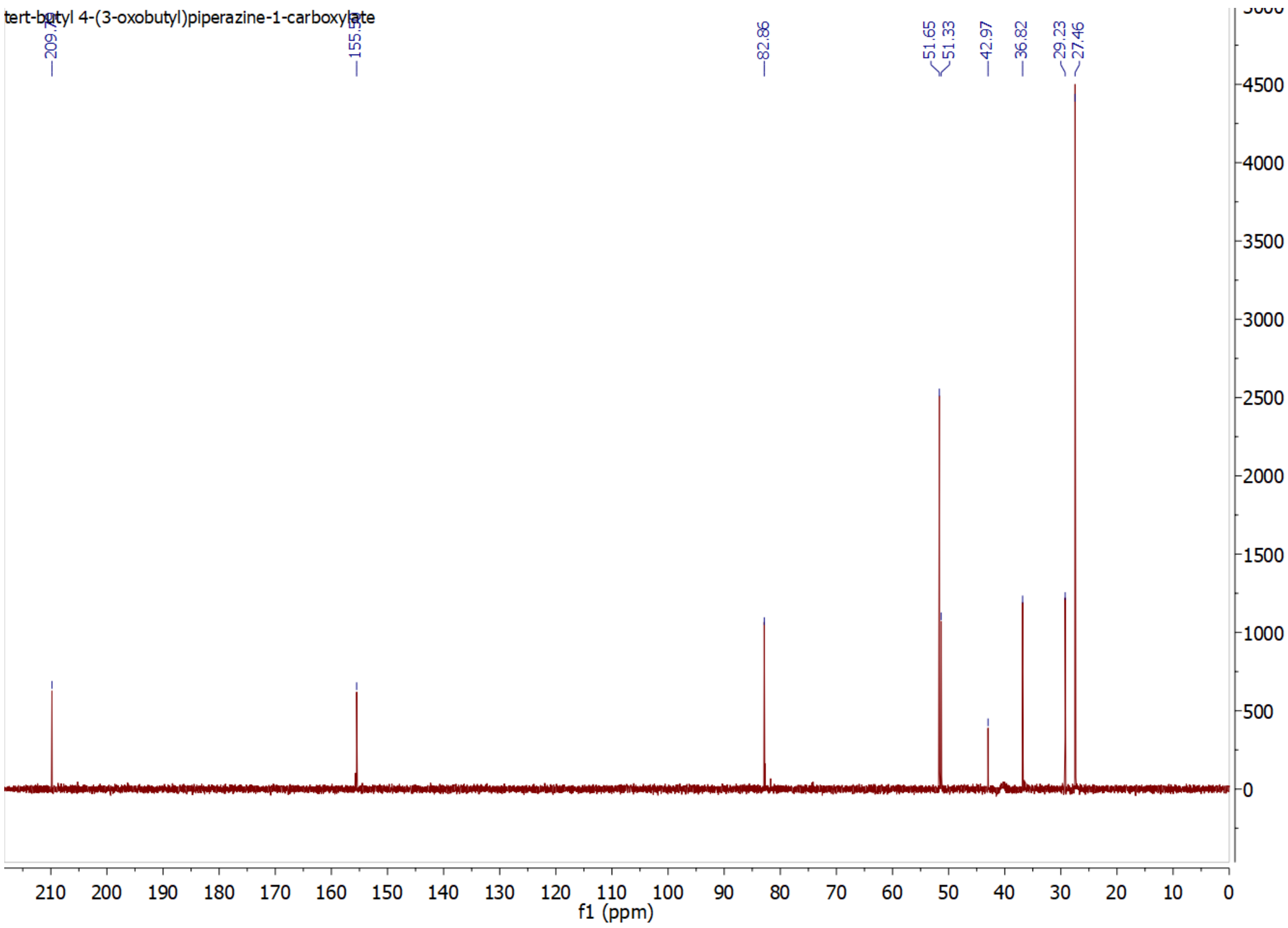

Fig. S25 ^{13}C NMR spectrum of *tert*-butyl 4-(3-oxobutyl)piperazine-1-carboxylate **2e**

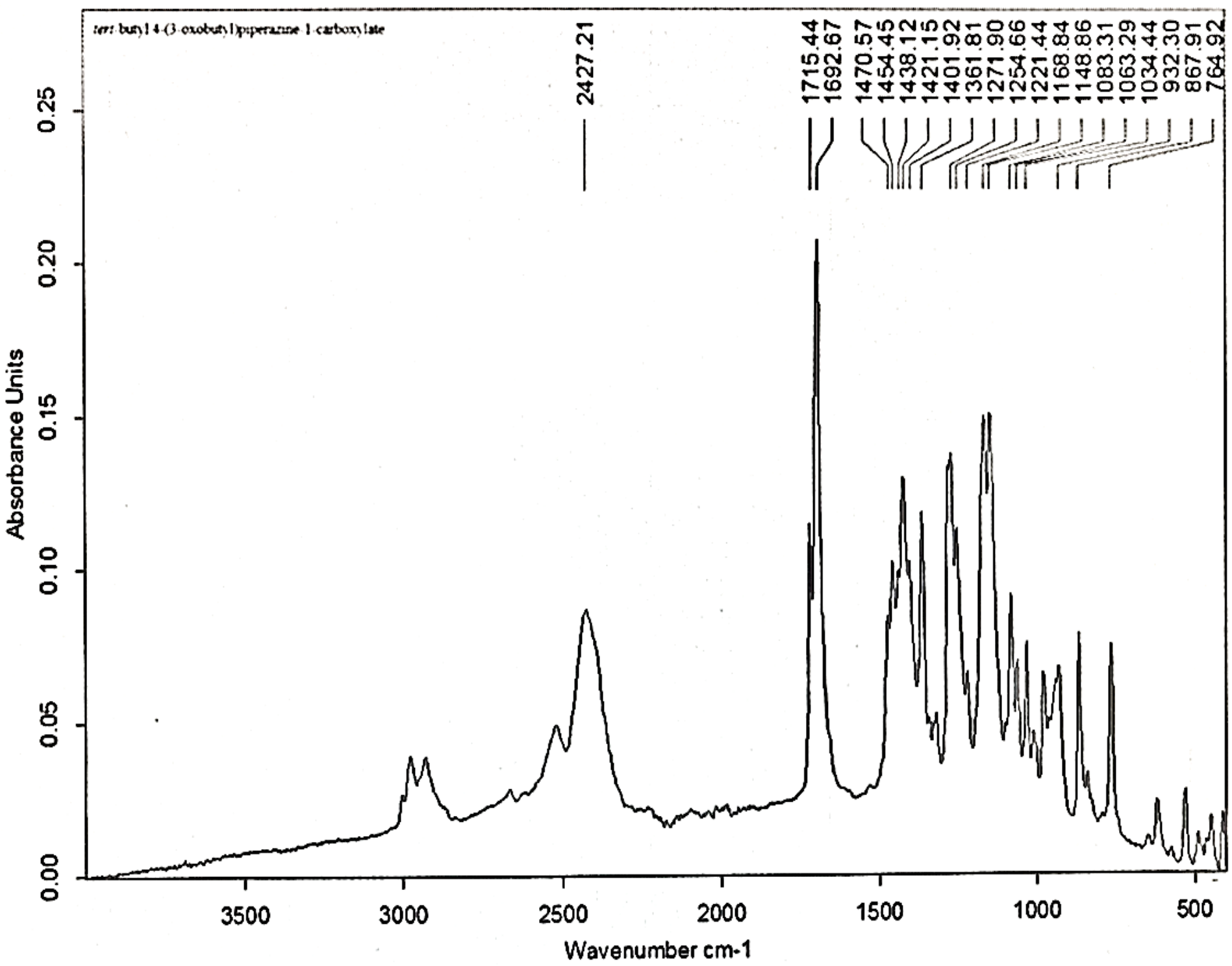

Fig. S26 IR spectrum of *tert*-butyl 4-(3-oxobutyl)piperazine-1-carboxylate **2e**

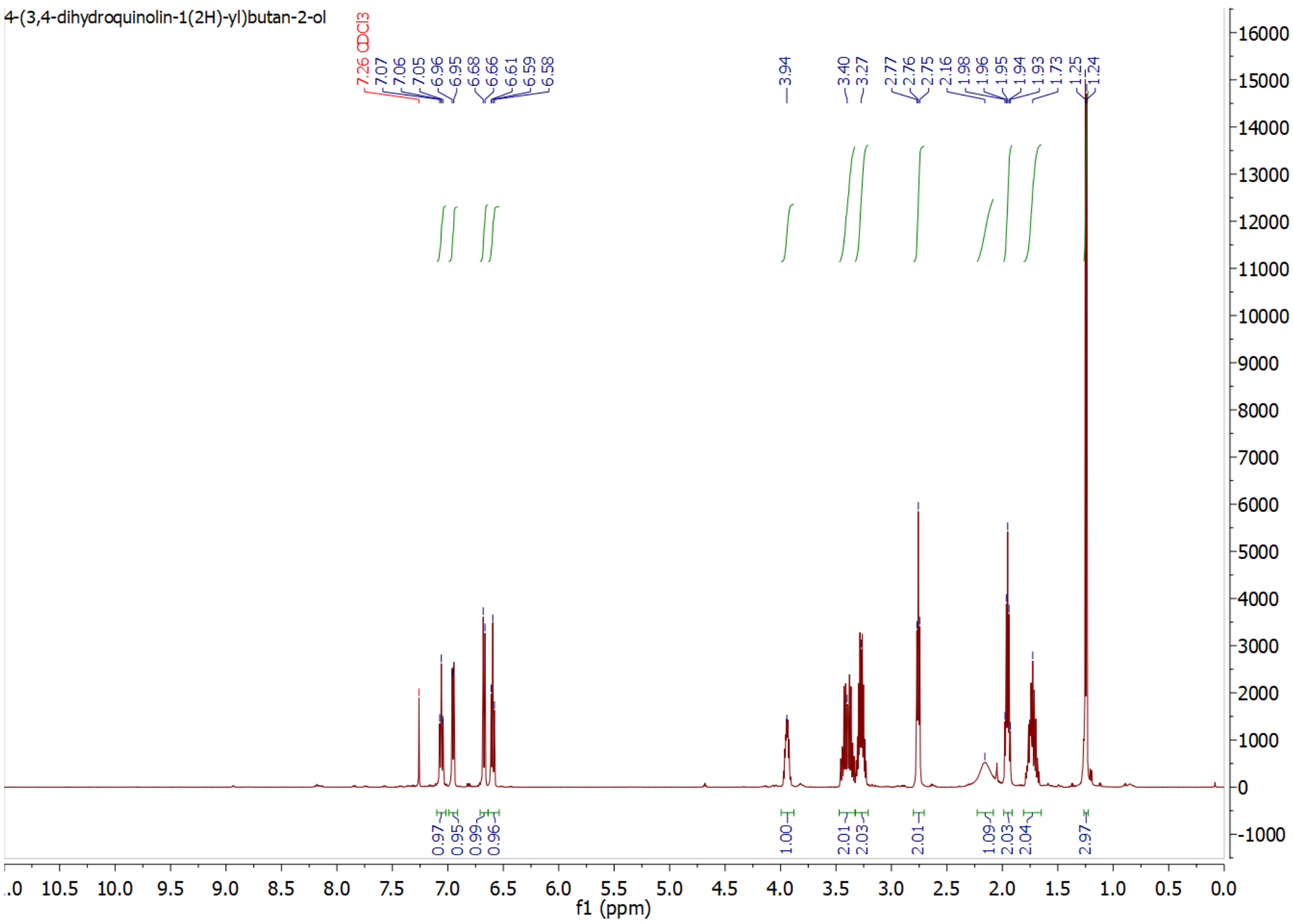

Fig. S27 ^{1}H NMR spectrum of racemic 4-(3,4-dihydroquinolin-1(2H)-yl)butan-2-ol (±)-**1a**

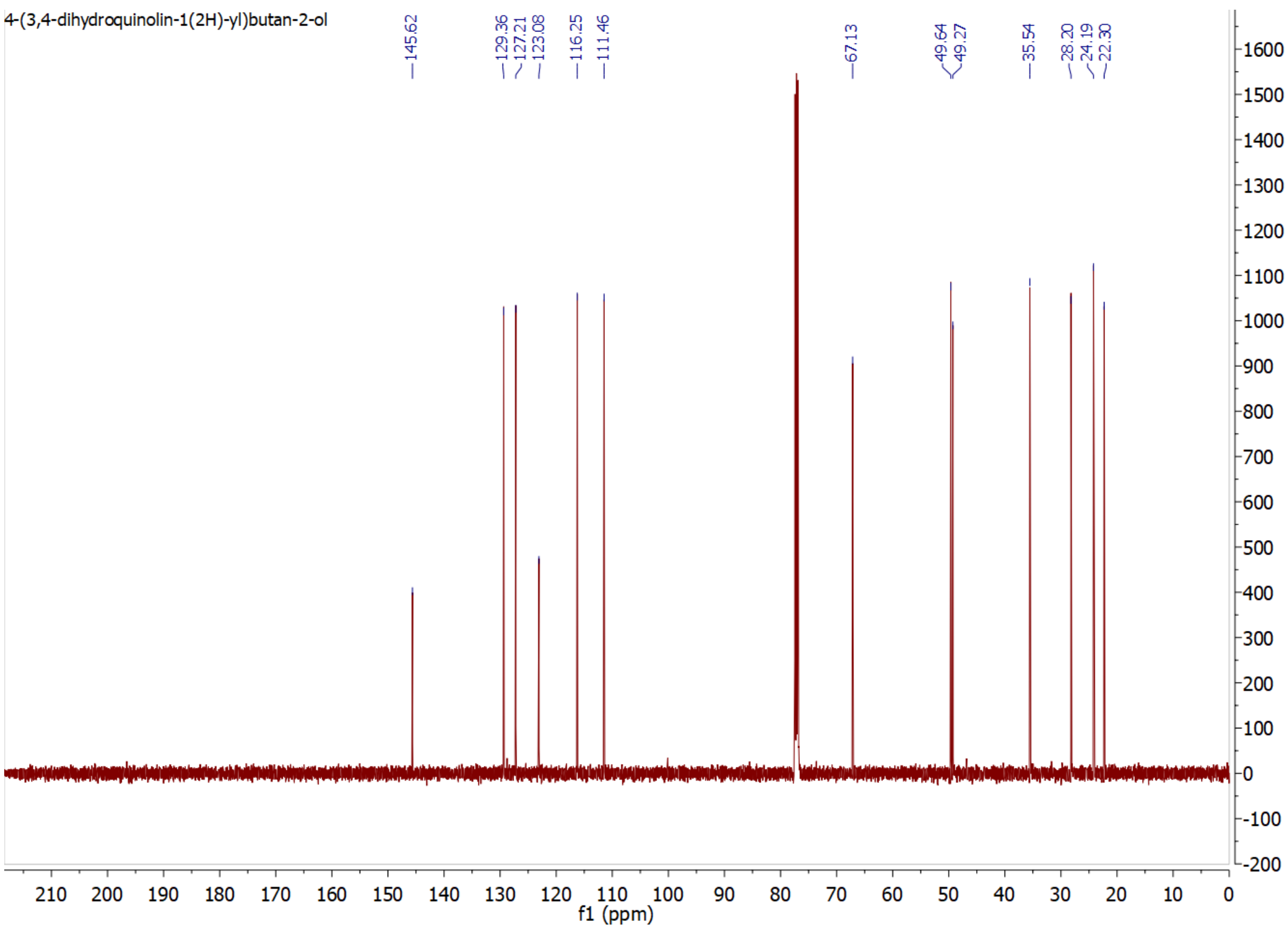

Fig. S28 ^{13}C NMR spectrum of racemic 4-(3,4-dihydroquinolin-1(2H)-yl)butan-2-ol (±)-**1a**

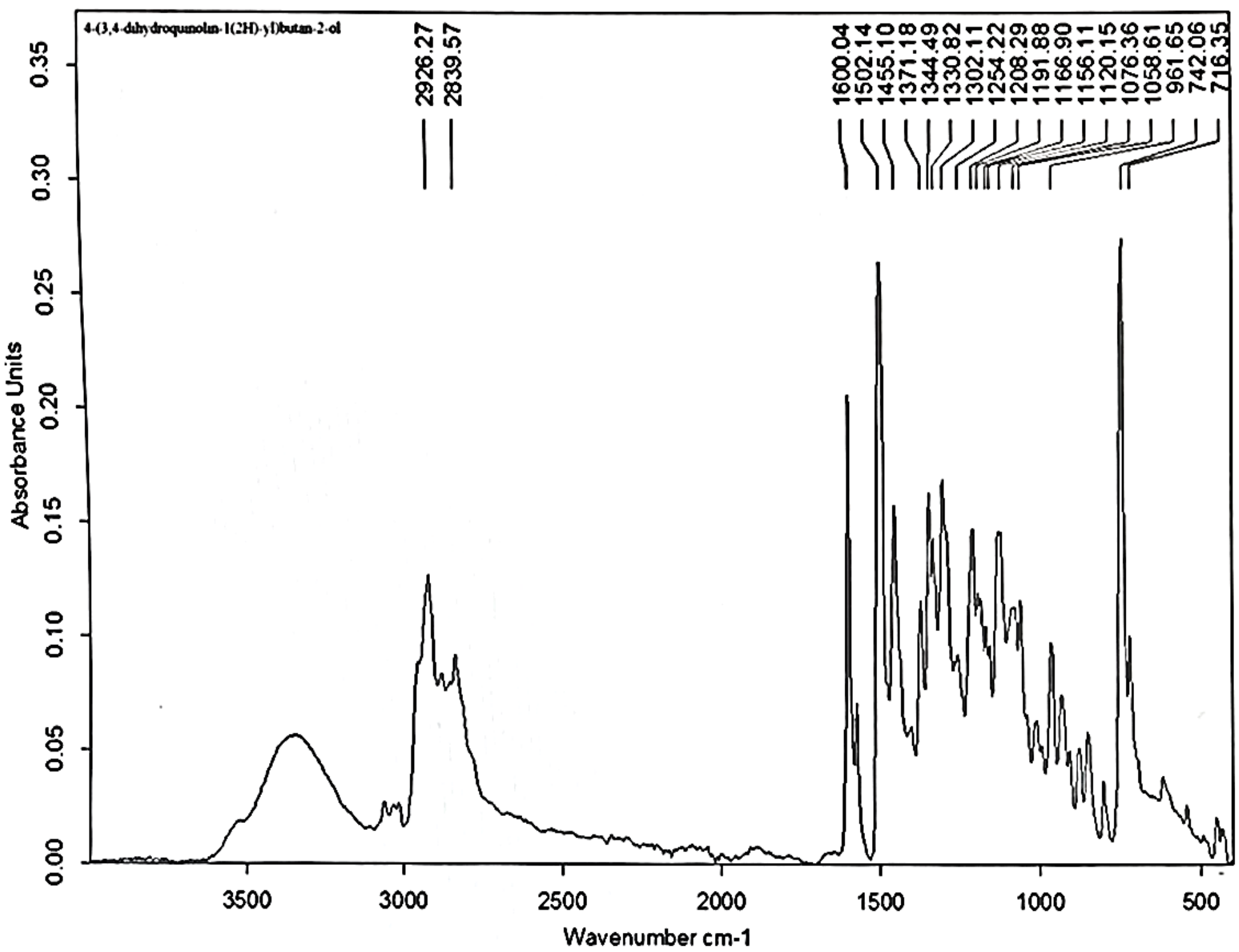

Fig. S29 IR spectrum of racemic 4-(3,4-dihydroquinolin-1(2H)-yl)butan-2-ol (±)-**1a**

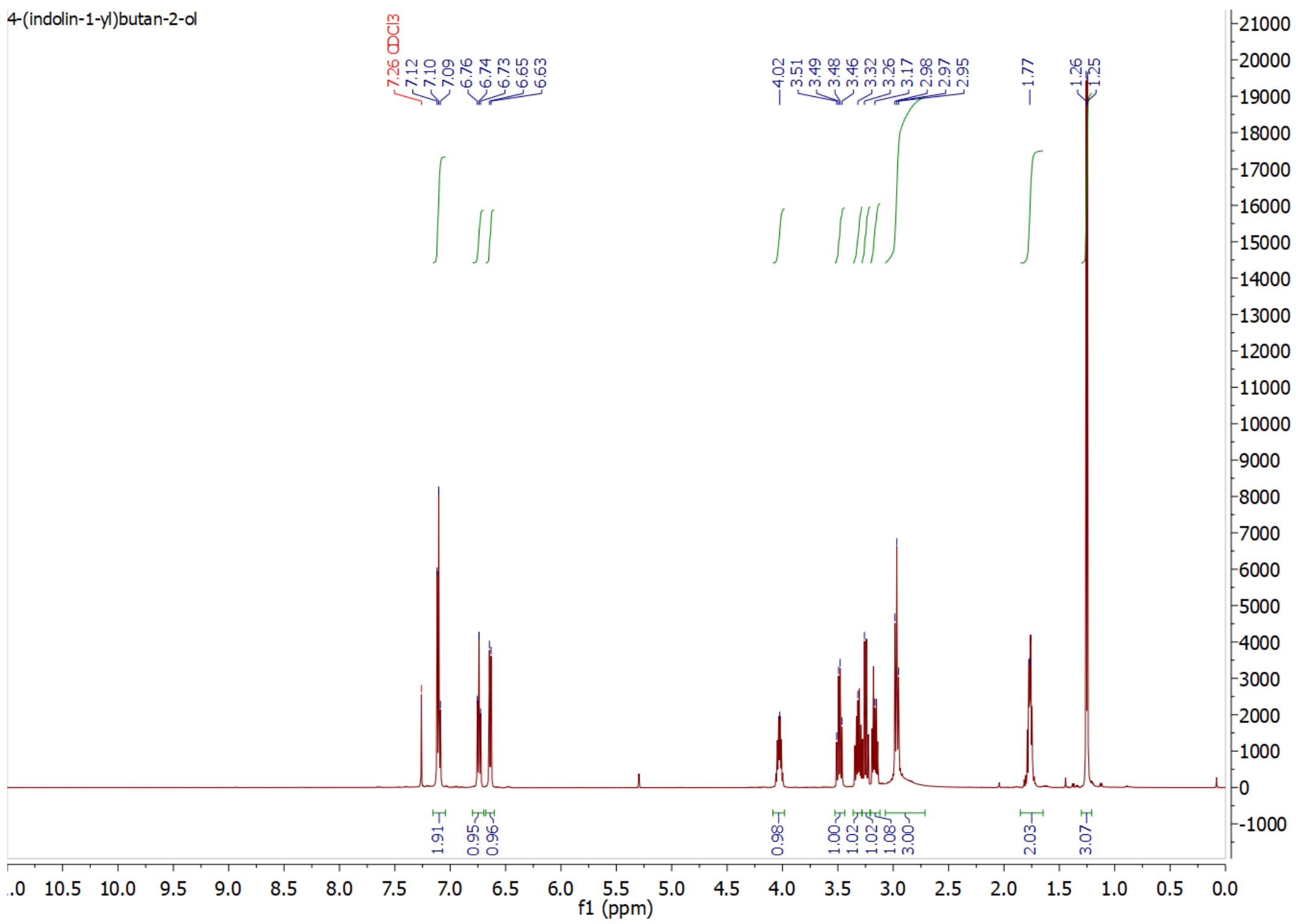

Fig. S30 ^{1}H NMR spectrum of racemic 4-(indolin-1-yl)butan-2-ol (±)-**1b**

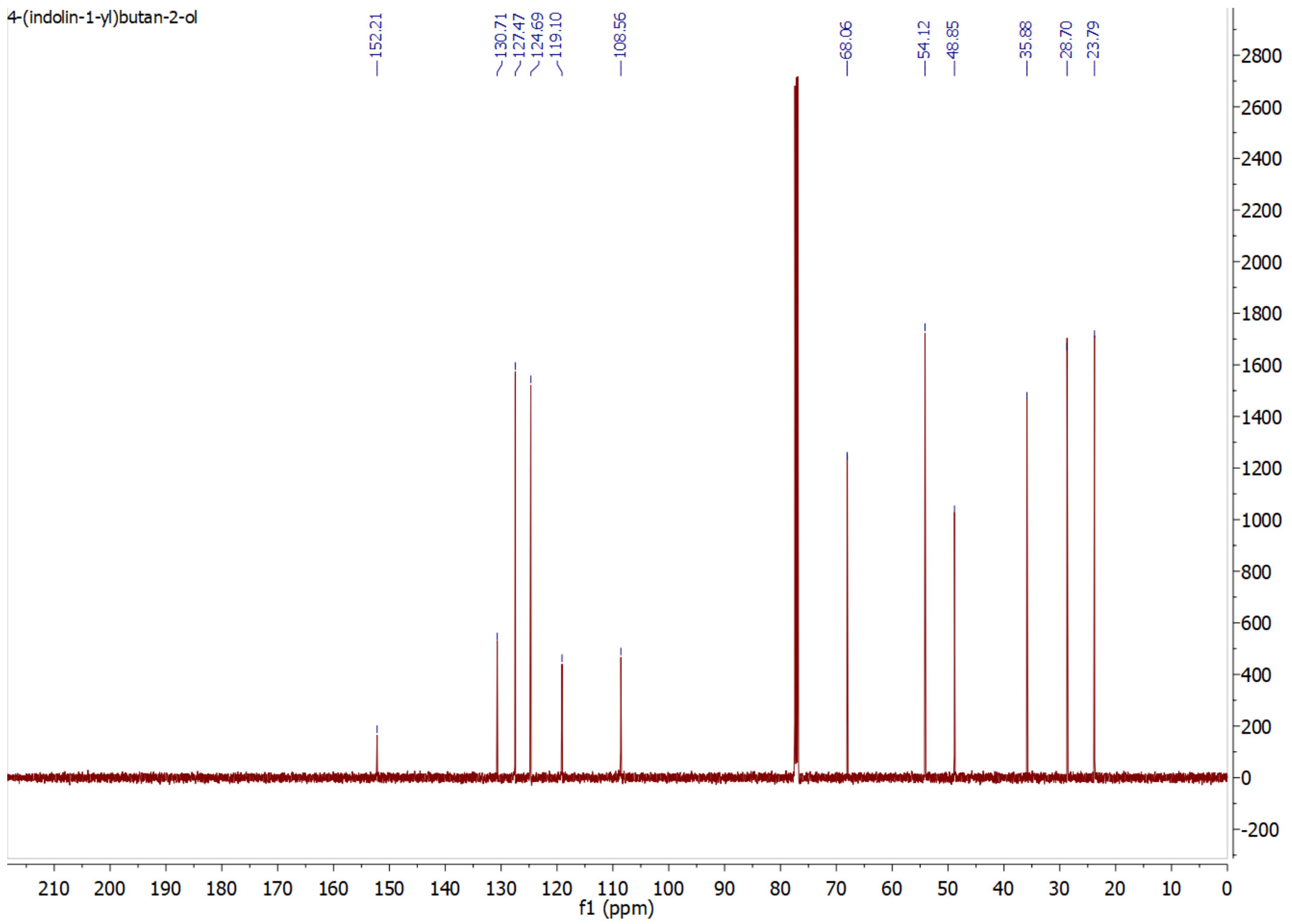

Fig. S31 ^{13}C NMR spectrum of racemic 4-(indolin-1-yl)butan-2-ol (±)-**1b**

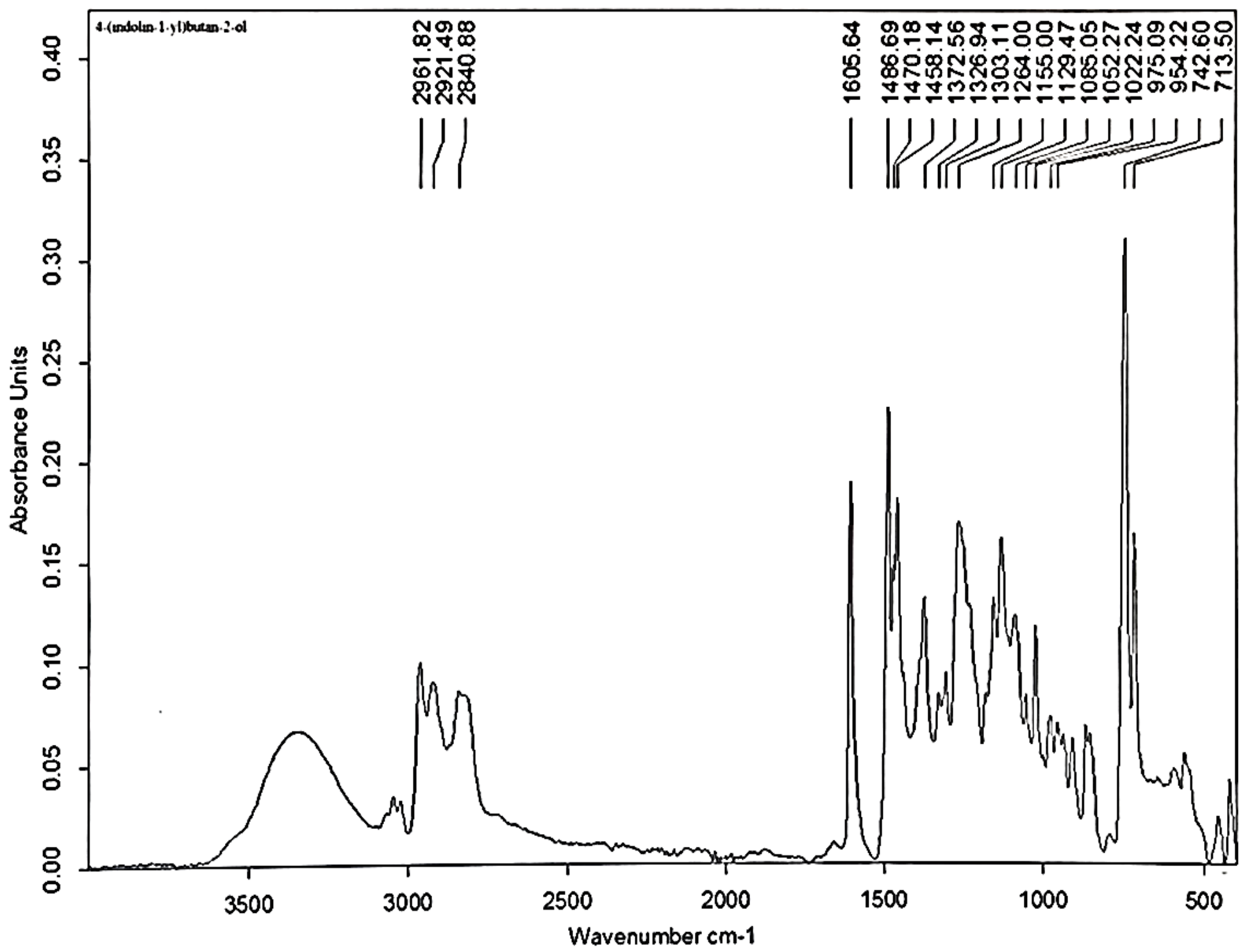

Fig. S32 IR spectrum of racemic 4-(indolin-1-yl)butan-2-ol (±)-**1b**

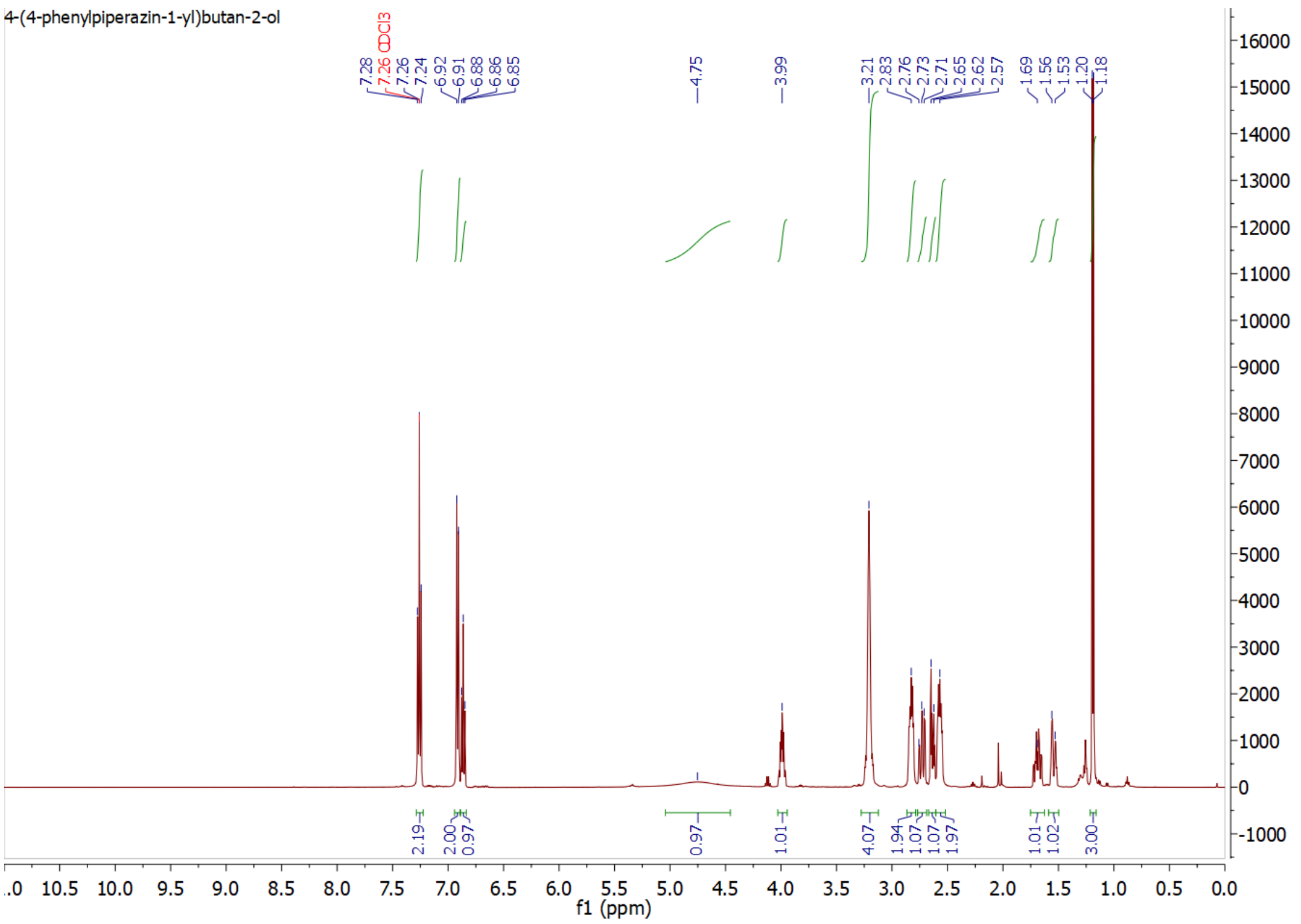

Fig. S33 ^{1}H NMR spectrum of racemic 4-(4-phenylpiperazin-1-yl)butan-2-ol (±)-**1c**

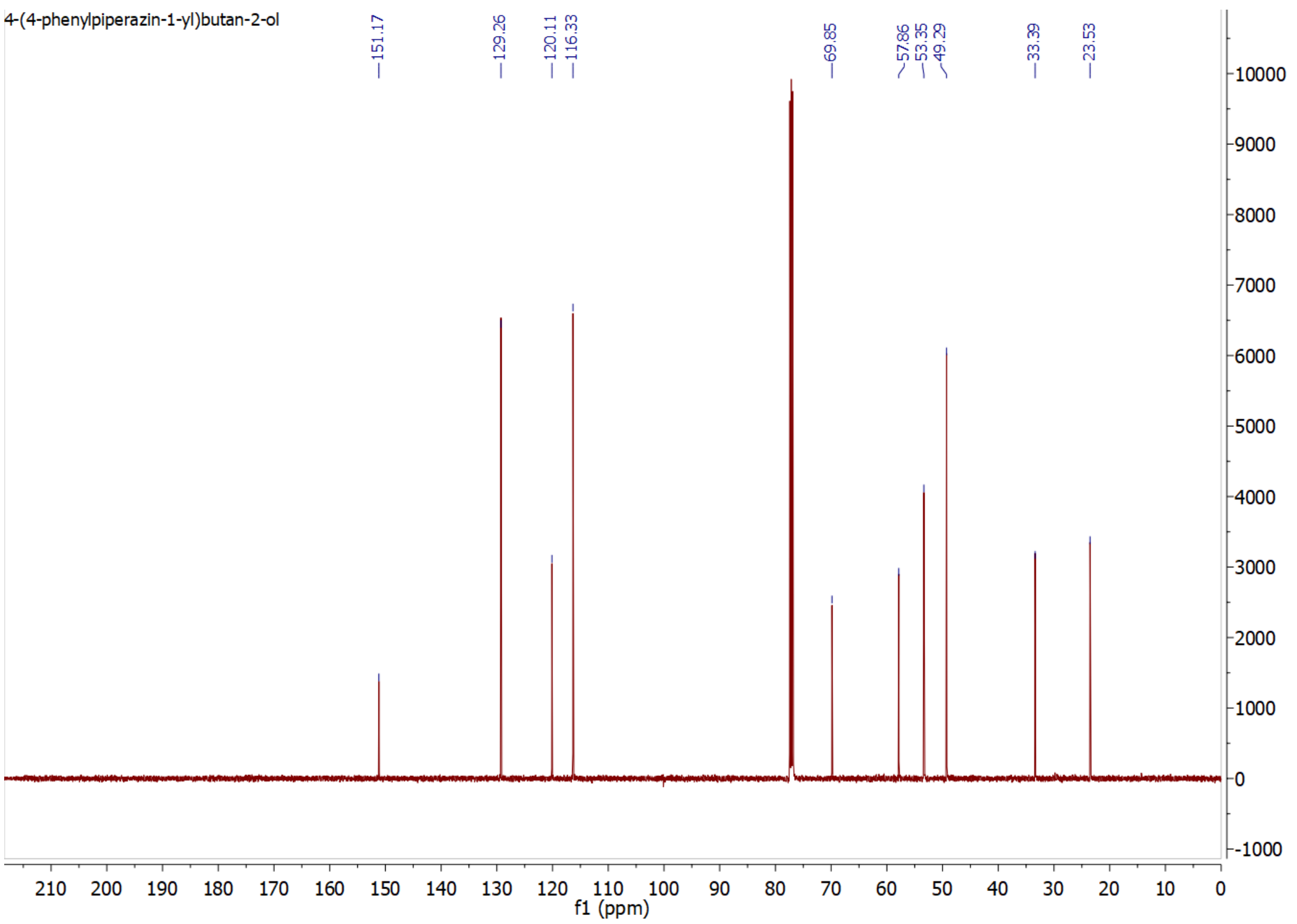

Fig. S34 ^{13}C NMR spectrum of racemic 4-(4-phenylpiperazin-1-yl)butan-2-ol (±)-**1c**

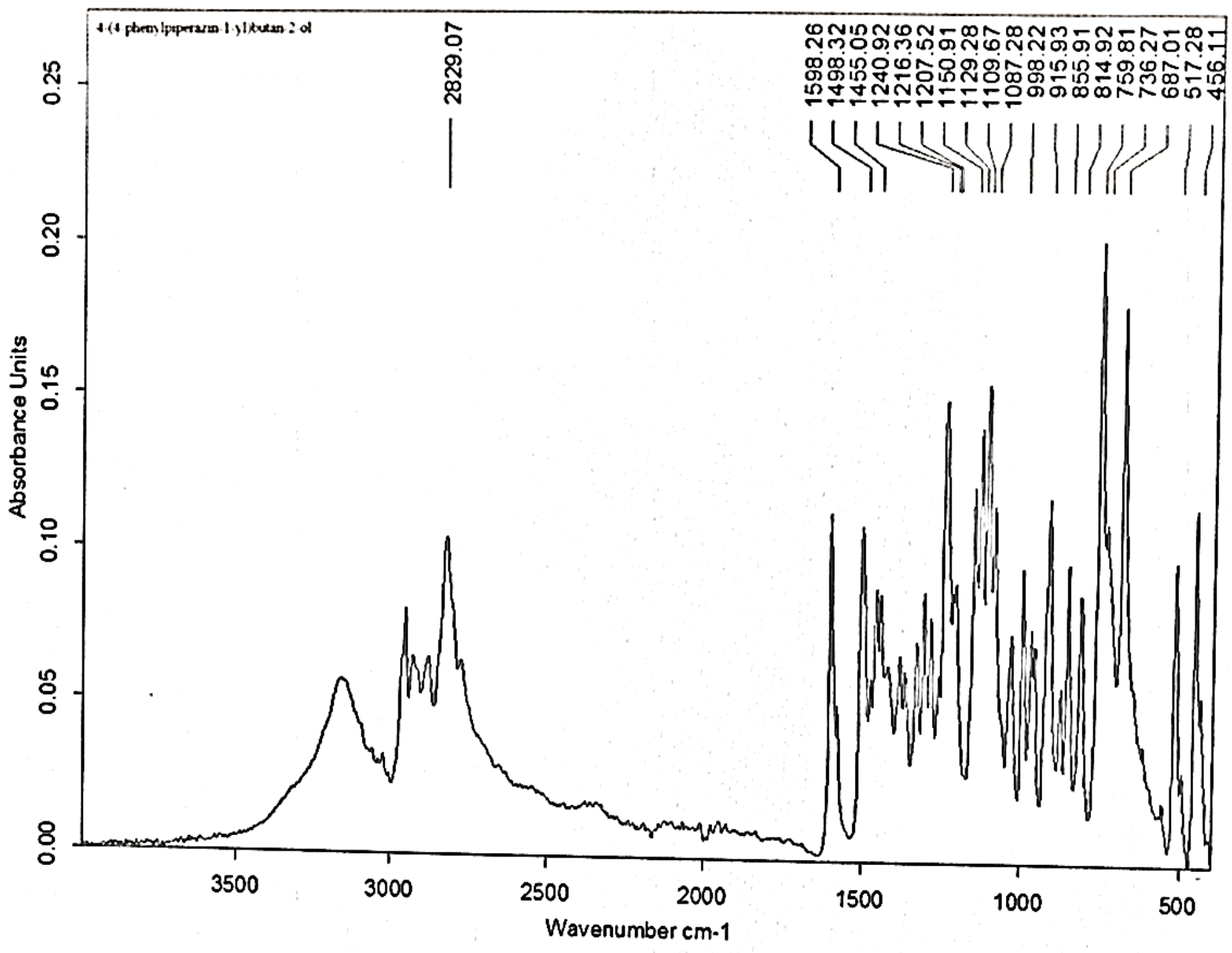

Fig. S35 IR spectrum of racemic 4-(4-phenylpiperazin-1-yl)butan-2-ol (±)-**1c**

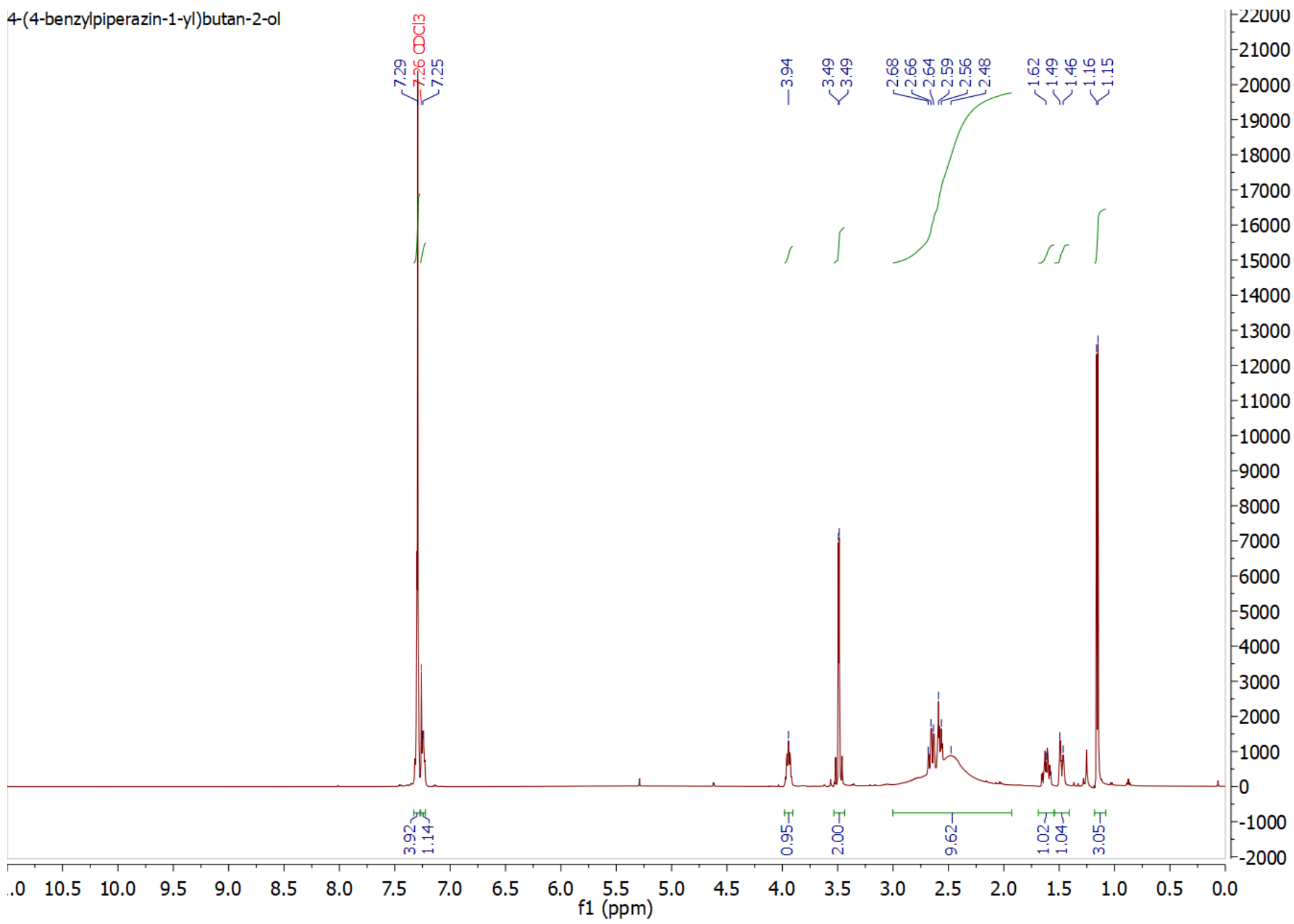

Fig. S36 ^{1}H NMR spectrum of racemic 4-(4-benzylpiperazin-1-yl)butan-2-ol (±)-**1d**

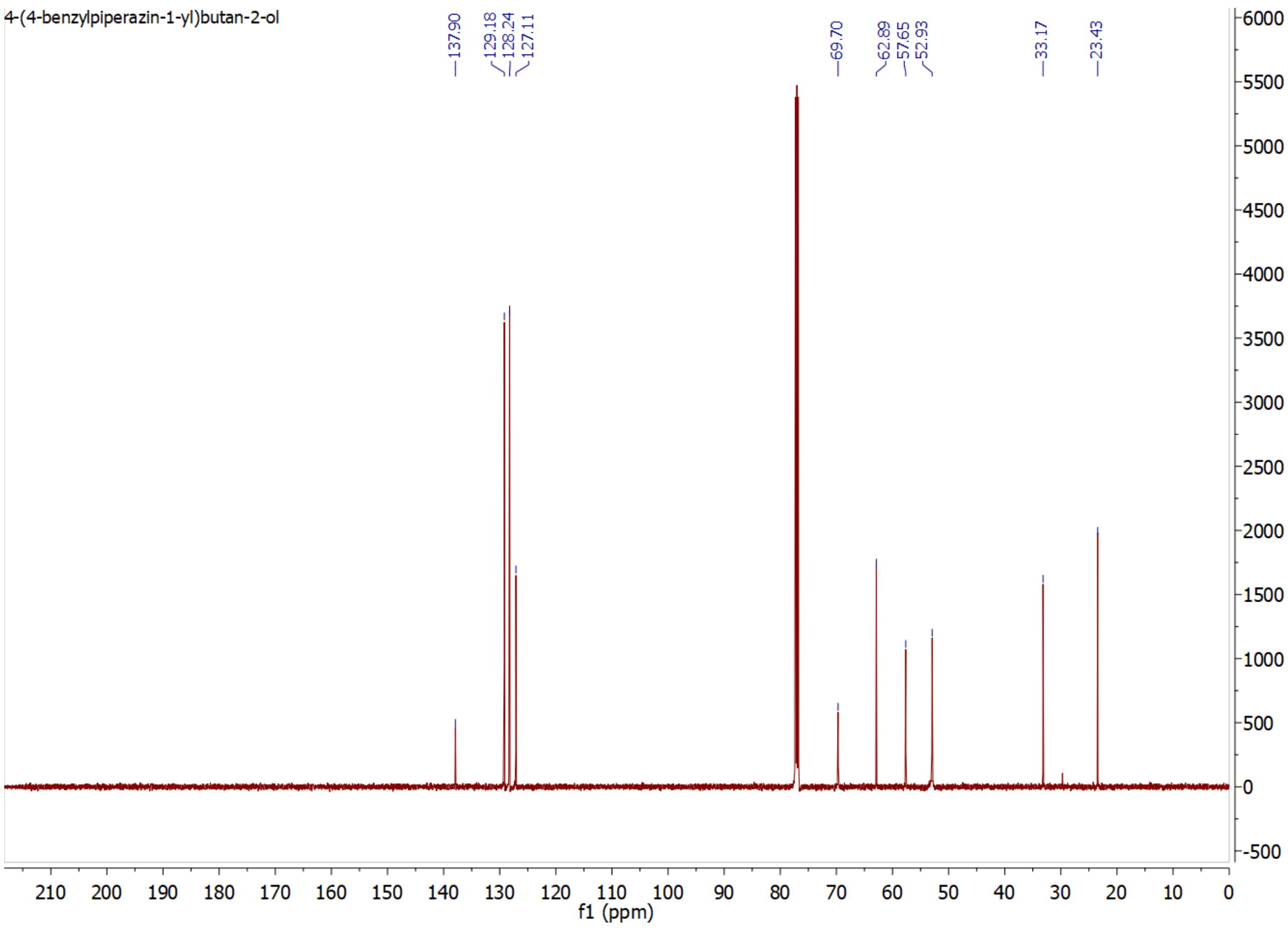

Fig. S37 ^{13}C NMR spectrum of racemic 4-(4-benzylpiperazin-1-yl)butan-2-ol (±)-**1d**

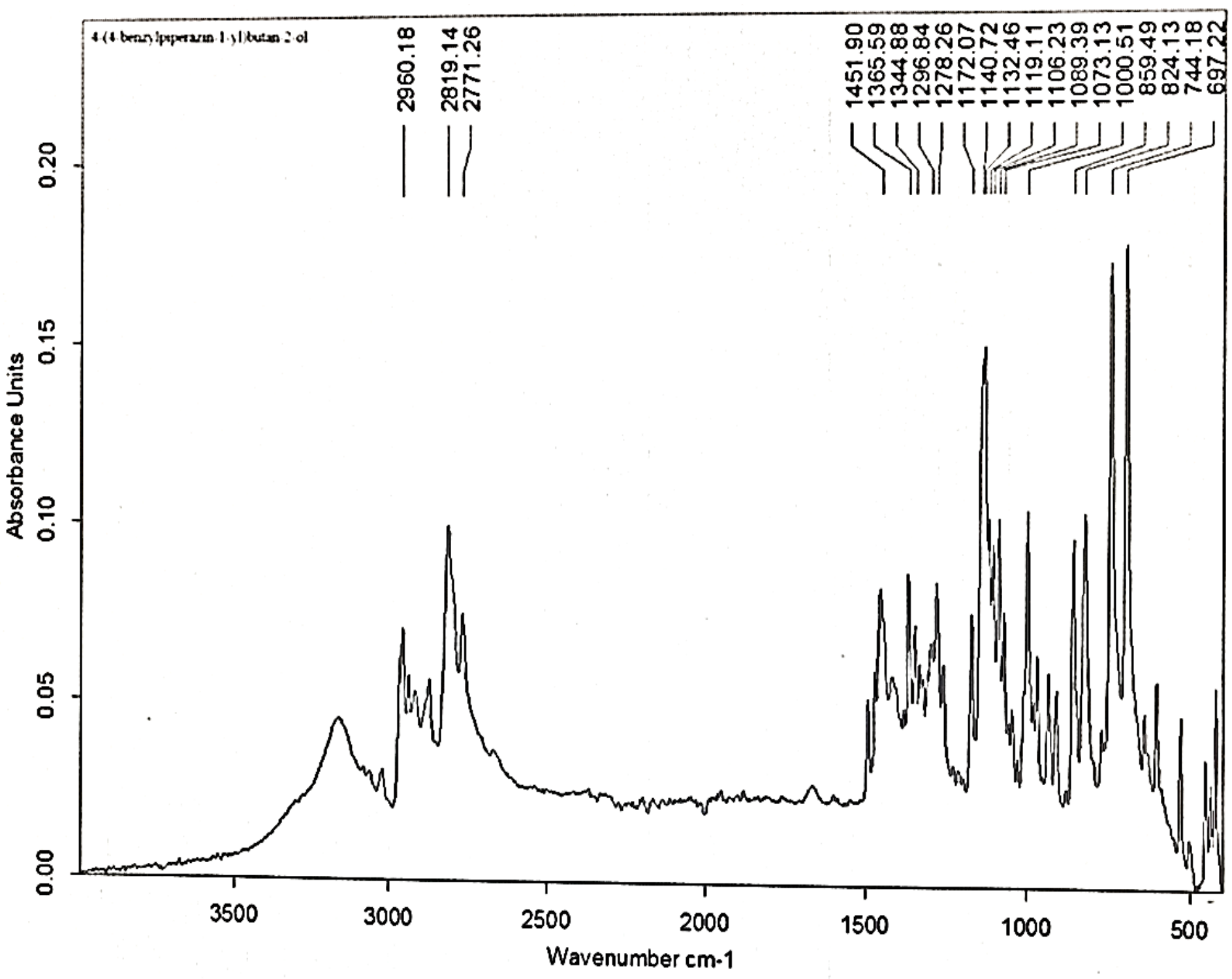

Fig. S38 IR spectrum of racemic 4-(4-benzylpiperazin-1-yl)butan-2-ol (±)-**1d**

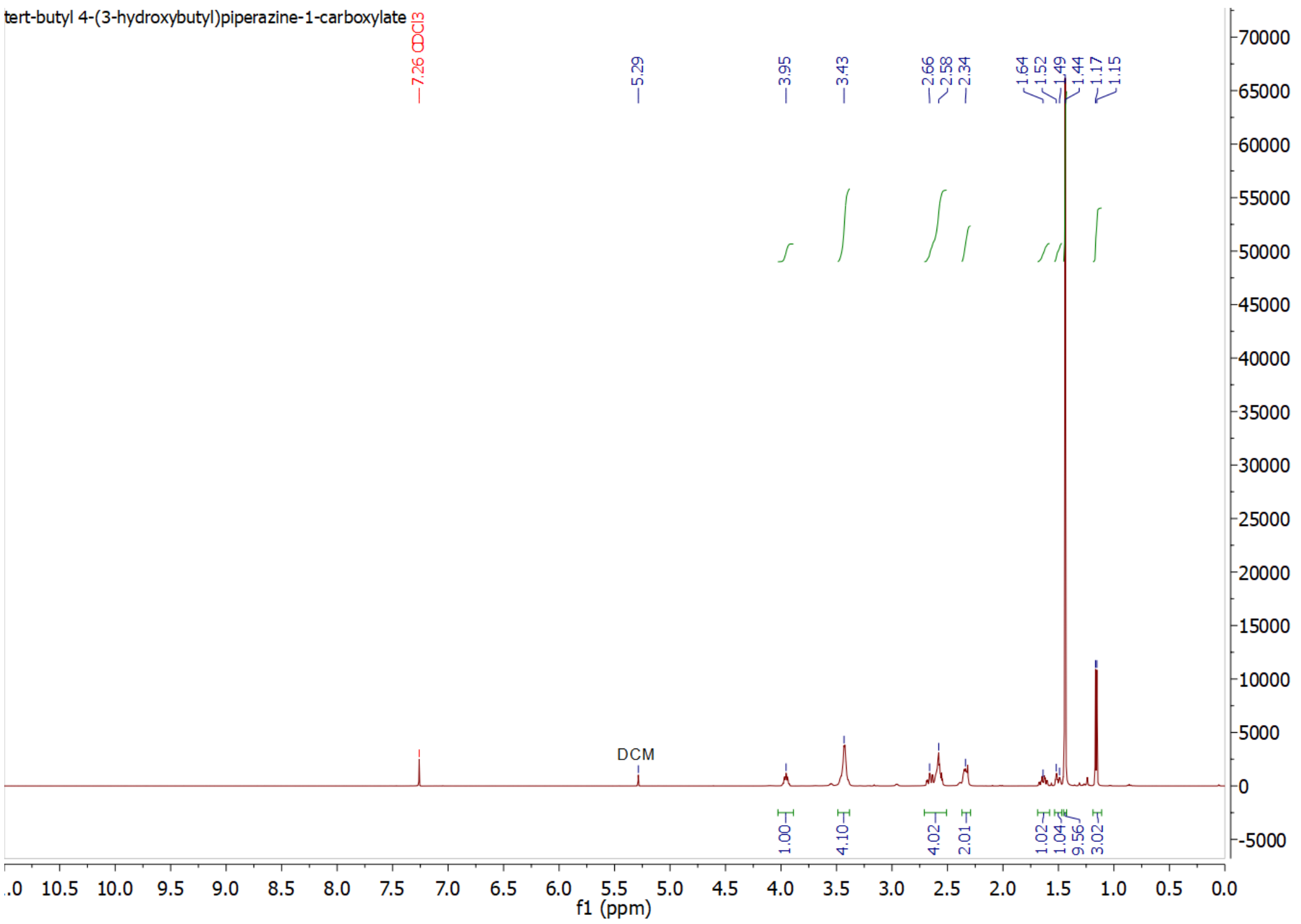

Fig. S39 ^{1}H NMR spectrum of racemic *tert*-butyl 4-(3-hydroxybutyl)piperazine-1-carboxylate (±)-**1e**

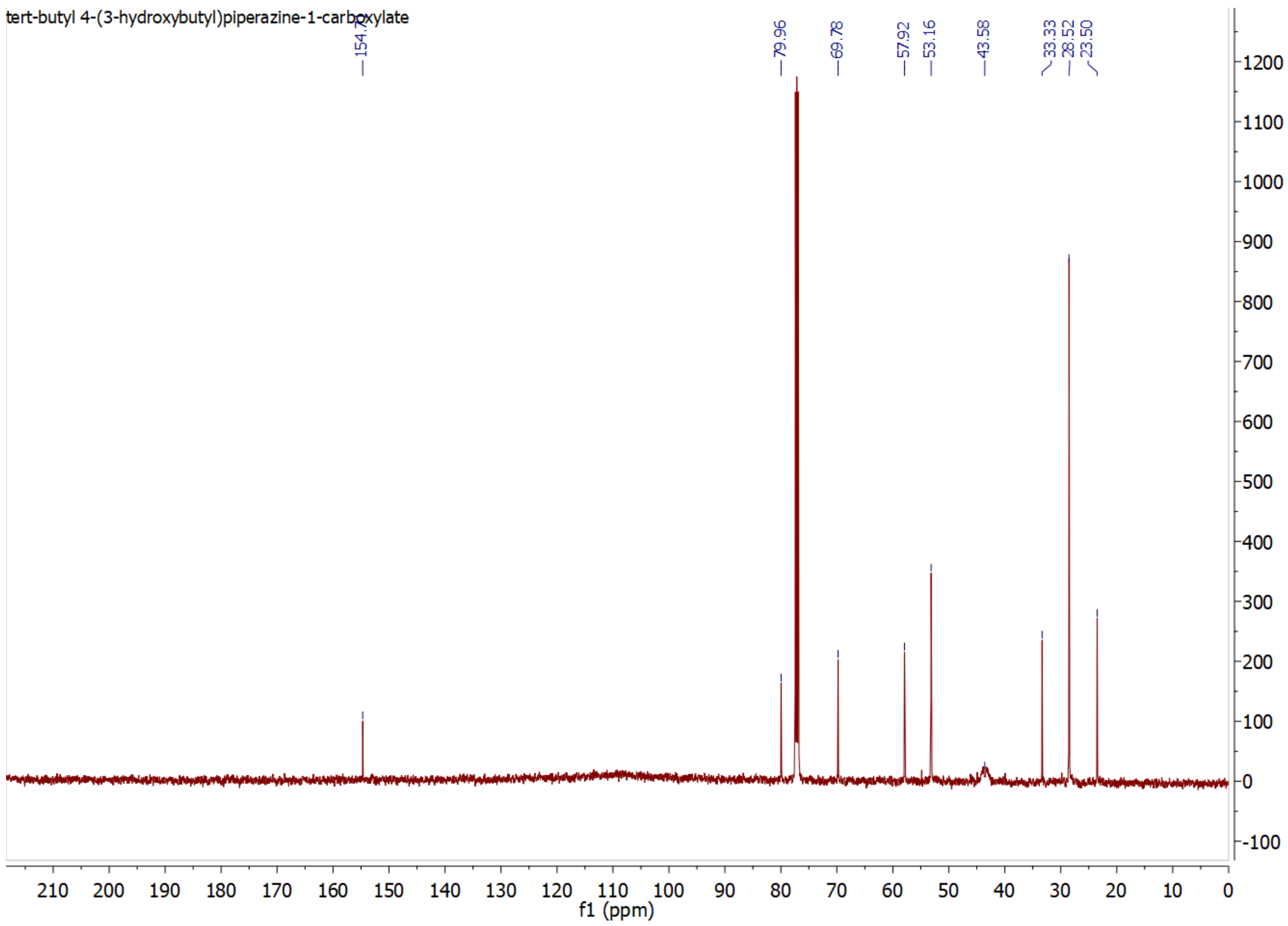

Fig. S40 ^{13}C NMR spectrum of racemic *tert*-butyl 4-(3-hydroxybutyl)piperazine-1-carboxylate (±)-**1e**

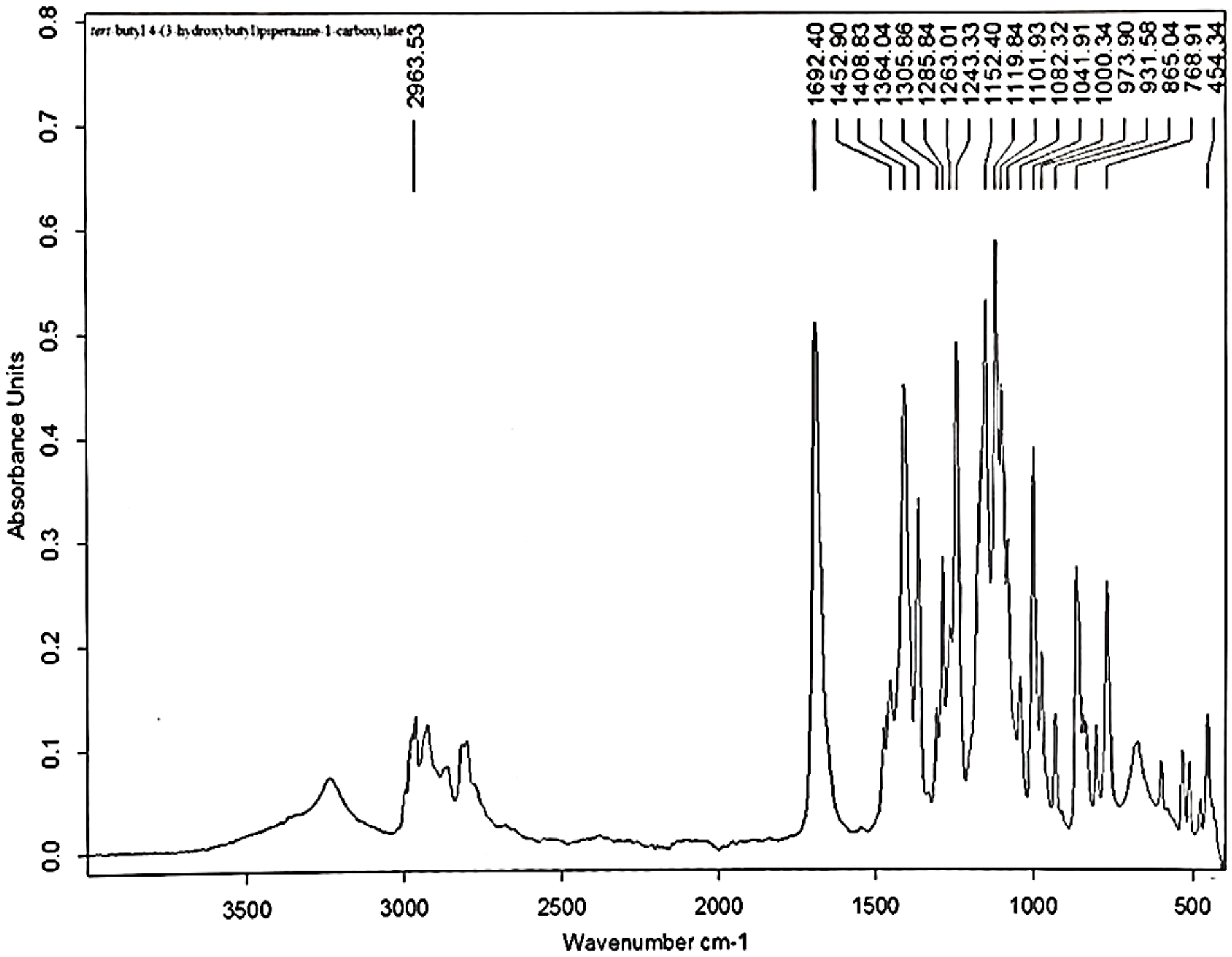

Fig. S41 IR spectrum of racemic *tert*-butyl 4-(3-hydroxybutyl)piperazine-1-carboxylate (±)-**1e**

3. References

Deb ML, Dey SS, Bento I, Barros T, Maycock CD (2013) Copper-catalyzed regioselective intramolecular oxidative α-functionalization of tertiary amines: an efficient synthesis of dihydro-1,3-oxazines. Angew Chem Int Ed 52:9791-9795. https://doi.org/10.1002/anie.201304654

DeLano WL (2002) The PyMOL molecular graphics system, 2002. (PyMOL Version 2.5.5, https://pymol.org)

Desantis J, Mammoli A, Eleuteri M, Coletti A, Croci F, Macchiarulo A, Goracci L (2022) PROTACs bearing piperazine-containing linkers: what effect on their protonation state? RSC Adv 12:21968-21977. https://doi.org/10.1039/D2RA03761K

Eberhardt J, Santos-Martins D, Tillack AF, Forli S (2021) AutoDock Vina 1.2.0: New Docking Methods, Expanded Force Field, and Python Bindings. J Chem Inform Model 61:3891–3898. https://doi.org/10.1021/acs.jcim.1c00203

HyperChem (2009) (https://www.hypercubeusa.com; Version 8.0.8)

Kilic H, Bayindir S, Erdogan E, Saracoglu N (2012) Synthesis of highly N-substituted indole library via conjugate additions of indoline and their synthetic tool potentials. Tetrahedron 68:5619-5630. https://doi.org/10.1016/j.tet.2012.04.066

Luić M, Štefanić Z, Ceilinger I, Hodošček M, Janežič D, Lenac T, Ašler IL, Šepac D, Tomić S (2008) Combined X-ray diffraction and QM/MM study of the *Burkholderia cepacia* lipase-catalyzed secondary alcohol esterification. J Phys Chem B 112:4876–4883. https://doi.org/10.1021/jp077717u

Nakamura Y, Ohta T, Oe Y (2015) A formal anti-Markovnikov hydroamination of allylic alcohols via tandem oxidation/1,4-conjugate addition/1,2-reduction using a Ru catalyst. Chem Comm 51:7459-7462. https://doi.org/10.1039/C5CC01584G

Ouyang W, Liu B, He Y, Wen Y, Gao Y, Huo Y, Chen Q, Li X (2022) Modular construction of functionalized anilines via switchable C–H and N-alkylations of traceless N-nitroso anilines with olefins. Org Chem Front 9:2746-2752. https://doi.org/10.1039/D2QO00389A

Rosignoli S, Paiardini A (2022) DockingPie: a consensus docking plugin for PyMOL. Bioinformatics 38:4233–4234. https://doi.org/10.1093/bioinformatics/btac452 (DockingPie (Version 1.2.1))

Roth HJ, Mühlenbruch B (1970) Bildungstendenz symmetrischer bis-Mannich basen des piperazins. Arch Pharm 303:156-170. https://doi.org/10.1002/ardp.19703030210

Showalter SA, Brüschweiler R (2007) Validation of Molecular Dynamics Simulations of Biomolecules Using NMR Spin Relaxation as Benchmarks: Application to the AMBER99SB Force Field. J Chem Theory Comput, 3:961–975. https://doi.org/10.1021/ct7000045

Silva FMWG, Imarah AO, Takács O, Tuba L, Poppe L (2023) Scalability of U-shape magnetic nanoparticles-based microreactor–lipase-catalyzed preparative scale kinetic resolutions of drug-like fragments. Catalysts 13:384-400. https://doi.org/10.3390/catal13020384

Trott O, Olson AJ (2010) AutoDock Vina: improving the speed and accuracy of docking with a new scoring function, efficient optimization, and multithreading. J Comput Chem 31:455–461. https://doi.org/10.1002/jcc.21334

Xu R, Wang K, Liu H, Tang W, Sun H, Xue D, Xiao J, Wang C (2020) Anti-Markovnikov hydroamination of racemic allylic alcohols to access chiral γ-amino alcohols. Angew Chem Int Ed 59:21959-21964. https://doi.org/10.1002/anie.202009754

Zhu X, Sun J, Wu Z, Guo D, Zhou S, Wang S (2024) Anti-Markovnikov hydroamination and hydroalkoxylation of allylic alcohols promoted by a simple rare-earth metal trialkyl complex. Adv Synth Catal 366:1405-1411. https://doi.org/10.1002/adsc.202301142
